# Can linear transportation infrastructure verges constitute a habitat and/or a corridor for vascular plants in temperate ecosystems? A systematic review

**DOI:** 10.1186/s13750-024-00328-3

**Published:** 2024-03-16

**Authors:** Hugo Mell, Vinciane Fack, Louise Percevault, Sylvie Vanpeene, Yves Bertheau, Aurélie Coulon, Frédérique Flamerie de Lachapelle, Eric Guinard, Arzhvaël Jeusset, Eric Le Mitouard, Dakis-Yaoba Ouédraogo, Marianne Vargac, Romain Sordello, Yorick Reyjol, Julien Touroult, Sébastien Filoche, Frédéric Hendoux

**Affiliations:** 1 Conservatoire Botanique National du Bassin Parisien (CBNBP)-Muséum National d’Histoire Naturelle (MNHN), 75005 Paris, France; 2 PatriNat, OFB-MNHN-CNRS-IRD, 75005 Paris, France; 3 Institut National de Recherche Pour l’Agriculture, l’Alimentation et l’Environnement (INRAE Centre PACA), 13182 Aix-en-Provence, France; 4 Centre d’Ecologie et de Sciences de la Conservation (CESCO), Muséum National d’Histoire Naturelle, Centre National de la Recherche Scientifique, Sorbonne Université, CP 135, 57 Rue Cuvier, 75005 Paris, France; 5 CEFE, Univ Montpellier, CNRS, EPHE, IRD, Montpellier, France; 6 University of Bordeaux, 33405 Talence, France; 7 Centre d’études et d’Expertises sur les Risques, l’Environnement, la Mobilité et l’Aménagement (Cerema), Sud-Ouest, Saint-Médard-en-Jalles, France

**Keywords:** Green infrastructure, Tracheophytes, Movement, Right of way, Roadside, Waterway bank, Pipeline, Powerline, Railways embankment, Invasion

## Abstract

**Background:**

Linear transportation infrastructures (roads, railways, oil and gas pipelines, powerlines and waterways) are recognized as important contributors to the fragmentation of species habitats. On the other hand, verges of linear transportation infrastructures (road and railway embankments, strips of grass under power lines or above buried pipelines, or waterway banks) form vast networks of continuous habitats. While the loss of natural habitats still poses a significant threat to biodiversity, verges can provide habitats or corridors in anthropogenic areas, although this potential for conservation remains controversial. The current paper is the first synthesis of evidence addressing this topic for vascular plants (except strictly aquatic species) in temperate ecosystems. We asked the following question: can linear transportation infrastructure verges constitute habitats and/or corridors for vascular plants in temperate ecosystems?

**Methods:**

We conducted a systematic literature survey using two online bibliographic databases, three search engines, specialist websites, and by sending a call for literature to subject experts. We also integrated studies from a previous systematic review with an overlapping scope. We successively screened the articles for relevance on titles, abstracts and full texts using criteria detailed in an a priori protocol. We then used six specific questions to categorize the selected studies and critically assess them. These questions encompassed the potential of verges as habitats and corridors for vascular plants, and the effects of landscape and management on these potentialities. We created a database of the studies with low and medium risk of bias. We synthesized results for specific questions in narrative syntheses. Finally, studies about the habitat role of verges that met the criteria for a meta-analysis were used for quantitative syntheses.

**Review findings:**

Our systematic literature survey yielded 101,524 search results. After critical appraisal, we included in our systematic review 294 articles that reported 316 studies. Most studies were conducted along road verges or waterway banks, with only a handful of studies involving powerlines, railways or pipelines. We were not able to draw conclusions on the role of verges as corridors for vascular plants as too few relevant studies were obtained. Regarding the habitat function of verges however, meta-analyses were conducted based on 205 cases from 47 primary studies that compared abundance and/or species richness in verges vs habitats away from transportation infrastructure for exotic, native or all species together. For non-highway road verges, both the abundance and richness of exotic species were higher on non-highway road verges, but we found no significant differences among species in general, or for native species specifically, which implies that alien species would often add but not subtract species. A wide variety of management practices were also represented in the evidence base. Overall, systematic impacts on species richness or abundance rarely emerged, but human interventions were seldom neutral and usually altered, at least temporarily, the balance between the native and exotic flora or among various functional groups.

**Conclusions:**

We identified a major knowledge gap regarding the potential of linear transportation infrastructure verges as corridors for vascular plants. Thus, we call for more research on this particular topic, especially as the evidence synthesis underlined the potential of verges as habitat for exotic and invasive flora.

**Supplementary Information:**

The online version contains supplementary material available at 10.1186/s13750-024-00328-3.

## Background

For the last decades, human activities have resulted in a global loss of biodiversity [1], and activities related to transportation were identified as one of the ten major threats faced by threatened or near-threatened species [2]. Linear transportation infrastructures (LTIs) led to habitat loss and degradation, fragmentation and barrier effects, light and noise disturbance, chemical pollution and direct mortality (e.g. road kill, electrocution) [3–8]. In particular, fragmentation (i.e. the splitting of natural habitats and ecosystems into smaller and more isolated patches) and the associated loss of habitat were identified to have significant negative effects on biodiversity [9]. LTIs induced a decrease in wildlife species abundance at local and large scales [10], and, through barrier effects, usually restrict wildlife movements, disrupt gene flow and metapopulation dynamics, leading to the genetic isolation of populations (e.g. [11]*.*).

Considered transversally, LTIs thus generate a demonstrated negative fragmenting effect on biodiversity. Considered longitudinally, LTIs have the potential to constitute a habitat and/or movement corridors for biodiversity by their semi-natural verges [12]. Indeed, inside the LTI boundaries there is generally a transportation lane (road, railway, pipeline, powerline, river or canal) surrounded by verges which are usually covered with vegetation: road and railway embankments, strips of grass under power lines, waterway banks or above buried pipelines that need forest cutting or scrub clearance. Studies assessing the potential of LTI verges as habitat and/or corridor for wildlife species have provided contrasted results. For instance, road verges sometimes contain significant portions of the remaining populations of rare plant species [13, 14]. On the other hand, the alteration of the original habitat induced by the construction of transport infrastructure can favour the establishment and spread of exotic plants (e.g. [15, 16]*.*). The potential of LTI verges as habitat and/or corridor for wildlife species may also vary with verge management practices, as one practice (e.g. mowing) can be beneficial for some species (e.g. disturbance-tolerant species) but not for others (e.g. woody species) [17, 18].

Knowing the role of LTI verges as habitat and/or corridor for biodiversity is of importance as verges might contribute to ecological networks. In the last decades, ecological networks of terrestrial and aquatic continuities (blue-green infrastructures) aiming to decrease fragmentation have received much attention from scientists and policymakers [19]. Meta-analyses of corridor effectiveness showed that, overall, corridors increase movements of plants, vertebrates and invertebrates between habitat patches, but that corridor effectiveness varies among taxa [20, 21]. Maintaining such a network of ecological corridors might be beneficial in the long term in the context of climate change by facilitating species dispersal to newly suitable areas [22]. In France, the concept of green and blue infrastructures led to the development of a public policy named “Trame Verte et Bleue” (meaning green and blue ecological network) launched by the French Ministry of Ecology in 2007. Accordingly, French administrative regions identified ecological networks and they conduct action plans for preserving and restoring these corridors for the benefit of biodiversity. At a smaller spatial scale, i.e. townships, corridors also have to be considered as part of local urban planning.

## Topic identification and stakeholder input

In France, the LTI network is very dense. For instance, the road network is the longest (over a million kilometres long, ¼ of the European network) and one of the densest (1.77 km/km^2^) of the European Union. As a comparison, Spain, which is similar in size to France, has a road density six times lower (0.32 km/km^2^). The railway network is also one of the longest in Europe with more than 30,000 kms of railway lines in use. Thus, such a dense LTI network means a considerable inherent surface of verges and LTI managers might substantially contribute to ecological networks. Seminal reviews on this question were previously published (e.g. [8, 17, 23]*.*) but they focused on roads and do not fulfil the standards of a systematic review [24]. This situation motivated several French LTI managing companies and the French Ministry of Ecology to request a systematic review on this issue, central to the green and blue infrastructure public policy.

The French LTI managing companies are gathered in an informal group, named “Club des Infrastructures Linéaires & Biodiversité” (CILB), aiming at acting for biodiversity conservation. The motorway, railway, power line, pipeline and waterway French stakeholder companies who are members of the CILB were specifically interested in evaluating whether their LTI verges could contribute to green and blue infrastructures and to improve the management of these verges for that purpose. The systematic review was assumed to be a relevant scientific method to provide a sound answer to this practical questioning from LTI managers. A call for tender for a systematic review was thus launched by the French Ministry of Ecology and the French Agency for Environment and Energy Management (ADEME) through its research incentive program related to transportation ecology, named “Infrastructures de Transport Terrestre, Écosystèmes et Paysage” (ITTECOP), supported by the CILB and the “Fondation pour la Recherche sur la Biodiversité” (FRB), a French foundation supporting research in biodiversity. At the beginning of the project, the LTI managers funding the study were met to list the types of verges they own and the management practices they apply on those, to define the components of the review question.

The protocol of the systematic review was published in 2016 [25]. Because the question encompasses all biodiversity, a very large number of articles were collected. The review process was thus split by taxa in three stages. A first systematic review focusing on insects was published in 2018 [26]. A second systematic review on vertebrates (mammals, birds, amphibians and reptiles) was published in 2020 [27]. Regarding insects, our systematic review revealed that their abundance was generally not statistically different between LTI verges and away from LTIs. Insect abundance was even higher on non-highway road verges than away from roads. Regarding vertebrates, highway verges had higher abundance of small mammals but both lower abundance and species richness of birds than away from highways. The opposite pattern was found however for bird species richness and abundance along waterways. The aim of the present work was to carry out the third systematic review initially planned in this project, focused on vascular plants (i.e. Tracheophyta).

## Objective of the review

The general aim of the review was to determine if LTI verges can provide habitats or corridors for all vascular plants except strictly aquatic species. This work exclusively focused on the longitudinal effect of LTI verges without considering the transversal effect of LTIs, such as barrier effects. The review also aimed to assess the effects of managements practices (e.g. mowing), as well as the characteristics of the surrounding landscape, on the potential of LTI verges for vascular plants.

### Primary question

The primary question of the review was: “can linear transportation infrastructure verges constitute habitats and/or corridors for vascular plants in temperate ecosystems?”.

### Secondary questions

The approach in this review consisted in splitting the above primary question into six specific questions detailed in Tables 1 and 2. This subdivision was used during study validity assessment and the synthesis of evidence.

**Table 1:** Details of the six specific questions of the review

Number	Details
Question Q1	Do LTI verge management practices increase, decrease or have no effect on tracheophyte biodiversity in LTI verges?
Question Q2	Is tracheophyte biodiversity in LTI verges equal to, higher, or lower than in similar habitats away from LTIs?
Question Q3	Do LTI verge management practices increase, decrease or have no effect on tracheophyte dispersal in LTI verges?
Question Q4	Is tracheophyte dispersal in LTI verges equal to, higher, or lower than their movements in similar habitats away from LTIs?
Question Q5	Is tracheophyte biodiversity in LTI verges dependent on the surrounding landscape?
Question Q6	Is tracheophyte dispersal in LTI verges dependent on the surrounding landscape?

**Table 2:** PECO and PICO elements of the systematic review

Population (Q1–Q6)	All vascular plant species (except strictly aquatic plants) and communities
Exposure (Q2–Q4–Q6)	LTI verges; i.e. road, railway, powerline or pipeline verges and waterway banks. Regarding the latter, as our systematic review focused on LTIs, we only considered navigable waterways (navigable rivers and canals) as relevant exposures
Comparator (Q2–Q4–Q6)	Temporal and/or spatial comparators, including but not restricted to habitat present before versus after infrastructure construction (LTI verge creation), LTI verge versus nearby similar habitat away from LTIs
Intervention (Q1–Q3–Q5)	Management practices (e.g. mowing) or human-induced disturbances (e.g. waterway channelization) on LTI verges
Comparator (Q1–Q3–Q5)	Temporal and/or spatial comparators, including but not restricted to LTI verge before versus after management intervention, LTI verge managed with one practice versus unmanaged LTI verge or LTI verge managed with a different practice
Outcomes (Q1–Q6)	All outcomes relating to species presence, reproduction or dispersal, including but not restricted to species richness, abundance, community composition, reproductive indicators and species dispersal

We defined LTI verges as the area up to 30 m from roadways, waterways or railways, or the area (whatever the width) below power lines or below/above pipelines. For questions related to the role of the surrounding landscape, the spatial scale considered could range from the land use directly adjacent to the LTI verge, to radii of hundreds of meters around the sampled verges.

### Components of the primary question

Population: All vascular plant species (except strictly aquatic plants) and communities.

Exposure: LTI verges; i.e. road, railway, powerline or pipeline verges and waterway banks. Regarding the latter, as our systematic review focused on LTIs, we only considered navigable waterways (navigable rivers and canals) as relevant exposures.

Intervention: Management practices (e.g. mowing) or human-induced disturbances (e.g. waterway channelization) on LTI verges.

Comparator: Temporal and/or spatial comparators, including but not restricted to habitat present before versus after infrastructure construction (LTI verge creation), LTI verge before versus after management intervention, LTI verge versus nearby similar habitat away from LTIs, LTI verge managed with one practice versus unmanaged LTI verge or LTI verge managed with a different practice.

Outcomes: All outcomes relating to species presence, reproduction or dispersal, including but not restricted to species richness, abundance, community composition, reproductive indicators and species dispersal.

Context: Because the funders requested an evidence synthesis applicable to western Europe, we restricted our synthesis to temperate zones.

## Methods

The methods are described in details in an a priori systematic review protocol [25]. It follows the Collaboration for Environmental Evidence (CEE) Guidelines and Standards for Evidence Synthesis in Environmental Management in their version in force at the time of validation of the protocol [28]. The CEE Guidelines have evolved since 2016 but, since searching and screening have been performed at an early stage, revising our methodology would have involved to restart this review from the beginning. As a consequence, due to the lack of resources, we were committed to the method validated in 2016, that will allow to maintain consistency between the three systematic reviews on insects, vertebrates and vascular plants, and thus comparisons between results. We summarized in the following section the small deviations from the protocol that we made when conducting the review.

### Deviations from the protocol

Search for articles:

- Searches on search engines were performed using search terms in English only, and not in both English and French.
- Searches on Google (https://www.google.fr) were not conducted.
- Searches on specialist websites were not performed at the beginning of the review (searches on Google Scholar and the call for literature were considered sufficient). However, the update for grey literature was conducted in 2018 through searches on specialist websites, as well as through searches on Google Scholar, BASE (https://www.base-search.net/) and CORE (https://core.ac.uk/). Yet, the call for literature was not carried out once more during this first update and only the main publication databases (WOS Core Collection and Zoological Records) were searched during the second update in 2020.

Screening:

- The consistency of reviewers’ decision during full-texts’ screening was not tested for the two first searches (2016, 2018), due to logistical and time constraints, but it was for the last update (2020).
- Although it was not clearly stated in the protocol, it is a CEE standard that articles without abstracts are assessed at full-text. However, in this review, articles without abstracts were directly excluded due to their high number and time constraints.

Data extraction:

- For better referencing of the studies, the variable “DOI” was extracted. The unique identifier of the publication, the source of the publication, and the specific question (Q1-Q6) addressed by the study were also indicated in the final table of included studies, as well as whether the study is included in the narrative synthesis and in the meta-analyses.

Narrative synthesis:

- Due to the large number of studies retained after critical appraisal, studies dealing with our specific question Q2 were not extracted for the narrative synthesis as this question could be addressed in a quantitative synthesis.

The implications of these deviations are considered in the review limitations section.

### Searching for articles

Searching for articles, whose method is detailed here, has formed a common process for all taxa (insects, vertebrates, flora) according to the systematic review protocol.

#### Search strings

The review team identified English search terms to be combined in search strings. For all keywords listed wild-cards may be used to allow the use of derivations of the word’s root and to account for the possibility of finding a word in various spellings (English from Great Britain or from the United States) and with various endings (singular or plural).

We tested a first search string combining some of the search terms with Boolean operators of Web Of Science Core Collection (with search on “Topic”). To assess the comprehensiveness of the search string, we compared the search hits to the articles of the test list indexed in the database (see Additional file 2 for the list of articles of the test list and how it was constituted). Then, we modified the search string by removing some of the search terms and including new ones, to increase the number of articles of the test list retrieved [25]. The search string that produced the highest efficiency (i.e. total number of search hits as low as possible with the highest number of articles from the test list retrieved) was a set of four sub-search strings displayed in Table 3. Of the 102 articles included in the test list, 95 were indexed on Web of Science and/or Scopus and only one of them was not retrieved by our final search string on either Web of Science or Scopus, resulting in a comprehensiveness of 99% (94/95).

**Table 3:** Sub-search strings selected and used in Web Of Science Core Collection and zoological records publications databases

LTI	Strategy	Search string
Roads, railways, pipelines and powerlines	1	*LTIs:* (“transport* infrastructure*” OR road* OR highway$ OR motorway$ OR freeway$ OR rail* OR pipeline$ OR powerline$ OR “power line” OR “power lines” OR “transmission line*” OR “electric* line” OR “electric* lines” OR “electric* pylon*”) AND *Verges/outcomes:* (corridor$ OR dispersal$ OR habitat$ OR refuge$ OR “right* of way*” OR verge$ OR abundance OR richness OR composition$ OR *diversity OR communit*)
	2	*LTIs:* (road* OR highway* OR motorway* OR rail* OR “transmission line* corridor*” OR powerline* OR pipeline* OR “electric* pylon*”) AND *Verges:* (corridor* OR habitat* OR verge* OR right$-of-way* OR proximity OR contiguous OR line$) AND *Outcomes:* (dispers* OR population* OR communit* OR abundan* OR distribution$ OR “species composition*” OR attendance)
Waterways	1	*LTIs/verges:* (riparian OR riverside$ OR riverbank$ OR “river* *bank*” OR ((waterway$ OR canal$ OR channel$) AND *bank*)) AND *Outcomes:* (corridor$ OR dispersal$ OR habitat$ OR refuge$ OR abundance OR richness OR *diversity OR composition$ OR communit*)
	2	*LTIs:* (river* OR channel$ OR stream$) AND *Verges:* (riparian$ OR *bank* OR proximity OR bridge$) AND *Outcomes:* (dispers* OR communit* OR richness OR diversity OR drowning OR roosting OR “alien plant*”)

The asterisk (*) replaces any group of characters, including no character. The dollar sign ($) replaces zero or one character. The quotation marks (“”) allow to search an exact phrase. Strategies 1 and 2 are explained in Additional file 3

#### Publication databases

We first listed the databases to which the members of our review team had access. The database selection was then based on three criteria [25]:

- Topic: the database(s) had to cover ecology;
- Accessibility/reproducibility/sustainability: the database(s) had to be accessible by the whole review team, and by researchers all over the world (as a guarantee of reproducibility and further reviewing);
- Comprehensiveness: number of articles indexed in the database(s) among the articles of the test list (Additional file 2)

These criteria led us to select two databases: Web Of Science Core Collection (with subscriptions: Science Citation Index Expanded 1956–present, Social Sciences Citation Index 1975–present, Arts and Humanities Citation Index 1975–present, Conference Proceedings Citation Index-Science 1990–present, Conference Proceedings Citation Index-Social Science and Humanities 1990–present, Book Citation Index-Science 2005–present, Book Citation Index-Social Sciences and Humanities 2005–present, Emerging Sources Citation Index 2015–present, Current Chemical Reactions 1985–present, and Index Chemicus 1993–present; 86 articles indexed out of the 102 articles of the test list) and Zoological Records (subscribed timespan 1864–present, 51 articles out of the 102 articles). Searches on these two databases were made on “Topic”.

#### Search engines

We performed additional searches using three search engines:

- Google Scholar (https://scholar.google.fr/);
- BASE (Bielefeld Academic Search Engine, https:// www.base-search.net/);
- CORE (https://core.ac.uk/). Because these search engines could only handle a limited number of search terms and did not allow the use of all wildcards, the search strings used for publication databases were simplified. We thus developed a search string for each of the five LTIs (Additional file 3). In Google Scholar, results were sorted by relevance, with the boxes “include patents” and “include citations” unchecked. In BASE, results were sorted by relevance, with the box “boost open access documents” unchecked and the box “Verbatim search” checked. For each of the five search strings, we retrieved the first 20 hits.

#### Specialist websites

We searched for links or references to relevant articles and data on 11 specialist websites including a journal special issue on transportation ecology (Additional file 4).

#### Supplementary searches

To retrieve grey literature, we contacted by email national and international experts of transportation ecology, through the Ecodif (now SFEcodif), Transenviro, Wftlistserv and IENE mailing lists and by posting a call on social media (https://fr.linkedin.com/). SFEcodif is a French mailing list about ecology and evolution which counted around 7000 subscribers (https://www.sfecologie.org/sfecodif/), and Transenviro, Wftlistserv and IENE mailing lists are international mailing lists about transportation ecology. Together, the Transenviro and Wftlistserv mailing lists (http://www.itre.ncsu.edu/CTE/Lists/index.asp) gathered about 600 contacts and the IENE mailing list (http://www.iene.info/) counted around 300 contacts. All these mailing lists were accessed on 22 September 2015. Eventually, we contacted nearly two thousand people (N = 1902) by individual email. Organizations funding the systematic review also provided us with their unpublished reports. As well, some experts spontaneously sent us documents on flora after having heard about our project (see below).

#### Dates of literature searches

Literature searches were performed in three stages. First, we performed searches in Web Of Science Core Collection publication database, in Zoological Records publication database, and in Google Scholar search engine on April 27th 2015, February 1st 2016, and March 4th to 9th 2016, respectively. The call for grey literature was performed on April 21st 2015. All articles published in 2016 were not considered during these first searches.

Second, searches were updated for a first time on June 15th 2018 for Web Of Science Core Collection and Zoological Records publication databases, and on November 6th 2018 for Google Scholar, to retrieve articles published from 2016 onward until these dates (Additional file 4). New searches on specialist websites were conducted from November 26th 2018 to December 4th 2018, and searches on BASE and CORE search engines were updated on November 7th and 8th 2018, respectively.

Third, we performed a second update from articles published between 2018 and 2020 on March 3rd, 2021 for Web Of Science Core Collection (N = 6193 articles) and Zoological Records publication databases (N = 1240 articles). Due to time restriction, no update was conducted however for the other sources of literature (Google Scholar, CORE, BASE). There was no update of the call for grey literature either, but we received documents from experts by emails: on 20th October 2021 from Mayenne Nature Environnement (N = 10 articles) and on 15th November 2021 from Frédéric Hendoux, director of the Conservation Botanique National du Bassin Parisien (N = 4 articles).

Finally, we considered 50 articles included in a previously published review on roadside management [29]. Among these 50 articles, there was an overlap with our searches but for various reasons (search string, literature sources) our search strategy had not retrieved some others. We screened these articles for inclusion/exclusion into our review according to our eligibility criteria.

### Article screening and study eligibility criteria

From this point, the method described is specific to the systematic review on flora.

#### Screening process

The articles collected from online publication databases were screened by several members of the review team for eligibility (according to the criteria described in the next section) through three successive stages: first on titles (performed by SV, AV, AJ, RS, MV, EG, EM, VR, LP), second on abstracts (performed by SV, AV, AJ, RS, MV, EG, EM, VR, AC, YB, LP), and third on full-texts (performed by LP, VF, VR, SV, AV, AJ, AC, DYO, EG, EM, MV, RS, YB, YR).

The screening was conservative at each stage: in cases of doubt, articles proceeded to the next stage for further assessment. The level of agreement between screener was assessed before beginning a screening stage (title, abstract or full-text screening stage) by computing a Randolph’s Kappa coefficient [25] on a number of references randomly sampled among the database of articles about vascular plants. Note that consistency between reviewer’s decisions at full-text screening was only tested for the most recent searches update in 2020. The randomly sampled articles were screened by each of the reviewers independently of each other. We considered 200, 20 and 50 randomly sampled references to be sufficient to assess the agreement between screeners during title, abstract and full-text screening, respectively. It is a relatively small proportion of the total number of references to be screened, but these numbers were based on our experience with the previous systematic reviews published on insects and vertebrates [26, 27]. The minimal level of agreement between reviewers was set at 0.6, which was considered by CEE Guidelines as an acceptable level when our protocol was published. All disagreements were discussed by reviewers, so that differences in screeners’ understanding of eligibility criteria could be resolved. When the coefficient was lower than 0.6 the operation was repeated until reaching a coefficient larger than 0.6.

#### Eligibility criteria

At each stage of screening, article eligibility was based on a list of selection criteria. At the stage of title screening, these criteria mainly encompassed both the subject (ecology and related disciplines) and the population and exposure/intervention of the article (Table 4). The same criteria were applied at the stage of abstract screening, to which we added criteria regarding the exposure/intervention, the comparator, the outcomes or the study type (Table 5). Articles without an abstract were discarded due to their high number and time constraints, and as this protocol was followed for the previous two reviews on insects and vertebrates of our project. Finally, the same criteria as for the abstract stage were used for the stage of full-text screening, to which we added new inclusion criteria regarding the language, the climate, the type of publication or the specific questions covered (Table 6). We considered that a study was not relevant to the purpose of the review (and thus discarded it) if the comparator was inappropriate (e.g. comparison between different seasons, high contrast of habitat with the comparator such as herbaceous verges compared to forests), or if the sampling was not strictly done on the verges. As our review focused on transportation infrastructure, we also made sure at the full-text screening stage that only paved roads and navigable rivers and canals were included. This information is unfortunately rarely provided for waterways, so we included all articles with Strahler [30] stream order above 3, canals and rivers, and excluded all articles with stream order equal or below three and articles with no information on stream order. Studies on waterways dealing with our specific question Q2 on the role of habitat of LTI verges were also excluded at the full-text screening stage, based on a lack of an appropriate comparator. Indeed, the studies gathered compared vegetation communities on the riversides to those further away from the stream, or the vegetation along small streams and larger rivers. Such comparisons conflate differences reflecting distinct riparian habitats with the exposure to an LTI and therefore were judged inadequate to answer our specific question.

**Table 4:** List of inclusion/exclusion criteria at the stage of title screening

Include	Exclude
*For* *all* *LTIs*	
- Articles dealing only partially with the role of habitat or corridor of the verges - Articles regarding invasive species if the role of corridor or habitat of verges is mentioned - Articles regarding soil biodiversity - Articles dealing with the effects of chemical, noise or light pollution on verge biodiversity (even if the pollution comes from the infrastructure itself) - Articles out of the temperate climatic zone (this criteria is assessed at the full-text reading stage) - Articles regarding wildfires (they are assessed at the full-text reading stage)	- Studies regarding green infrastructures in general without considering the specific case of LTIs - Studies regarding overpasses/underpasses or fragmentation due to LTIs considered transversally, without considering the roles of habitat and corridor of verges - Studies regarding paleontology, phylogenetics, phylogeography and taxonomy (including studies describing newly discovered species) - Genetic studies without any relation to a natural habitat (in particular biodiversity meta-genomics studies) - Pedological studies without any relation to biodiversity - Studies regarding medicine, toxicology or chemical, noise or light pollution without any relation to biodiversity
*Specifically* *for* *fluvial* *LTIs* *(waterways)*	
- Articles whose title mentions the words floodplain, riparian, wetland, seasonal pond, intermittent stream or spawning (in which case the article is considered to deal with the semi-aquatic part of the river, that is to say the banks, emerged during the dry season and immersed during the wet season, which is part of the scope of the review) - Articles regarding amphibious species - Articles regarding seed dispersal through waterway flow (hydrochory) - Articles recommending management actions to perform under bridges - Articles regarding streams (they are assessed at the full-text reading stage)	- Articles regarding exclusively aquatic species, except if the title mentions the words floodplain, riparian, wetland, seasonal pond, intermittent stream or spawning (in which case the article is considered to deal with the lateral part of the river, that is to say the banks, sometimes immersed other times emerged, which is part of the scope of the review) - Articles regarding lakes and islands or sand banks in the middle of rivers - Articles regarding river debris (organic matter, tree trunks, underwater leaves decomposition, except if the article deals with the submerged part of the bank, etc.)
*Specifically* *for* *non-fluvial* *LTIs* *(roads,* *railways,* *power* *lines,* *pipelines)*	
- Articles regarding the role of verges in plant dispersal - Articles recommending verge management actions to perform	- Articles regarding plant dispersal without any relation with the role of habitat or corridor of the verges

**Table 5:** List of inclusion criteria at the stage of abstract screening

Type of criteria	Description
Relevant population(s)	All tracheophyte biodiversity (at the species, community and ecosystem level), including exotic invasive species
Types of exposure/intervention	Any article exposing biodiversity to a LTI verge (road, railway, power line or pipeline verges or waterway banks), to a LTI verge management (mowing, pesticide spreading, pruning, planting, fence laying, etc.) or to a LTI verge disturbance (chemical, air pollution, wildfires, etc.)
Types of comparator	Unexposed/intervention-free control site or before-exposure/before-intervention control site
Types of outcome	All outcomes relating to corridor and habitat assessment or effects of verge management, such as dispersal (including species invasions, hydrochory and seed dispersal by vehicles), species richness, Shannon index, Simpson index, beta diversity, community composition and abundance of different taxonomic or functional groups of organisms
Types of study	All type of studies should be included apart from modelling (theoretical) articles, articles making recommendations without making experimentation and articles making experimentations in laboratory conditions

**Table 6:** List of inclusion criteria at the stage of full-text screening

Type of criteria	Description
Language	Full text written in English or French
Climate	Articles with study zone(s) of the temperate climate
Type of publication	Articles different from editorial material, meeting abstracts, news items and review
Comparator	Articles with control/compared site
Road type	Articles with paved road (not unpaved road, path, gravel road, forest road)
Waterway type	Articles with stream order above three, canals or rivers
Specific questions	Articles that give relevant results for the six specific synthesis questions detailed in Table 1 and 2

To identify whether study area was in the temperate climate we used the Köppen–Geiger Climate Classifcation (Cfa, Cfb, Cfc, Csa, Csb, Csc, see http://people.eng.unimelb.edu.au/mpeel/koppen.html for the GoogleEarth layers of the Köppen–Geiger Climate Classification). When a study area overlapped temperate and non-temperate climate with no possibility to extract the data regarding only the temperate climate, the corresponding study was discarded. Similarly, studies were excluded if the results included biological groups and/or exposures that were not under the scope of the review, with no possibility to extract results scoping the review (e.g. results combining aquatic and riparian flora, or combining vascular flora and lichen or bryophytes, results combining paths and paved roads, results combining streams and rivers). We also checked for data redundancy (data already published in another article included in the review) and added this factor as an exclusion cause.

### Study validity assessment

We conducted a critical appraisal of the studies and assigned them a low, medium or high risk of bias. To define the criteria of this appraisal, eight external experts in landscape connectivity and transportation ecology were gathered and consulted during a 1-day workshop with seven scientists of our review team [25]. During the workshop, we discussed the gold standard protocol of an ideal study answering our primary question with unlimited resources (unlimited money, time, workforce, etc.).

We relied on scientific literature to select the most recommended criteria regarding potential biases in ecology studies [31, 32]. In particular we paid attention to:

- replication vs pseudo-replication, considering that the lack of independence between samples may introduce bias into the results [33–35]
- the type of study design (BACI, CI, BA), considering that a dual temporal and spatial comparator (BACI) should be preferred on a single one (CI or BA) [36–38]
- confounding factors defined as 'parasitic' elements that may affect the outcomes measured [39]

We considered that a study was unreliable because of a high risk of bias, and therefore excluded it from further synthesis, if there was/were:

- A total absence of replication;
- An inadequate methodology (for example for question Q4 on the role of corridor of verges, a statistical analysis of dispersal data that did not allow to distinguish LTI verges from other habitats);
- A method description strongly insufficient (i.e. when it was not possible to know where the sampling was done: within or outside LTI verges);
- Major confounding factors (e.g. strong difference in sampling effort between treatment and control).

We considered that a study had a medium risk of bias if it had the following characteristics:

- Absence of transparent and systematic procedure for the selection of sample plot location (i.e. randomization, fixed distances, grids);
- Control–Intervention and Before–After–Intervention study designs (as opposed to Before–After–Control–Intervention study designs) for the specific questions involving verge management (questions Q1 and Q3);
- Absence of true spatial replication of the study (for example study with repetition of measures on a unique site);
- Attrition bias (difference in the loss of samples between control and treatment) that may lead to incomplete outcome data [31];
- Slightly inadequate description of the method (some minor details were missing but did not challenge our understanding of the methods).

Finally, we considered that a study that did not have a high or medium risk of bias had a low risk of bias. Studies with a high risk of bias were discarded from the synthesis. In the narrative synthesis, the results of studies with a low risk of bias were first synthesized and then the consistency of the results of studies with a medium risk of bias was assessed. In the meta-analyses, the influence of the level of bias (low or medium) on effect sizes was furthermore tested. For articles dealing with more than one specific question (Tables 1 and 2), we performed critical appraisal for each question separately, that we considered being different studies. The critical appraisal was performed as follows: first, each study was critically appraised by one reviewer (VF). Then, a second reviewer critically appraised again the uncertain cases. We compared conclusions of the two reviewers, and when they differed, they discussed disagreements until reaching a consensus and asked for a third reviewer if necessary. Reviewers never critically appraised an article they had authored. Although it is a CEE standard that at least two people independently critically appraise each study, it was not possible in this study due to the high number of articles and time constraints.

### Data coding and extraction strategy

#### Extraction of meta-data

We used the coding tool displayed in Table 7 to produce an easily searchable database of the studies included after critical appraisal (i.e. with low and medium risk of bias). When an article dealt with more than one of our specific questions, it was split in as many studies, each coded in a distinct row.

**Table 7:** Coding tool for the database of included studies

Coding variable	Details/examples
ID	Unique identifier of the publication
Source	Source of the publication (e.g. WOS, ZR, grey literature)
Publication author(s)	
Publication year	
Publication title	
Publication journal	
DOI	
Publication type	Book chapter, journal article, thesis, report, etc
Article language	English/French
Specific question	Question Q1, Q2, Q3, Q4, Q5, or Q6
Study design	Spatial/Temporal/Spatial and temporal comparisons
Risk of bias	Low/Medium
Study country	
Study region(s)	
GPS coordinates	
Biological group(s)	Trees/herbs/shrubs, forbs, riparian flora, etc
LTI	Roads/Railways/Powerlines/Pipelines/Waterways
LTI verge	Description of verge and its habitat (grassland, shrubland, hedge, forest, etc.)
Comparison	- Questions Q2 and Q4: type of habitat of the control site; - Question Q1 and Q3: management practices (mowing, pesticide spreading, pruning, planting, fence laying, etc.); - Questions Q5 and Q6: landscape metric(s) and spatial scale(s)
Outcomes	Abundance, species richness, Shannon index, etc
Included in narrative synthesis	
Included in meta-analyses	

#### Extraction of data for narrative synthesis

For all specific questions except Q2 (for which data was extracted for meta-analyses only), we first extracted into tables the statistically tested results of all studies with low and medium bias. For each species or group of species we extracted the effects of exposure/intervention and for outcomes related to abundance and species richness or diversity and categorized them as positive, negative or neutral. Neutral effects referred to comparison between control and treatment that were statistically not significant (i.e. no statistically significant difference between the two, α = 0.05). Where necessary, we assessed whether the differences were statistically significant using the confidence intervals reported by the authors. We also extracted whether exposures/interventions yielded significant changes in species assemblages and community composition. For questions Q3, Q4 and Q6, data extraction was performed by one reviewer (VF) and then checked by a second reviewer (HM). Data extraction for Q1 and Q5 was done by one reviewer (HM).

#### Extraction of data for meta-analyses

For each primary study, and for both LTI verges and control sites away from LTIs, sample sizes, outcome means, and measures of variation (standard deviation, standard error, or confidence interval) were extracted from tables, text, published raw data (e.g. in appendices), and graphics using the R package “metaDigitise” version 1.0.1. [40]. When outcome means or measures of variation could not be directly extracted from the published data, the sample size and any other measure that enable further imputation according to Lajeunesse [41] (e.g. upper- and lower inter-quartile ranges, statistical tests parameters) were extracted. In cases of uncertainty regarding the measure of variation reported (i.e. when it was impossible to know whether it was the standard deviation or the standard error that was reported), standard errors were assumed to obtain conservative estimates of the uncertainty around the effect sizes calculated. Abundance for either species groups or individual species were extracted. If a study reported the abundances for both a group and some particular individual species from the group we only used the former. Similarly, if total abundance or richness were given alongside data for specific subgroups (e.g. annuals, forbs, or ruderals), only global values were used except if the grouping concerned the native or exotic/invasive status of species, in which case results were also extracted in addition to the global ones. When studies measured the biodiversity of vascular plants at various distances from LTI verges we used values at the furthest distance as controls. When studies reported multiple measures along a distance gradient within the boundaries of the LTI verges, means and standard deviations were combined and used to compute a single effect size. Finally, if a study reported several sites that could serve as a control, the site with habitat most similar to LTI verges was chosen as control. One reviewer (HM) performed data extraction for the meta-analyses and a sample of data (60%) was retrospectively cross checked by another reviewer (YR).

### Potential effect modifiers/reasons for heterogeneity

We recorded the following potential effect modifiers as stated in the protocol of the present review [25]:

- Geographic location;
- Biological groups studied;
- Site characteristics: type of LTI, type of habitat of the verge/comparator sites and level of contrast between them;
- Verge management practices (mowing, grazing, vegetation burning, pesticide use, etc.);
- Comparator type (spatial/temporal, etc.);
- Selection of sampling location (randomization, fixed distances or grids versus directed sampling). Although identified as a potential reason for heterogeneity in the review protocol, we eventually considered the absence of replicates as an important source of bias. Accordingly, those articles without replicates were discarded during critical appraisal.

### Data synthesis and presentation

#### Descriptive statistics and narrative synthesis

The meta-data extracted from each study were used to produce descriptive statistics of the evidence. Then, for all specific questions except Q2, we produced a narrative table that summarized the key results of relevant studies and we wrote a narrative synthesis. Whenever possible, we organized the findings from included studies by grouping them into categories based on risk of bias, biological group, type of LTI, and/or type of management intervention.

#### Quantitative synthesis

##### Eligibility for meta-analysis

Meta-analyses were only possible for the specific question Q2 (role of habitat of LTI verges) because only this question gathered enough homogeneous studies in terms of comparator and outcome with the required statistics.

To be included in the meta-analyses, studies had to report mean, sample size and some measure of variation for vascular plants abundance or species richness, for both LTI verges and another habitat away from the LTI that served as a control (in addition to the inclusion criteria used for the whole review). When we could not get some measure of variation from primary studies they were estimated via data imputation using the available means and standard deviations of all the studies with complete information [41].

In the meta-analyses, we used as response variables abundance (plant cover estimates) and its proxies (density of seedlings, density of stems or number of individuals), as well as species richness.

##### Meta-analyses

To assess the response of vascular plants to LTI (specific question Q2) we used the Hedges’ $d$ standardized mean difference [42] as a measure of the effect size for both abundance and species richness:

1 $${d}_{i}=\frac{{\overline{X}}_{i,treatment}-{\overline{X}}_{i,control}}{{S}_{poole{d}_{i}}}\times {J}_{i}$$

where ${\overline{X}}_{i,treatment}$ and ${\overline{X}}_{i,control}$ are the means for study of treatment sites on LTI verges and control sites away from LTI, respectively. Thus, the effect size ${d}_{i}$ is positive if the abundance or species richness is higher in LTI verges than in sites away from LTI.

${S}_{poole{d}_{i}}$ is the pooled standard deviation of the two groups:

2 $${S}_{poole{d}_{i}}=\sqrt{\frac{\left({n}_{i,treatment}-1\right)\times S{D}_{i,treatment}^{2}+\left({n}_{i,control}-1\right)\times S{D}_{i,control}^{2}}{{n}_{i,treatment}+{n}_{i,control}-2}}$$

where $S{D}_{i}$ is the standard deviation and ${n}_{i,treatment}$ and ${n}_{i,control}$ are the sample sizes of treatment and control groups.

${J}_{i}$ is a correction for small sample size:

3 $${J}_{i}=1-\frac{3}{4\times \left({n}_{i,treatment}+{n}_{i,control}-2\right)-1}$$

To calculate the variance for Hedges’ ${d}_{i}$ we did not use the standard approach with Hedges’ estimator [43] because Hamman et al. [44] demonstrated that it is biased under conditions common in ecological meta-analyses.

Instead, we used the alternative estimator proposed by Hedges [45]:

4 $$va{r}_{i}={\left(1-\frac{3}{4*\left({n}_{i,treatment}+{n}_{i,control}-2\right)-1}\right)}^{2}*\left(\frac{{n}_{i,treatment}+{n}_{i,control}-2}{\frac{{n}_{i,treatment}*{n}_{i,control}}{{n}_{i,treatment}+{n}_{i,control}}*\left({n}_{i,treatment}+{n}_{i,control}-4\right)}\right)$$

We used linear mixed-models with the restricted maximum-likelihood (REML) estimator to estimate the grand mean effect size and test the effect of moderators. Because one study could gather several cases, we nested the cases within the studies as random effects for each analysis. A case referred to an individual effect size extracted from a study (e.g. abundance of a particular species or group of species); a given study possibly gathering multiple cases (e.g. abundance of several species or group of species).

In the models each effect size was weighted based on the precision of its estimate, with more precise estimates receiving greater weights. Weights ${w}_{i}$ were computed as ${w}_{i}=\frac{1}{\left(va{r}_{i}+{\tau }^{2}\right)}$ with ${\tau }^{2}$ the among-study variance estimated during the meta-analysis.

For each response variable (i.e. abundance and species richness), we first computed the grand mean effect size combining all studies (mixed model without moderators with cases nested within studies as random effects). We analyzed the datasets for publication bias (*i.e.* when the published literature reports results that systematically differ from those of all studies conducted) using funnel plots, Pearson’s correlation coefficient between effect size and publication year, as well as a cumulative meta-analysis by publication year. Publication bias is manifested by an asymmetry in funnel plot, but other causes than publication bias can lead to funnel plot asymmetry, such as heterogeneity in effect sizes. To take this into account, we used the residuals of the model testing the effect of the type of LTI on vascular plants response to graphically examine and test for funnel plot asymmetry [46] using Egger’s regression test [47]. At this stage we also tested for the effect of the risk of bias of the studies (low or medium).

We investigated in meta-analyses the influence of LTI type and plant status (moderators) on the response of vascular plants. We evaluated their response to five LTI types: highways, non-highway roads, pipelines, powerlines, railways. We discriminated between highways from other roads because road width, verge width and disturbances (traffic, noise, light, pollution) can vary considerably between the two types.

To categorize highways, we used the description given by the authors of the publication and considered “highway”, “motorway”, “freeway”, and “6-lane roads” as highways. We evaluated the heterogeneity in vascular plants response to LTIs by assessing how it varies with plant status (native or exotic).

For testing the effects of moderators, we avoided problems associated with confounding factors by constructing independent subsets of data. We also determined the influence of individual cases on the results by computing Cook’s distance, and we removed from analysis the cases with a distance greater than one.

In all analyses, total heterogeneity ${Q}_{T}$ was partitioned into heterogeneity explained by the model (${Q}_{M}$) and heterogeneity not explained by the model (${Q}_{E}$) with ${Q}_{T}={Q}_{M}+{Q}_{E}$. The statistical significance of ${Q}_{M}$ and ${Q}_{E}$ was tested against a ${\chi }^{2}$ distribution.

All analyses were conducted in R 4.2.2 [48] using the metafor package [49] and the rma.mv() function.

## Review findings

### Review descriptive statistics

Searches about the role of linear transportation infrastructure as habitat and/or corridor for biodiversity returned 102,517 articles from 1972 to 2020 (83,565 from the initial search retrieval and 18 952 from the update in 2020). After the title screening stage, 27,649 articles were accepted. During the abstract screening stage, we rejected 23,601 articles from the corpus, yielding 4048 articles for full-text screening.

**Fig. 1:**
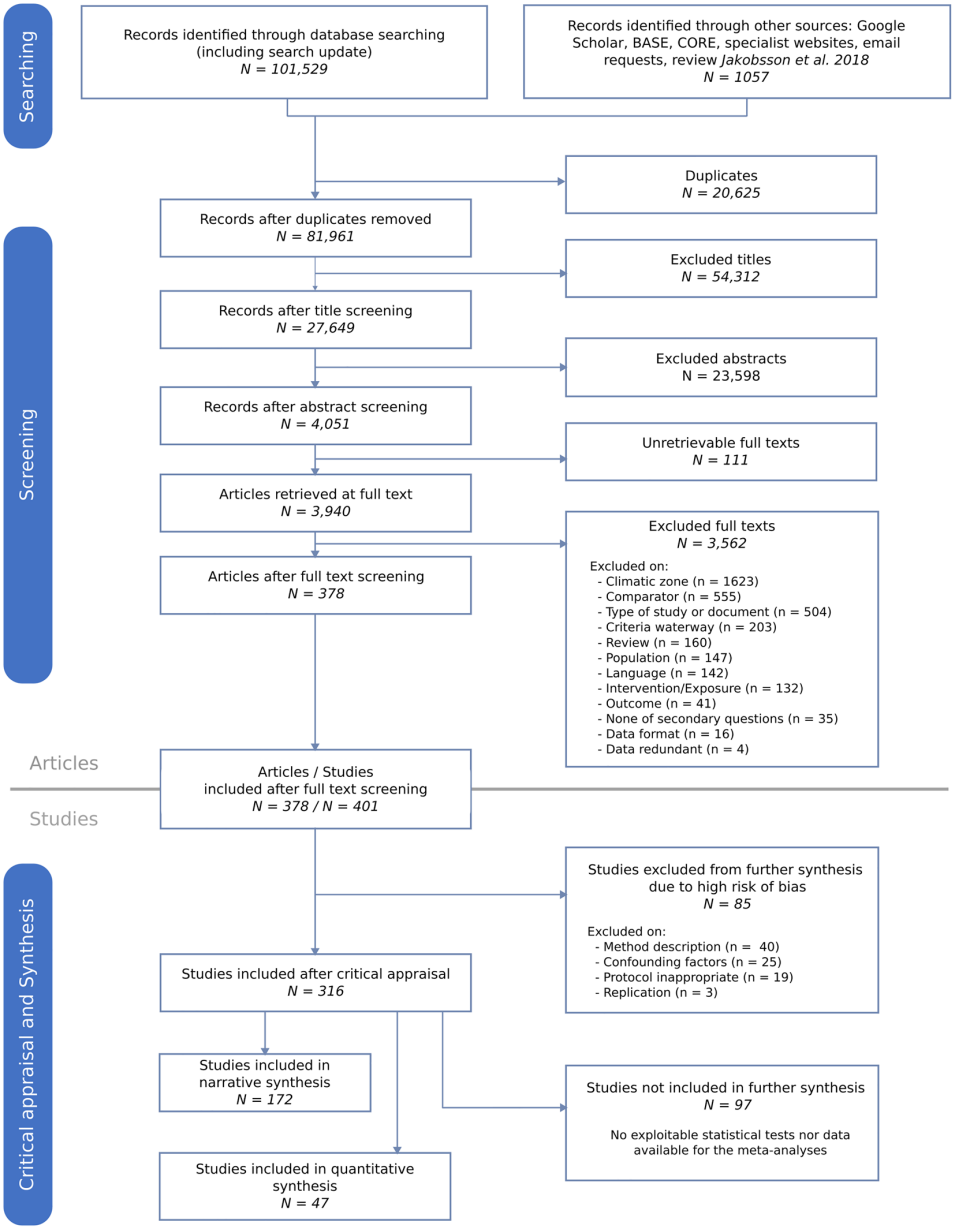
Flow diagram reporting the screening process of the articles and studies of the review

We could not retrieve full-texts for 111 articles (3%) and among the other articles, 378 were accepted for critical appraisal from the literature search retrieval (see ROSES flow diagram in Fig. 1).

For articles that were rejected at full-text screening, the most common reasons for exclusions were: sites were not in the bioclimatic region of interest (44.2%), there was no appropriate comparator (13.6%) and the type of studies were not appropriate (e.g. a simulation study; 13.3%).

A posteriori, some articles were included in the screening process. Indeed, we considered 49 articles from the review of Jakobsson et al. [29]. This recent review assessed the role of ILT infrastructure management on the flora biodiversity. As it corresponds to our question Q1, we included the articles which were not already in our corpus. One of them was lacking an abstract and was therefore excluded during abstract screening, no PDF could be obtained for 6 articles, 25 articles were excluded during the screening stage and leaving 17 articles that were included in the critical appraisal stage. The exclusions of the 25 articles was either because studies were not conducted under a temperate climate (76%), or because the document was written in a language other than English or French (24%).

Later in the search process, we also received 14 articles by mail from experts (10 from Mayenne Nature Environnement and 4 from Conservatoire Botanique National du Bassin Parisien). However, these articles were all excluded from the screening process at the full-text stage, as 4 of them were reviews and the others because of the type of study or because the exposure criteria were not met.

Thus, at the end of the screening phase, 378 articles remained (Fig. 1; see Additional file 5 sheet B for a list of all articles retained after the screening phase). The articles rejected at full-text screening as well as those for which we did not find full-texts are also listed in Additional file 5 (sheet A) with reason for exclusion.

#### Study validity assessment

Because an article can contain data relevant to more than one of our specific subquestions, the 378 articles included in the review were split into 401 different studies that underwent critical appraisal.

At this stage, 85 studies were excluded from further synthesis because they showed a high risk of bias. The reasons for a high risk of bias were a strongly insufficient description of the method (45%), the presence of a major confounding factor (28%), an inadequate methodology (19%), an absence of replication (3.5%) and protocols varying between control and treatment (2%) or between sites (1%).

**Fig. 2:**
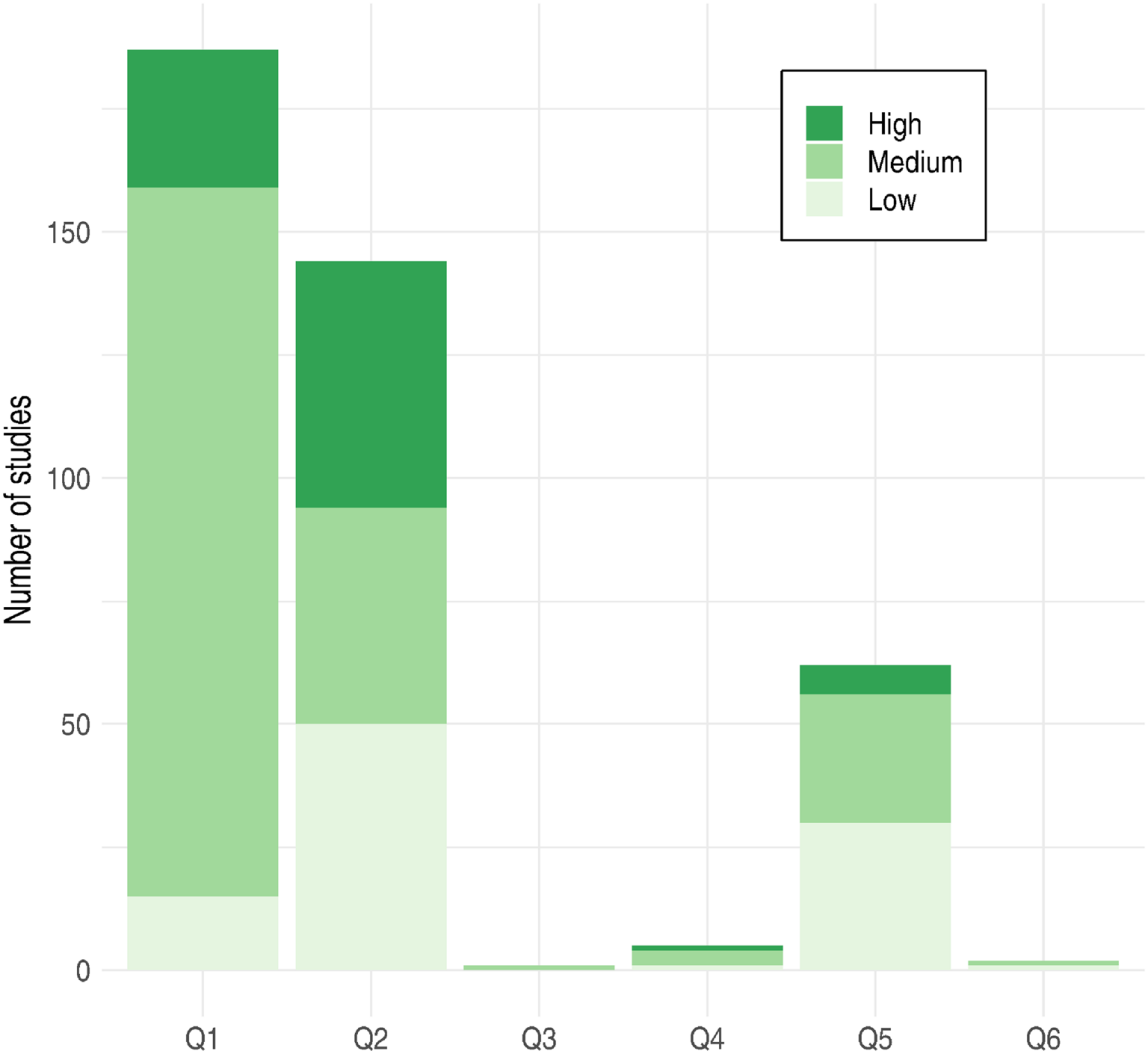
Number of studies with low, medium, high risk of bias for each specific question

Thus, a total of 316 studies corresponding to 294 articles were retained after critical appraisal (Additional file 5 sheet C). Additional file 5 (sheet D) contains also a list of all studies retained after the screening stage with coded metadata and their risk of bias assessed during critical appraisal. The number of studies of each level of bias for every subquestion and type of LTI is also given in Fig. 2.

After the critical appraisal stage, 172 studies with a low or medium risk of bias which reported statistical results for any subquestion except Q2 were included in the narrative synthesis (Table 8). The 94 studies with a low or medium risk of bias dealing with Q2 were scanned separately for means and error measures for control and treatment sites in order to compute effect sizes for a meta-analysis on the role of habitat of LTI verges. Among them, 47 studies provided the necessary data for *Species* *richness* and/or *Abundance*.

**Table 8:** Number of studies included in the narrative synthesis for each of the 6 subquestions

Specific question	Management intervention subgroup	Susceptibility to bias
Q1 habitat/management practices	Artificial fluctuation of water level due to dam regulation	2 low, 21 medium
	Channelization of rivers	2 low, 3 medium
	River restoration	10 medium
	Riverbanks engineering	10 medium
	Biomass reduction (mowing, grazing, etc.)	4 low, 41 medium
	Exotic/weed management	4 low, 26 medium
	Revegetation techniques	2 low, 21 medium
Q3 dispersal/management practices	–	1 medium
Q4 dispersal in LTI verges *vs* at proximity	–	1 low, 3 medium
Q5 habitat/surrounding landscape	–	21 low, 17 medium
Q6 dispersal/surrounding landscape	–	1 low, 1 medium

### Description of the studies

Of the 316 studies with low or medium risk of bias, 30% had a low risk of bias (Fig. 2).

The majority of studies dealt with the role of habitat of verges by comparing biodiversity within the boundaries of LTI verges to biodiversity in similar habitats away from the verges (Q2; 94 studies), and with the impact of management practices on biodiversity (managed LTI verges vs non-managed LTI verges, LTI verges under different management regimes or perturbation levels) (Q1; 159 studies). A relatively high number of studies also investigated the effect of the surrounding landscape on the habitat role of verges (Q5; 56 studies). However, studies addressing the role of verges as corridors were very scarce. Indeed, there were only 4 studies on the role of corridor of LTI verges (Q4), 1 study on the impact of management practices on the role of corridor (Q3) and 2 studies on the influence of the surrounding landscape on the role of corridor (Q6).

#### Source, language, document type

Most of the 294 articles retained after critical appraisal were retrieved from Web Of Science Core Collection (265 articles). Only 1 article came from Zoological Records and only 1 from Google Scholar. The call for grey literature eventually led to 10 articles being included in the final corpus and 2 articles were also spontaneously sent to us. Lastly, 15 articles came from the review of Jakobsson et al. [29] on the impact of management practices of road verges on biodiversity.

The vast majority of the studies included in the synthesis came from documents written in English (94%), the remainder being written in French.

Almost all of the articles included were published scientific articles (95%), but the review also included 11 technical reports, 2 Master theses and 2 PhD thesis chapters, as well as 1 Powerpoint presentation and 1 book chapter.

#### Geographical range

**Fig. 3:**
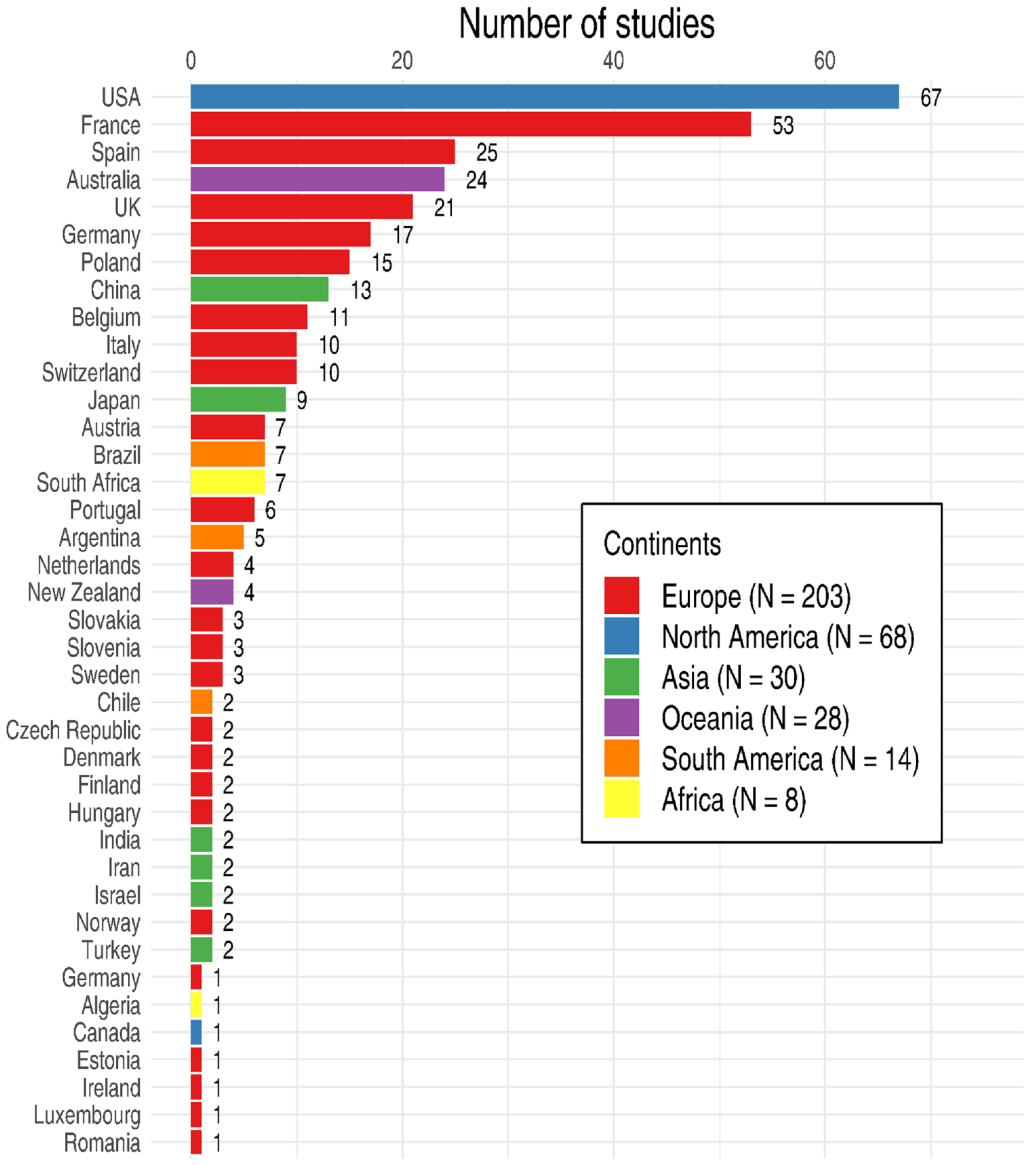
Study locations (country and continents)

At the country level, most of the 316 studies were done in the United States (21%) and France (17%), then in Spain (8%), Australia (8%) or in the United Kingdom (7%; see Fig. 3 for a full list).

#### Year of publication

**Fig. 4:**
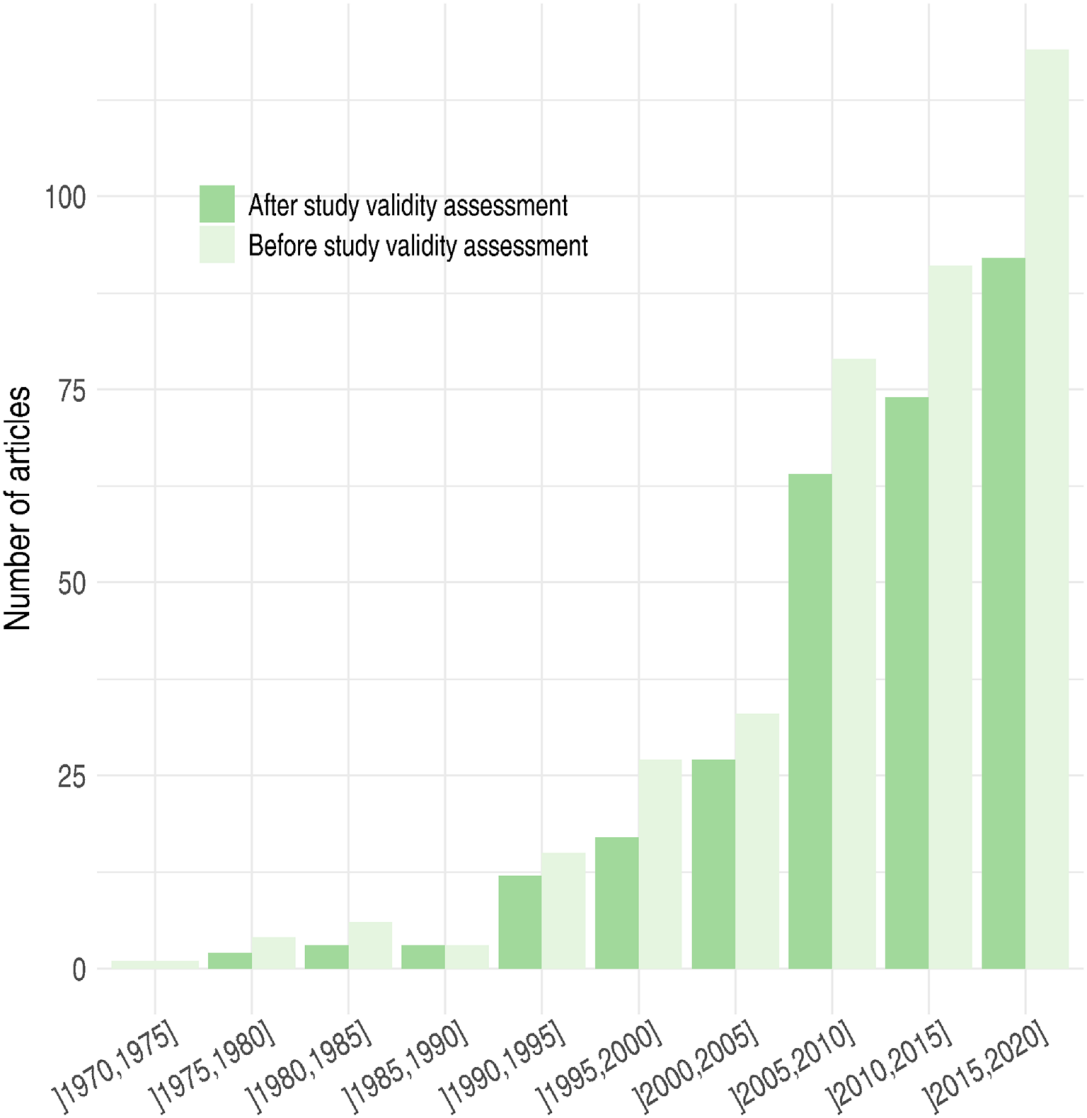
Years of publication of the articles retained before and after study validity assessment

The 294 articles retained after critical appraisal were published from 1977 to 2020, with a number of articles retained that increased rapidly over this period both before and after critical appraisal (Fig. 4).

#### Study design

Most studies included in the synthesis had a Control-Exposure (for questions Q2, Q4, Q5, Q6) or a Control-Intervention (for questions Q1) design (93% of the studies). Only 7% of the studies however included a temporal control by assessing biodiversity both before and after the intervention/exposure.

#### Exposure

The majority of the 316 studies were about roads (153). Waterways were the second most studied LTI with 133 studies. On the other hand, only 22, 10 and 6 studies were done along powerlines, railways and pipelines respectively.

### Narrative synthesis of study findings

The narrative synthesis of the secondary review questions encompassed 172 studies out of the 219 studies included in the evidence synthesis. The remaining studies were not incorporated into the narrative synthesis of study findings either because they dealt with the specific question Q2 which was treated in the quantitative synthesis, or because they did not report statistical results for comparisons between treatment and control. Immediately below we provided a narrative summary of the main patterns identified from the key results reported in Additional file 6 for each study.

*Do LTI verge management practices have positive, neutral or negative effect on vascular plants in LTI verges? (question Q1)*

A total of 127 studies provided statistical results to answer this specific question. Among them, 13 had a low risk of bias. They addressed a wide array of management practices to which vascular plants in LTI verges can be subjected to. Their key results are summarized in Additional file 6: Table S1. Given the diversity of the management measures studied, we present here only the main results and we provide an extended narrative synthesis in Additional file 7.

Twenty-three studies (2 with a low risk of bias) assessed the impact of artificial fluctuations of water levels in dam-regulated waterways (see group ‘a’ in Additional file 6: Table S1; Additional file 7 Sect. 1). Studies that investigated the effects of artificial changes to flooding exposure tended to show mostly negative impacts of artificial flooding in terms of species richness, as well as significant alterations of species assemblages. Similar results were generally reported by studies comparing regulated sections of streams to either less regulated or completely unregulated watercourses. In the case of stream regulation, the importance of exotic plants in the communities sampled generally increased with the degree of regulation.

Five studies (2 with a low risk of bias) assessed the impact of the channelization of rivers on vascular plants (see group ‘b’ in Additional file 6: Table S1; Additional file 7 Sect. 2). Results are less consistent for this small group of heterogeneous studies except for the fact that river channelization seems to favor exotic species, at least for specific sections of the riverbank.

Conversely, ten studies (all with a medium risk of bias) assessed the effects of river restoration projects of channelized streams (e.g. stream channel widening, re-meandering, reconnection of side channels, bank restoration etc.) (see group ‘c’ in Additional file 6: Table S1; Additional file 7 Sect. 3). Two studies compared vegetation communities before and after the implementation of restoration measures. Changes in plant cover and species richness were reported but they were sensitive to the type of vegetation considered and/or only transient. The remaining studies compared riparian vegetation in restored sections to either unrestored, ‘near-natural’ reference sections, or areas restored using a distinct restoration measure. Results for species richness and diversity indices were rather inconsistent between studies or even between sites within a given study, which may reflect the heterogeneity in restoration measures.

Ten studies (all with a medium risk of bias) assessed the effects of various bank engineering techniques to protect against erosion and stabilize streambanks (e.g. groynes, riprap, willow fascines, mixed techniques, etc.) (see group ‘d’ in Additional file 6: Table S1; Additional file 7 Sect. 4). Overall, either mixed techniques or pure bioengineering seemed to yield the best outcomes in terms of species richness when compared to riverbanks with only civil engineering techniques (usually riprap protection).

Forty-five studies (4 with a low risk of bias) assessed the impact of generic vegetative biomass reduction techniques (mowing, slashing, grazing or burning) (see group ‘e’ in Additional file 6: Table S1; Additional file 7 Sect. 5). Grazing was sometimes found to be associated with higher species richness, but the pattern was not very robust across sites and studies. Clear conclusions could not be drawn for mowing studies neither because of the high variability in mowing regimes applied (timing, frequency, mowing height, combined with herbicide treatment, etc.), which limits our ability to compare treatments across studies.

Thirty studies (4 with a low risk of bias) assessed the effects of management practices aimed at controlling populations of specific weeds and/or exotic and invasive species (see group ‘f’ in Additional file 6: Table S1; Additional file 7 Sect. 6). Specific treatments varied widely between experiments in terms of the exact cocktails of chemical substances or the removal protocol applied. Nonetheless, they reported systematically at least some success in containing the development of weeds and invasives, although some situations required to maintain a dedicated long term management program to avoid resurgences. In some cases, exotic and invasive control programs were shown to have positive repercussions on native plant communities.

Twenty-three studies (2 with a low risk of bias) assessed the effects of various revegetation techniques of LTI verges (e.g. hydroseeding, soil amendments, fertilization, planting of native cuttings, etc.; group ‘g’ in Additional file 6: Table S1; Additional file 7 Sect. 7).

Overall, seeding of new vegetation significantly improved vegetation cover on LTI verges in most experiments, with variation in the extent of the improvement often found based on the type of seed mixture (commercial seeds or seeds collected on sites, with or without native species), the use of additional treatments (fertilization, irrigation) and their interactions.

Lastly, a group of seven studies (1 with a low risk of bias) concerned more particular management techniques not related to the previous categories (see group ‘h’ in Additional file 6: Table S1; Additional file 7 Sect. 8).

*Do LTI verge management practices increase, decrease, or have no effect on vascular plant dispersal? (question Q3)*

A single study with a medium risk of bias addressed the impact of human intervention on the corridor function of LTI verges. Werth et al*.* [50] compared gene exchanges between populations of the endangered riparian shrub *Myricaria* *germanica* that were either connected or separated by a barrier (a canyon, a channelized segment or an impoundment). They found that genetic differentiation was higher between populations isolated by impoundments (or canyons), indicating that such structures can disrupt gene flows of riparian species. Average FST values however were similar between connected and isolated populations when the barrier was a channelized river segment.

*Is vascular plant dispersal on LTI verges equal to, higher, or lower than in habitats away from the LTIs? (question Q4)*

Only 4 studies provided evidence on the corridor function of LTI verges, all concerning waterways. One study by Leyer [51] with a low risk of bias measured dispersal patterns along a gradient of declining connectivity between sites and how they affected seedling numbers and species richness. They found a significant effect of connectivity on both outcomes, with decreasing trends overall as water bodies were more isolated from the main river. The remaining three studies all had a medium risk of bias and assessed whether the distance to the river had an impact on genetic differentiation [52–54]. Relationships between genetic diversity and distance to the stream were not significant for all three studies, suggesting that populations closer to waterways did not receive more alleles.

*Is habitat function of LTI verges for vascular plants influenced by the surrounding landscape? (question Q5)*

A total of 38 studies provided statistical results to evaluate the influence of the surrounding landscape on plant communities of LTI verges. The majority of them focused on the extent to which land-use classes related to anthropogenic pressures might constrain levels of diversity within LTI verges. Hence, the degree of urbanization and the proportion of agricultural fields in the surrounding of LTI verges were most commonly assessed. Overall, mostly negative influences on plant biodiversity were associated with these land-use categories. Total species richness or percentage cover of the habitat however were not always diminished since a greater exposure to such human disturbances often resulted in a greater presence of exotic plants (e.g. [55]). Consequences on the native flora on the other hand were mostly deleterious (e.g. [56]). Nevertheless, we can note that from studies that used distances to urban centers as a proxy to capture the effect of urbanization yield less consistent results. Conversely, the proportion of natural or semi-natural patches of woodlands and grasslands tended to be associated with richer communities (e.g. [57]).

*Is vascular plant dispersal on LTI verges dependent on the surrounding landscape? (question Q6)*

Two studies focused on the influence of the type of landscape surrounding verges on the dispersal of vascular plants. The first study with a low risk of bias by Schwoertzig et al*.* [58] compared seed dispersal along river segments in urban, suburban or peri-urban landscapes. They sampled sites on two distinct rivers and found in both cases a higher number of seeds in traps in urban rather than suburban sites. Furthermore, more than three times the number of seeds were collected in the peri-urban context than in the urban landscape, but only for one of the main rivers. The second study by Yager et al*.* [59] had a medium risk of bias and investigated whether woody shrubs could act as barriers against the invasion of the cogongrass, *Imperata* *cylindrica*. They found that mean dispersal distance did not differ between roadsides next to pine-tallgrass forest or pine-shrub forest. Yet, the mean maximum dispersal distance and the number of spikelets that dispersed farther than 5 m were greater into the pine-tallgrass forest than in the pine-shrub forest.

### Quantitative syntheses

#### Description of the study cases

We extracted quantitative data from 47 studies among the 97 with a low or medium risk of bias addressing the specific question Q2 (“Is tracheophyte biodiversity in LTI verges equal to, higher, or lower than in similar habitats away from the LTIs?”). Among the 205 cases extracted from these 47 studies, 109 cases in 24 studies concerned species or group abundance, and 96 cases in 34 studies concerned species richness (Additional file 8).

We estimated the variance of cases with data imputation (*i.e.* filling missing variance by using the available means and standard deviations from the other studies as described in [32]) for 24 cases (15.6% of *Abundance* cases and 7.3% of *Species* *richness* cases). When several cases were extracted from the same study, this was because there was data for several species or group of species, for several sites, or for several years or seasons.

Most of the cases in our data were conducted along roads (62%, including 23% of highways), then along powerlines (20%), pipelines (17%) and railways (1%).

Cases extracted for the meta-analysis were mostly from studies conducted in North America and Europe (North America: 41%, Europe: 39%, Oceania: 11%, South America: 5%, and Asia: 5%).

#### Mean effects and publication bias

For abundance, overall we found that vascular plants were marginally less abundant on LTI compared to similar habitats away from verges (grand mean effect size d = − 0.79, 95% confidence interval [− 1.57, − 0.02]). However, a leave-one-out analysis revealed that this negative relationship was mainly driven by outlier cases from one study (Additional file 11). A similar diagnostic was made using Cook's distances, with two cases having values above the 0.5 threshold suggestive of influential data points, both extracted from this same study (cases 2 and 6 from Eyitayo et al. 2020; Additional file 8). With these two influencial cases removed, the overall grand mean effect size became positive but was not statistically different from zero (d = 0.18, 95% CI [− 0.56,0.92]). Based on this more robust estimate, abundance on the verges did not seem to differ from the one observed in reference sites. We also found high heterogeneity between effect sizes (Qt = 3609, p < 0.0001, N = 104), indicating that moderators could explain variations in effect sizes.

For species richness, the overall mean effect size was not statistically different from 0 (d = 0.1, 95% CI [− 0.42,0.62]), meaning that LTI verges and reference sites also exhibited similar numbers of species overall. Again, we found a statistically significant heterogeneity in the effect sizes (Qt = 1503, p < 0.0001, N = 78).

There was no evidence of publication bias neither for abundance nor for species richness from the funnel plots and the plot of the cumulative meta-analysis by publication year (Additional file 9). Egger’s regression test for funnel plot asymmetry indicated that there was no statistically significant asymmetry neither for abundance (p = 0.44) nor species richness (p = 0.72).

Similarly, the year of publication was not significantly correlated with effect sizes for abundance (r = 0, p = 0.9) or species richness (r = 0.01, p = 0.91). Finally, we did not detect any influence of the risk of bias of the study (low or medium) on effect sizes (after removing the effect of type of LTI) for none of the outcome category.

#### Effects of moderators on abundance (Fig. 5a, b)

**Fig. 5:**
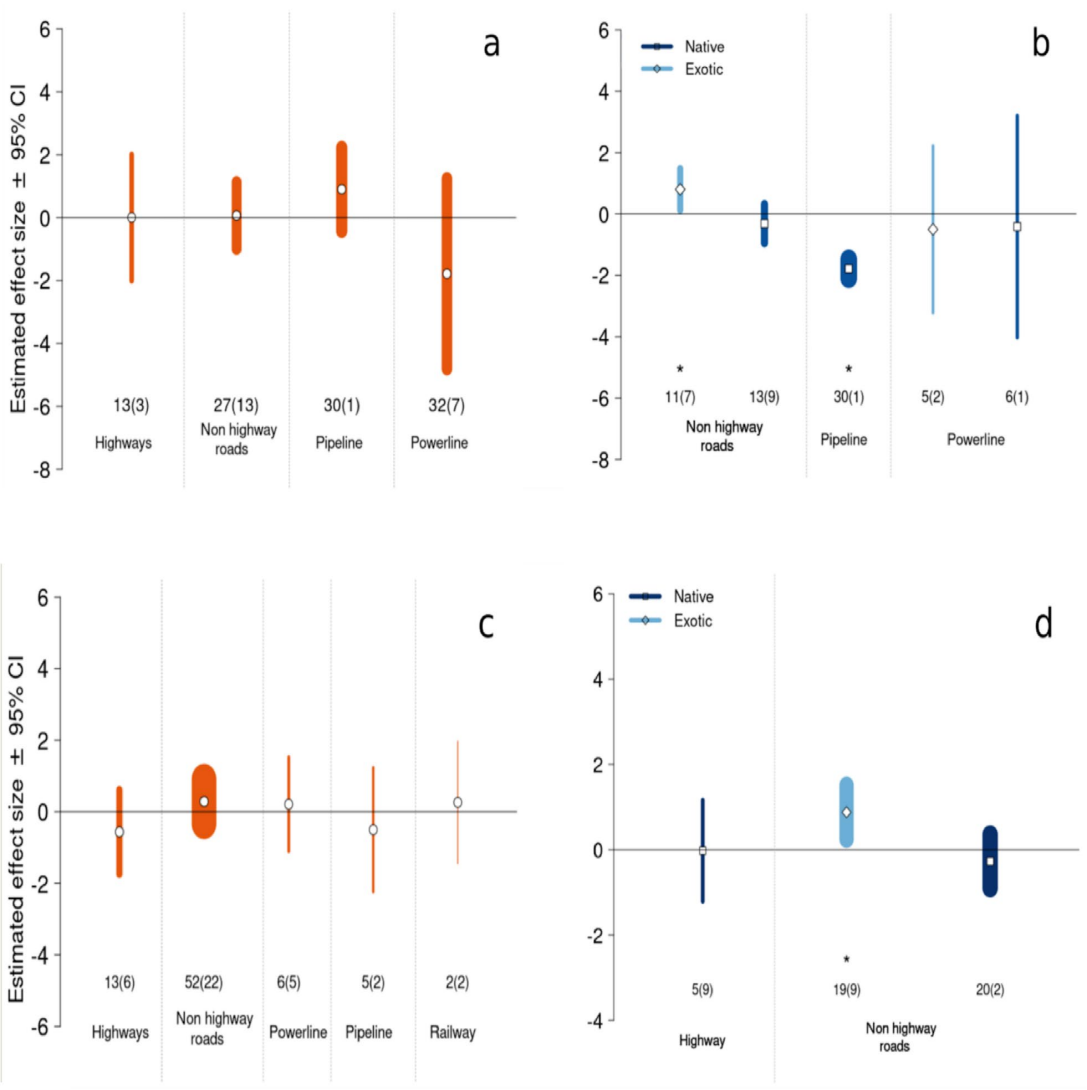
Mean effect sizes by LTI category with 95% confidence intervals. A positive effect size indicates that abundance or species richness respectively were higher in verges than in control sites. Effect sizes are calculated using ‘non-redundant’ cases if outcomes were reported for both a group of plants and a subset of that group (e.g. number of forb species and number of exotic forbs in the same study). a Abundance for all species together. Values in orange correspond to the estimates for all cases independently of plant status (native/exotic). b Abundance for native/exotic species. Values in blue were obtained from cases for either native species (dark blue) or exotic species (light blue) only. c Species richness for all species together. d Species richness for native/exotic species only. For all plots, the width of the bar is proportional to the number of corresponding cases, also indicated below each effect size with the associated number of individual studies in brackets. Estimated means statistically significantly different from zero (P-value < 0.05) are indicated by an asterisk (*)

Though no significant differences in abundance between LTI verges and sites away from the verges were found globally, we further investigated whether mean effect sizes varied by category of LTI. We first used all non-redundant cases (i.e. global values when available or values for native/exotic species only otherwise) to test the influence of the type of LTI (Fig. 5a). As only a single case was available for railways, therefore this category of LTI was discarded from the analysis. For the remaining four categories of LTIs with multiple cases, no significant differences were found between LTI verges and control sites.

Because this first analysis mixed abundance for both native and exotic species, we then looked at the differences in mean effect sizes between these two categories of flora status separately, when enough cases were available for a given type of LTI (Fig. 5b). For native species, we found that abundance was lower above pipelines than in control plots further away. However, all cases came from repeated measurements from a single study and therefore cannot be generalized. For non-highway roads, significant differences were found depending on flora status (Qm = 9.39, p = 0.01, N = 24). A greater abundance of exotic seems to occur along non-highway road verges compared to control plots (d = 0.8, 95% CI [0.13,1.48]), while no differences were found for native species. For powerlines on the other hand, no difference was found regardless of flora status.

#### Effects of moderators on species richness (Fig. 5c, d)

The influence of the type of LTI was also investigated for species richness (Fig. 5c, d). Considering all species together, we again found no significant differences between LTI categories (Fig. 5c). However, when we considered native and exotic species independently, significant differences were found again for non-highway roads (Qm = 21.06, p =  < 0.0001, N = 39), as for abundance. More exotic species were present in the verges compared to the control plots (d = 0.88, 95% CI [0.25,1.51]), while no significant differences were found for native species (Fig. 5d). Available cases for native plants along highways on the other hand did not show differences between the verges and control sites.

### Reasons for heterogeneity

For a given question, studies included in the synthesis relied on a wide variety of comparators. For instance, adjacent woodlands along a forest road can be under distinct management regimes or be dominated by very different tree species. Similarly, herbaceous roadsides were compared in some studies to peri-urban grasslands, which can be oldfields or abandoned industrial spaces, and in others to dry calcareous grasslands of high conservation value. When looking at the effects of specific management interventions, the control can sometimes be a reference plot that has been unmanaged for a given period of time (e.g. time since grazing exclusion for control pastures, time since last clear cut for control powerline corridors) and this duration is not fixed across studies. Therefore, the level of contrast between exposed or treated sites and comparators could be one of the main sources of heterogeneity.

The impacts of a wide array of practices were also documented regarding the management of LTI verges. Although we attempted to synthesize results using categories based on LTI and the type of intervention affecting the verges, important differences still remain between studies within a given category. As mentioned earlier, studies that investigate the impact of mowing can manipulate one or several parameters including the frequency and height of mowing, the timing of cuts and how they relate to the phenology of certain species, or whether the plant material is removed from the site after mowing or not. For grazing, the type of livestock and the intensity of grazing are two important parameters that vary between studies. Furthermore, different combinations of chemical products and various dosages were applied to control invasive and weeds development on LTI verges. These examples illustrate the range of differences in the implementation of management practices that may be expected to yield significant heterogeneity within a given category.

Other aspects of the designs of included studies could also explain part of the heterogeneity. In particular, the sampling periods (i.e. during which seasons) and whether vegetation surveys spanned across multiple years can influence the final results of a study. The specific survey method used, as well as the areas of sample sites are another example of important protocol choices that can affect the outcomes measured. In addition, for studies that assessed the influence of the surrounding landscape, the proxy and the scales at which land-use types were aggregated varied also greatly between studies.

The LTI and their verges themselves differ in important aspects that can be expected to influence the effects measured. Indeed, they will often show significant differences in terms of width, topology, history of management, use, etc. Such variations can have lasting impacts on the types of plant communities they can potentially host. For instance, some studies contrast the vegetation found on roadfills and roadcuts since they involve different construction processes.

Another reason for heterogeneity arises from the fact that the synthesis included all vascular plants in a wide variety of habitat settings (e.g. broad-leaved forests, steppe grasslands, river floodplains). Then, depending on whether a habitat is open or closed, soil conditions, and the history of site management, different assemblages of plant species were sampled. Consequently, these plant communities differ in terms of dominant strata (herbs, shrubs or trees), life cycles, exotic or native status, functional traits and tolerance to specific environmental parameters (nitrogen availability, moisture, etc.) of the various species, which will usually mediate to some extent how they respond to the presence of an LTI or to a particular management practice. Indeed, in the meta-analyses for the role of habitat of non-highway road verges we found that plant status explained a significant part of the heterogeneity as different patterns were obtained for native and exotic species.

### Review limitations

#### Limitation due to the methodology of the review

First, we are aware that our systematic review protocol, published in 2016, does not strictly comply with the current guidelines of CEE that have evolved since then. However, since we had already completed much of the searching and screening at this early stage we did not have the resources to go back and start over again.

Second, mostly due to logistical constraints several decisions were made during the review process that may have introduced some biases in the final output. Indeed, in order to save time during screening, articles for which abstracts were missing were excluded from the corpus (Additional file 10). Yet, CEE guidelines recommend to process articles with missing abstracts at the full-text assessment stage directly. Therefore, it is possible that the exclusion of this group of articles biased the screening process to some extent. While we acknowledge this risk, it should be noted that the vast majority of articles underwent a proper three stage screening process, as the articles missing an abstract represented less than 3% of the total of articles analyzed at the full-text stage. Furthermore, time limitations and logistical considerations due to the number of screeners involved in the review and the high volumes of articles to process prevented us from checking the consistency of screeners’ decisions at full-text screening except for the last update in 2020. Hence, this increases the probability that an observer effect introduced bias at the full-text stage because screeners may have applied inclusion criteria inconsistently from one another or over time. Nevertheless, clarifications and adjustments between screeners were made before the full-text assessment in order to limit such judgment disparities. A source of bias that can be clearly identified arises from the fact that we included only articles written in English or French. Our corpus is therefore biased towards English or French speaking countries. This language criteria notably prevented us from including in the review six articles from the synthesis by Jakobsson et al*.* [29] on management practices for roadside vegetation. However, the full list of articles excluded based on language can be found in Additional file 5. The first update relied instead on searches on specialist websites and on BASE and CORE search engines. For the second update, no search for grey literature was actually conducted (although a few documents including relevant grey literature were sent to us). Indeed, the call for literature made in 2015 was very time-consuming for few included studies; for this reason, it was excluded from our search strategy when updating the searches.

Third, we noticed a rapid increase in literature about our primary review question (Fig. 3). The content of Web Of Science also increased over time. This implies that this systematic review on vascular plants should be regularly updated. Indeed, our review was highly skewed towards roads and waterways and our results for other LTIs need to be extended by the inclusion of future studies. In addition, future updates might allow us to assess whether the differential response of exotic and native plants is robust across LTI categories. As the review focused on plants, we would also like to emphasize that it provides only a partial estimate of the potential of LTI verges as habitat and/or corridor for biodiversity (but see Villemey et al*.* [26] for insects and Ouédraogo et al*.* [27] for vertebrates).

#### Limitation of primary research

First, we observed that some information was lacking in primary studies regarding the design of the studies. One of the main difficulty concerned the comparator. Indeed, differences in substrate conditions, history of management, or level of degradation or perturbation will usually produce markedly distinct vegetation assemblages. Hence properly matching habitats along these multiple dimensions may prove to be particularly hard. Moreover, we seldom found articles that clearly described these parameters both for the LTI verges and the control plots at the time of the experiment and in the past. As a result, it is often unclear whether sufficient care has been taken to ensure that the biodiversity on verges is assessed against a comparator of good quality and not a degraded one. In the latter case, the risk especially for question Q2 on the role of habitat is to grossly overestimate the potential of LTI verges by using habitats of poor quality as a reference. Another limitation may come from the duration of studies and the scheduling of sampling throughout the year. Seasonal or inter-annual variations can produce important fluctuations in the attributes of plant communities. Furthermore, the effects of a particular intervention on verges (e.g. the clearing of alien trees) or on the LTI directly (e.g. the removal of flood defences or of a river dam) may unfold over many years. However really long-term studies were quite rare in contrast to single year studies, suggesting that the time window may not always be adequate to capture delayed or successive effects. In addition, one important shortcoming of the large majority of studies is that verges and comparators were not sampled before the intervention. This implies that variations measured between control and managed sites may actually reflect pre-existing differences instead of a real impact of management. Indeed, a simulation study by Christie et al*.* [37] demonstrated that designs other than Before–After–Control–Impact may lead to biased estimates of the true effect of an environmental impact, even when the sample size is large. Moreover, besides the limitations of the individual studies included in this review, we note a lack of common research protocol for each specific question. Indeed, researchers measured several aspects of plant biodiversity in dissimilar ways, which challenges the comparison of results among studies.

Second, we aimed to assess the potential for biodiversity of linear transport infrastructure but in the case of waterways, it was difficult to know whether they are navigable and even more difficult to know whether they are navigated: not every watercourse is a linear transport infrastructure. Indeed, the navigability of the waterways is rarely provided by authors and there is no international database that references this information. Hence, we resorted to use Strahler stream order [30] as a proxy for navigability, assuming that streams with an order above 3 might be navigable. Nonetheless, we are aware that this solution is not perfect: some articles about navigable waterways could have been excluded and others about non-navigable waterways could have been included. Moreover, studies on waterways are also particular because the distinction between waterway verges and the surrounding habitat can get blurred, especially when important fluctuations of the water level occur throughout the seasons. This also illustrates why we were not able to evaluate the role of habitat of waterway verges (Q2). Natural gradients may exist with distance from the streambed but verges are often not clearly separated from the habitat beyond the verge; then interpretations of differences in abundance and diversity along the gradient with regard to our specific question Q2 seems unwarranted.

Third, we restricted the narrative synthesis to include only those studies that involved comparisons that were statistically-tested. As a result, some of them were excluded from the synthesis, although they are still listed in Additional file 5 (sheet B/D). Part of them were purely phytosociological accounts of the flora in verges compared to reference sites. Others used stepwise regressions or model selection procedures to select a subset of best predictors. Hence, in some cases they did not report the estimates for the exposure to the LTI or for the management practices under investigation. Yet, because of the potential collinearity between metrics, the fact that a predictor was not retained in the best model does not guarantee that it has no effect. Likewise, some papers compared multiple categories including LTI verges and potential comparators but the authors do not provide results for each comparison (e.g. Kruskal–Wallis test or ANOVA without post hoc tests) such that we could not extract the effects of interest. Lastly, a proper assessment of all the evidence on changes in species and guild composition as a response to exposure to an LTI or to a specific intervention on verges was beyond the scope the review. Yet, numerous studies documented such effects, providing a richer account of the potential of LTI verges for plant communities. Further integration of these detailed impacts on species assemblage might therefore be particularly illuminating in the case of vascular plants. One caveat however is that such analyses usually yield results that are quite context-dependent and therefore more difficult to generalize.

Fourth, due to a lack of comparable studies across exposures and interventions, we could not implement meta-analyses for five out of our six specific questions. Indeed, although a high number of studies were collected on the impact of various management practices, once grouped by the type of intervention and outcome, the number of homogeneous cases were insufficient. Thus, most of the evidence was reviewed using narrative syntheses only. This method however has inherent limitations with regard to its capacity to provide reliable information on the magnitude and consistency of effects. In addition, in situations where sample size and the magnitude of underlying effects are low while variability is high, individual studies will most likely be unable to detect a real response. Such limitations in the ability to detect a real effect makes narrative syntheses more prone to negative bias [60]. Consequently, the conclusions we provided for these questions should be taken with caution, and we recommend more research on these topics to allow meta-analyses in the future. Likewise, the relatively limited number of cases available for both abundance and richness for most LTI categories, together with a recurrent lack of detailed description of the LTI verges and comparators in the publications prevented us from properly exploring the role of the sources of heterogeneity listed in the previous section.

### Review conclusions

#### Implications for policy/management

A substantial number of studies were collected on the impact of management interventions as well as the habitat role per se of transportation infrastructure verges for vascular plants. However, the high heterogeneity of the evidence base implies that comparable effects are usually reported only by a handful of studies. Furthermore, there is a geographical skew of the retained studies towards North America and Europe. Together, this limits our ability to draw clear and generalizable conclusions from our findings. Therefore, we cannot provide detailed recommendations to inform policy or management of verges. Still, a few valuable insights may be highlighted from the evidence synthesis.

First, we have seen that perturbations associated with human activities on transport infrastructure verges do not systematically result in a loss of richness or abundance of vascular plants, but usually alter significantly the composition of plant communities. This often translates into an increase in the presence of exotic species, sometimes to the detriment of the native flora. Depending on the specific context, various measures may however be taken to alter such dynamics. For instance, when vegetative biomass has to be reduced on verges (e.g. for safety reasons), removing cuttings after mowing/slashing, or planning mowing schedules that negatively impact on the reproductive cycles of problematic invasive species, can have positive impacts on plant communities. Bank engineering structures built to limit the erosion of waterway verges seem to be less harmful to plant biodiversity when they use natural biomaterials (e.g. willow fascines) in place of or in addition to more traditional civil engineering techniques (e.g. ripraps).

Furthermore, restoration measures (e.g. river dechannelization, hydroseeding of native species, alien removal in highly invaded sites) in some situations have proven to be effective at allowing plant communities to recover that are at least partly analogous to those found in less-disturbed natural sites. However, the success of the restoration plan often depended on sustained efforts over several years, therefore adequate resources must be allocated and sustained over sufficiently long time periods, if long-term results are to be achieved.

A careful assessment of the state of the verges before restoration (e.g. mapping the distribution of invasive species in the area) might also be required in some situations, as it will determine whether additional measures are necessary. As an example, dechannelization can be followed by the colonization of restored spaces by invasive species, which calls for the use of specific treatments (such as herbicide applications) to prevent the spread of the exotic flora [61]. Similarly, the long-term benefits of the removal of invasive alien species may be obtained only when coupled with targeted revegetation programs that create the necessary conditions for the reestablishment of native communities. For instance, after clearing of invasive alien woody trees, active revegetation using common riparian scrub trees (i.e. small trees adapted to open habitats) can accelerate the evolution of these cleared riparian corridors by making them less favourable to the invasion of exotic species [62]. The particular method used for alien control or biomass reduction can also be of importance. In some cases, slashing and removing cuttings from site might yield better outcomes in terms of recolonization by native species compared to other methods like burning [63]. In contrast, in other contexts where burning occurs naturally and frequently, it may be preferred to cutting as it will generate favourable conditions for the native flora.

Finally, a subset of studies underlined the importance of the surrounding landscape on the biodiversity hosted on verges. Hence, the landscape context around transportation infrastructure ought to be taken into account by managers to target the most interesting sites for biodiversity conservation. It should also be noted that measures implemented on verges to shape plant communities are expected to further impact other taxonomic groups to whom they provide food resources or habitat. Thus, an integrative approach has eventually to be adopted to assess the relative merits and select between different management practices.

#### Implications for research

First and foremost, the evidence synthesis highlights an important knowledge gap on the corridor function of transportation infrastructure verges. Given the potential of LTI verges as edges of ecological networks and given that alien species have recurrently been found to occur significantly more along verges, the lack of empirical studies assessing dispersal patterns within and outside LTI verges is problematic. This is all the more so in a context of rapid global warming where populations range boundaries will most likely shift to track changes in conditions and new invasion situations are likely to arise. It should be noted that the same observation of a lack of research on the corridor function of verges was made in the previous systematic reviews on insects [26] and vertebrates [27]. Thus, we recommend that resources be dedicated to document existing dispersal patterns along transportation infrastructure verges in various landscape contexts and to monitor future changes. Additional research on the verges of railways, pipelines and powerlines could also be prioritized as they are greatly understudied compared to roadsides and waterway banks.

Furthermore, we recommend that future investigations of the impact of management practices focus on a subset of well defined interventions implemented using standardized protocols. This would eventually allow meaningful quantitative comparisons between studies in order to better inform the management of verges, since the evidence as of today is actually quite sparse once we want to compare relatively homogeneous interventions.

To evaluate more accurately the potential of LTI verges for biodiversity conservation, it is also essential that researchers provide detailed information on the quality of the control sites used as comparators. Indeed, the fact that the biodiversity on LTI verges is similar to the adjacent habitat should be interpreted differently if the latter is a well-preserved dry calcareous grassland or a degraded meadow. Unfortunately, precise descriptions of the study sites that could be used to consistently assess the quality of the comparator were too rarely reported in the publications.

Finally, we also advise that authors not only report values for all vascular plants but also provide separate results for the native and exotic flora, as contrasting patterns are often obtained. In addition, the number of observations for the different groups as well as the means and measures of variance (with the type of measure used; e.g. standard deviation) should be reported anytime it is appropriate in the main text or in supplementary materials made publicly available. Such best practices would further facilitate the use of data in meta-analyses, thus increasing sample sizes and limiting the use of approximations to compensate for missing or unclear data (Additional file 11).

## Supplementary Information

**Additional file 1.** ROSES systematic review checklist. ROSES form for systematic review version 1.0.

**Additional file 2.** Test list. List of the 102 articles used to assess the comprehensiveness of the search with information on the method to get them and on their indexation in publication databases and retrieval with the search string.

**Additional file 3.** Search strings used for search engines. Search strings that were used for search engines Google Scholar, BASE, and CORE.

**Additional file 4.** Searches for literature summary. Summary of all the searches for literature with dates of search and number of articles found.

**Additional file 5.** List of articles at different stages of the review process. A. List of articles excluded at full-text screening with their reasons for exclusion. B. Articles retained after full-text screening. C. Studies included in the review with study validity assessment. D. Studies included in the review with coded metadata.

**Additional file 6.** Narrative tables. Tables summarizing key results of the studies included in the narrative syntheses.

**Additional file 7.** Extended narrative summary for the subquestion Q1 on the impact of management practices on the habitat role of transport infrastructure verges.

**Additional file 8.** Meta-analyses database. Table of all the variables extracted for computing effect sizes and analyzing reasons for heterogeneity in effect sizes, as well as value and variance of the effect sizes computed.

**Additional file 9.** Graphical analysis of publication bias with funnel plots and cumulative meta-analysis.

**Additional file 10.** List of excluded without-abstract articles. List of the articles that were excluded because they did not have an abstract.

**Additional file 11.** Leave-one-out analyses and output from meta-regressions models.

## Data Availability

All data generated or analysed during this study are included in this published article and its supplementary information files.
