# Supplementary material for: Can linear transportation infrastructure verges constitute a habitat and/or a corridor for vascular plants in temperate ecosystems? A systematic review
Source: Environ Evid. 2024 Mar 16;13:4. doi: 10.1186/s13750-024-00328-3 (PMC11376103; doi:10.1186/s13750-024-00328-3)
Supplement: Supplementary file 2 — Additional file 2. Test list. List of the 102 articles used to assess the comprehensiveness of the search with information on the method to get them and on their indexation in publication databases and retrieval with the search string. [file 13750_2024_328_MOESM2_ESM.docx]

**Additional File 2: Test list.** List of the 102 articles used to assess the comprehensiveness of the search with information on the method to get them and on their indexation in publication databases and retrieval with the search string.

To constitute the test list, we first requested experts to send us the five scientific articles that they considered the most relevant regarding our research topic. E-mail addresses of experts were obtained through mailing lists in ecology (SFEcodiff, Transenviro, Wftlistserv and IENE) and directories owned by members of our research team. The directories contained the contact details of 1902 persons working on green infrastructures, including scientists, LTI managers and government officers. Most of these persons were working in France but the directories also included 21 international contacts, coming from 14 countries.

The mailing lists in ecology were a French mailing list about ecology and evolution, SFEcodiff, which counted around 7,000 subscribers (<https://www.sfecologie.org/sfecodiff/>), and three international mailing lists about transportation ecology (Transenviro, Wftlistserv and IENE). Together, the Transenviro and Wftlistserv mailing lists (http://www.itre.ncsu.edu/CTE/Lists/index.asp) gathered about 600 contacts and the IENE mailing list (<http://www.iene.info/>) counted around 300 contacts.

Following the e-mail request, 77 scientific articles were sent to us by 21 experts. The research team considered that 50 of these articles were relevant to our scientific question. Studies that were excluded of the list mostly dealt with road-kills, habitat fragmentation, wildlife overpasses, green infrastructures in general, environmental impact assessment of LTI construction, naturalist inventories out of the temperate climatic zone and pedology of LTI verges. Those subjects were considered by the research team as subsidiary to the scientific question. On top of these 50 articles provided by experts, 23 scientific articles, known by the members of the research team to address our scientific question, were added to the list. Nevertheless, at that stage, few articles of the list were related to pipelines, waterways and railways. Thus, 29 relevant scientific articles were searched on Google Scholar in order to balance the proportion of articles of the list related to each LTI.

**Table A**: Articles of the test list identified by subject experts with indications of indexation and retrieval with the search strings in Web of Science Core Collection and Zoological Records. References in red are those that were not indexed and/or collected by the search string neither in Web of Science Core Collection nor in Zoological Records.

| **Reference** | **Web of Science Core Collection** | | **Zoological Records** | |
| --- | --- | --- | --- | --- |
|  | **Indexed** | **Collected** | **Indexed** | **Collected** |
| 1. Akbar KF, Hale WHG, Headley AD, Ashraf I. Evaluation of Conservation Status of Roadside Verges and Their Vegetation in North England. Pol J Ecol. 2010;58:459–67. | X | X |  |  |
| 2. Akbar KF, Hale WHG, Headley ADD. Floristic Composition and Environmental Determinants of Roadside Vegetation in North England. Pol J Ecol. 2009;57:73–88. | X | X |  |  |
| 3. Akbar KF, Headley AD, Hale WHG, Athar M. A Comparative Study of De-Icing Salts (Sodium Chloride and Calcium Magnesium Acetate) on the Growth of Some Roadside Plants of England. J Appl Sci Environ Manag. 2006;10:67–71. |  |  |  |  |
| 4. Arévalo JR, Otto R, Escudero C, Fernández-Lugo S, Arteaga M, Delgado JD, et al. Do anthropogenic corridors homogenize plant communities at a local scale? A case studied in Tenerife (Canary Islands). Plant Ecol. 2010;209:23–35. | X | X |  |  |
| 5. Arnett EB, Hayes JP. Bat Use of Roosting Boxes Installed under Flat-Bottom Bridges in Western Oregon. Wildl Soc Bull. 2000;28:890–4. | X | X | X |  |
| 6. Askins RA, Folsom-O’Keefe CM, Hardy MC. Effects of Vegetation, Corridor Width and Regional Land Use on Early Successional Birds on Powerline Corridors. PLoS ONE. 2012;7:e31520. | X | X | X | X |
| 7. Barnaud A, Kalwij JM, Berthouly-Salazar C, McGeoch MA, Jansen van Vuuren B. Are road verges corridors for weed invasion? Insights from the fine-scale spatial genetic structure of *Raphanus raphanistrum*. Weed Res. 2013;53:362–9. | X | X |  |  |
| 8. Barrientos R, Bolonio L. The presence of rabbits adjacent to roads increases polecat road mortality. Biodivers Conserv. 2009;18:405–18. | X | X | X | X |
| 9. Bellamy PE, Shore RF, Ardeshir D, Treweek R, Sparks TH. Road verges as habitat for small mammals in Britain. Mammal Rev. 2000;30:131–139. | X | X | X | X |
| 10. Benítez-López A, Alkemade R, Verweij PA. The impacts of roads and other infrastructure on mammal and bird populations: A meta-analysis. Biol Conserv. 2010;143:1307–16. | X | X | X | X |
| 11. Bolger DT, Scott TA, Rotenberry JT. Use of corridor-like landscape structures by bird and small mammal species. Biol Conserv. 2001;102:213–224. | X | X | X | X |
| 12. Brisson J, de Blois S, Lavoie C. Roadside as Invasion Pathway for Common Reed *Phragmites australis*. Invasive Plant Sci Manag. 2010;3:506–14. | X | X |  |  |
| 13. Brown GP, Phillips BL, Webb JK, Shine R. Toad on the road: Use of roads as dispersal corridors by cane toads *Bufo marinus* at an invasion front in tropical Australia. Biol Conserv. 2006;133:88–94. | X | X | X | X |
| 14. Busch DE, Smith SD. Mechanisms Associated With Decline of Woody Species in Riparian Ecosystems of the Southwestern U.S. Ecol Monogr. 1995;65:347–70. | X | X |  |  |
| 15. Cerboncini RAS, Roper JJ, Passos FC. Edge effects without habitat fragmentation? Small mammals and a railway in the Atlantic Forest of southern Brazil. Oryx. 2015;FirstView:1–8. |  |  |  |  |
| 16. Chelazzi G, Naziridis T, Benvenuti S, Ugolini A, Crivelli AJ. Use of river-wetland habitats in a declining population of the terrapin *Mauremys rivulata* along the Strymon River, northern Greece. J Zool. 2007;271:154–61. | X | X | X | X |
| 17. Christen D, Matlack G. The Role of Roadsides in Plant Invasions: a Demographic Approach. Conserv Biol. 2006;20:385–91. | X | X |  |  |
| 18. Clark BK, Clark BS, Munsterman WE, Homerding TR. Differential use of roadside fencerows and contiguous pastures by small mammals in southeastern Oklahoma. Southwest Nat. 1996;:54–59. | X | X | X | X |
| 19. Clevenger AP, Chruszcz B, Gunson KE. Highway mitigation fencing reduces wildlife-vehicle collisions. Wildl Soc Bull. 2001;29:646–53. | X | X |  |  |
| 20. Coles TF, Southey JM, Forbes I, Clough T. River wildlife databases and their value for sensitive environmental management. Regul Rivers Res Manag. 1989;4:179–89. |  |  | X | X |
| 21. Décamps H, Planty-Tabacchi AM, Tabacchi E. Changes in the hydrological regime and invasions by plant species along riparian systems of the Adour River, France. Regul Rivers Res Manag. 1995;11:23–33. | X | X |  |  |
| 22. Desserud P, Gates CC, Adams B, Revel RD. Restoration of foothills rough fescue grassland following pipeline disturbance in southwestern Alberta. J Environ Manage. 2010;91:2763–70. | X | X |  |  |
| 23. Clarke DJ, Pearce KA, White JG. Powerline corridors: degraded ecosystems or wildlife havens? Wildl Res. 2006;33:615–626. | X | X |  |  |
| 24. Dussault C, Ouellet J-P, Laurian C, Courtois R, Poulin M, Breton L. Moose Movement Rates Along Highways and Crossing Probability Models. J Wildl Manag. 2007;71:2338. | X | X | X | X |
| 25. Everson DA, Boucher DH. Tree species-richness and topographic complexity along the riparian edge of the Potomac River. For Ecol Manag. 1998;109:305–314. | X | X |  |  |
| 26. Fabritius H, Rönkä K, Ovaskainen O. The dual role of rivers in facilitating or hindering movements of the false heath fritillary butterfly. Mov Ecol. 2015;3:1–14. |  |  |  |  |
| 27. Fahrig L, Rytwinski T. Effects of roads on animal abundance: an empirical review and synthesis. Ecol Soc. 2009;14:21. | X | X |  |  |
| 28. Filibeck G, Cornelini P, Petrella P. Floristic analysis of a high-speed railway embankment in a Mediterranean landscape. Acta Bot Croat. 2012;71:229–248. | X | X |  |  |
| 29. Fortin C, Doucet J. [Small mammals and land taken by electricity pylons situated in the forest environment.]. Nat Can Quebec. 2008;132:32–40. |  |  | X | X |
| 30. Fortin C, Galois P, Ouellet M, Doucet GJ. [Use of electric pylon areas by amphibians and reptiles of the deciduous forest of Quebec.]. Nat Can Quebec. 2004;128:68–75. |  |  | X | X |
| 31. Graitson E, Hussin J, Parent G-H. Le rôle des voies ferrées dans la mise en place des reptiles en Belgique et dans quelques territoires adjacents (Nord et Nord-Est de la France, Grand-Duché de Luxembourg). Nat Belg. 2000;81:376–95. |  |  | X | X |
| 32. Graitson E. L’herpétofaune des bords de route en Wallonie: analyse de 101 stations. Nat Belg Les. 2006;87:73–80. |  |  | X | X |
| 33. Graitson E. Répartition et écologie des reptiles sur le réseau ferroviaire en Wallonie. Bull Société Herpétologique Fr. 2006;120:15–32. |  |  | X | X |
| 34. Greenberg CH, Crownover SH, Gordon DR. Roadside soils: A corridor for invasion of xeric shrub by nonindigenous plants. Nat Areas J. 1997;17:99–109. |  |  |  |  |
| 35. Grilo C, Sousa J, Ascensão F, Matos H, Leitão I, Pinheiro P, et al. Individual Spatial Responses towards Roads: Implications for Mortality Risk. PLoS ONE. 2012;7:e43811. | X | X |  |  |
| 36. Harvolk S, Symmank L, Sundermeier A, Otte A, Donath TW. Can artificial waterways provide a refuge for floodplain biodiversity? A case study from North Western Germany. Ecol Eng. 2014;73:31–44. | X | X |  |  |
| 37. Hansen MJ, Clevenger AP. The influence of disturbance and habitat on the presence of non-native plant species along transport corridors. Biol Conserv. 2005;125:249–59. | X | X |  |  |
| 38. Jacquemyn H, Looy KV, Breyne P, Honnay O. The Meuse river as a corridor for range expansion of the exotic plant species *Sisymbrium austriacum*: evidence for long-distance seed dispersal. Biol Invasions. 2009;12:553–61. | X | X |  |  |
| 39. Jansson R, Zinko U, Merritt DM, Nilsson C. Hydrochory increases riparian plant species richness: a comparison between a free-flowing and a regulated river. J Ecol. 2005;93:1094–103. | X | X |  |  |
| 40. Jodoin Y, Lavoie C, Villeneuve P, Theriault M, Beaulieu J, Belzile F. Highways as corridors and habitats for the invasive common reed *Phragmites australis* in Quebec, Canada. J Appl Ecol. 2008;45:459–66. | X | X |  |  |
| 41. Johnston FM, Johnston SW. Impacts of Road Disturbance on Soil Properties and on Exotic Plant Occurrence in Subalpine Areas of the Australian Alps. Arct Antarct Alp Res. 2004;36:201–7. | X | X |  |  |
| 42. Joly M, Bertrand P, Gbangou RY, White M-C, Dubé J, Lavoie C. Paving the Way for Invasive Species: Road Type and the Spread of Common Ragweed *Ambrosia artemisiifolia*. Environ Manage. 2011;48:514–22. | X | X |  |  |
| 43. Jones IL, Bull JW, Milner-Gulland EJ, Esipov AV, Suttle KB. Quantifying habitat impacts of natural gas infrastructure to facilitate biodiversity offsetting. Ecol Evol. 2014;4:79–90. | X | X |  |  |
| 44. Lavoie C, Jodoin Y, de Merlis AG. How did common ragweed *Ambrosia artemisiifolia L.* spread in Québec? A historical analysis using herbarium records. J Biogeogr. 2007;34:1751–61. | X | X |  |  |
| 45. Leblond M, Dussault C, Ouellet J-P, Poulin M, Courtois R, Fortin J. Management of Roadside Salt Pools to Reduce Moose–Vehicle Collisions. J Wildl Manag. 2007;71:2304. | X | X | X | X |
| 46. Le Viol I, Chiron F, Julliard R, Kerbiriou C. More amphibians than expected in highway stormwater ponds. Ecol Eng. 2012;47:146–54. | X | X | X | X |
| 47. Le Viol I, Julliard R, Kerbiriou C, de Redon L, Carnino N, Machon N, et al. Plant and spider communities benefit differently from the presence of planted hedgerows in highway verges. Biol Conserv. 2008;141:1581–90. | X | X | X | X |
| 48. Le Viol I, Mocq J, Julliard R, Kerbiriou C. The contribution of motorway stormwater retention ponds to the biodiversity of aquatic macroinvertebrates. Biol Conserv. 2009;142:3163–71. | X | X | X | X |
| 49. Li Y, Yu J, Ning K, Du S, Han G, Qu F, et al. Ecological effects of roads on the plant diversity of coastal wetland in the Yellow River Delta. ScientificWorldJournal. 2014;2014:952051. | X | X |  |  |
| 50. Lym RG, Nelson JA. Biological Control of Leafy Spurge *Euphorbia esula* with Aphthona spp. along Railroad Right-of-Ways. Weed Technol. 2000;14:642–6. | X | X |  |  |
| 51. Major RE, Smith D, Cassis G, Gray M, Colgan DJ. Are roadside strips important reservoirs of invertebrate diversity? A comparison of the ant and beetle faunas of roadside strips and large remnant woodlands. Aust J Zool. 1999;47:611. | X | X | X | X |
| 52. Merritt DM, Cooper DJ. Riparian vegetation and channel change in response to river regulation: a comparative study of regulated and unregulated streams in the Green River Basin, USA. Regul Rivers Res Manag. 2000;16:543–64. | X | X |  |  |
| 53. Meunier F, Fichet X, Verheyden C. [Use of motorway verges by raptors at different spatial and temporal scales]. Alauda. 2001;69:117–27. |  |  | X | X |
| 54. Meunier F, Gauriat C, Verheyden C, Jouventin P. Végétation des dépendances vertes autoroutières : influences d’un mode de gestion extensif et du milieu traversé. Rev Ecol Terre Vie. 1998;53:97–121. |  |  |  |  |
| 55. Meunier FD, Corbin J, Verheyden C, Jouventin P. Effects of landscape type and extensive management on use of motorway roadsides by small mammals. Can J Zool. 1999;77:108–17. | X | X | X | X |
| 56. Meunier FD, Verheyden C, Jouventin P. Bird communities of highway verges: Influence of adjacent habitat and roadside management. Acta Oecologica. 1999;20:1–13. | X | X | X | X |
| 57. Meunier FD, Verheyden C, Jouventin P. Use of roadsides by diurnal raptors in agricultural landscapes. Biol Conserv. 2000;92:291–298. | X | X | X | X |
| 58. Morelli F, Beim M, Jerzak L, Jones D, Tryjanowski P. Can roads, railways and related structures have positive effects on birds? – A review. Transp Res Part Transp Environ. 2014;30:21–31. | X | X |  |  |
| 59. Morelli F. Importance of road proximity for the nest site selection of the Red-backed shrike *Lanius collurio* in an agricultural environment in Central Italy. J Mediterr Ecol. 2011;11:21–29. |  |  | X | X |
| 60. Moroń D, Skórka P, Lenda M, Rożej-Pabijan E, Wantuch M, Kajzer-Bonk J, et al. Railway Embankments as New Habitat for Pollinators in an Agricultural Landscape. PLoS ONE. 2014;9:e101297. | X | X | X | X |
| 61. Munguira ML, Thomas JA. Use of Road Verges by Butterfly and Burnet Populations, and the Effect of Roads on Adult Dispersal and Mortality. J Appl Ecol. 1992;29:316. | X | X | X | X |
| 62. Naiman RJ, Decamps H, Pollock M. The Role of Riparian Corridors in Maintaining Regional Biodiversity. Ecol Appl. 1993;3:209–12. | X | X | X | X |
| 63. Nilsson C, Grelsson G, Johansson M, Sperens U. Patterns of Plant Species Richness Along Riverbanks. Ecology. 1989;70:77–84. | X | X |  |  |
| 64. Nilsson C, Svedmark M. Basic Principles and Ecological Consequences of Changing Water Regimes: Riparian Plant Communities. Environ Manage. 2002;30:468–80. | X | X |  |  |
| 65. Noordijk J, Delille K, Schaffers AP, Sýkora KV. Optimizing grassland management for flower-visiting insects in roadside verges. Biol Conserv. 2009;142:2097–103. | X | X | X | X |
| 66. O’Farrell PJ, Milton SJ. Road Verge and Rangeland Plant Communities in the Southern Karoo: Exploring What Influences Diversity, Dominance and Cover. Biodivers Conserv. 2006;15:921–38. | X | X |  |  |
| 67. Penone C, Machon N, Julliard R, Le Viol I. Do railway edges provide functional connectivity for plant communities in an urban context? Biol Conserv. 2012;148:126–33. | X | X |  |  |
| 68. Peris S, Morales J. Use of passages across a canal by wild mammals and related mortality. Eur J Wildl Res. 2004;50:67–72. | X | X | X | X |
| 69. Perry MC, Osenton PC, Fallon FW, Fallon JE. Optimal management strategies for biodiversity within a powerline right-of-way. In: Part II. Vegetation Management. New Orleans, Louisiana, USA; 1997. p. 133–9. |  |  | X | X |
| 70. Peterson E, Hansen E, Kanaskie A. Spatial relationship between *Phytophthora ramorum* and roads or streams in Oregon tanoak forests. For Ecol Manag. 2014;312:216–24. | X | X |  |  |
| 71. Planty-Tabacchi A-M, Tabacchi E, Naiman RJ, Deferrari C, Décamps H. Invasibility of Species-Rich Communities in Riparian Zones. Conserv Biol. 1996;10:598–607. | X | X |  |  |
| 72. Prunier JG, Kaufmann B, Léna J-P, Fenet S, Pompanon F, Joly P. A 40-year-old divided highway does not prevent gene flow in the alpine newt *Ichthyosaura alpestris*. Conserv Genet. 2014;15:453–68. | X | X | X | X |
| 73. Pysek P, Prach K. Plant Invasions and the Role of Riparian Habitats: A Comparison of Four Species Alien to Central Europe. J Biogeogr. 1993;20:413–20. | X | X |  |  |
| 74. Rahlao SJ, Milton SJ, Esler KJ, Barnard P. The distribution of invasive *Pennisetum setaceum* along roadsides in western South Africa: the role of corridor interchanges: Corridor interchange and plant invasion. Weed Res. 2010;50:537–43. | X | X |  |  |
| 75. Rautenstrauch KR, Krausman PR. Preventing Mule Deer Drownings in the Mohawk Canal, Arizona. Wildl Soc Bull. 1989;17:280–6. | X |  | X |  |
| 76. Redon (de) L, Le Viol I, Jiguet F, Machon N, Scher O, Kerbiriou C. Road network in an agrarian landscape: Potential habitat, corridor or barrier for small mammals? Acta Oecologica. 2015;62:58–65. | X | X |  |  |
| 77. Rentch JS, Fortney RH, Stephenson SL, Adams HS, Grafton WN, Anderson JT. Vegetation-site relationships of roadside plant communities in West Virginia, USA. J Appl Ecol. 2005;42:129–38. | X | X |  |  |
| 78. Richardson DM, Holmes PM, Esler KJ, Galatowitsch SM, Stromberg JC, Kirkman SP, et al. Riparian vegetation: degradation, alien plant invasions, and restoration prospects. Divers Distrib. 2007;13:126–39. | X | X |  |  |
| 79. Ries L, Debinski DM, Wieland ML. Conservation value of roadside prairie restoration to butterfly communities. Conserv Biol. 2001;15:401–411. | X | X | X | X |
| 80. Ruiz-Capillas P, Mata C, Malo JE. Road verges are refuges for small mammal populations in extensively managed Mediterranean landscapes. Biol Conserv. 2013;158:223–9. | X | X | X | X |
| 81. Russell KN, Ikerd H, Droege S. The potential conservation value of unmowed powerline strips for native bees. Biol Conserv. 2005;124:133–48. | X | X | X | X |
| 82. Saarinen K, Valtonen A, Jantunen J, Saarnio S. Butterflies and diurnal moths along road verges: Does road type affect diversity and abundance? Biol Conserv. 2005;123:403–12. | X | X | X | X |
| 83. Sabo JL, Sponseller R, Dixon M, Gade K, Harms T, Heffernan J, et al. Riparian Zones Increase Regional Species Richness by Harboring Different, Not More, Species. Ecology. 2005;86:56–62. | X | X | X | X |
| 84. Sakchoowong W, Jaitrong W, Ogata K. Comparison of Ground-Ant Diversity Between Natural Forests and Disturbed Forests Along a Natural Gas Pipeline Transect in Thong Pha Phum National Park, Kanchanaburi Province. Kasetsart J Nat Sci. 2009;43:64–73. |  |  | X | X |
| 85. Samways MJ, Osborn R, Carliel F. Effect of a highway on ant (Hymenoptera: Formicidae) species composition and abundance, with a recommendation for roadside verge width. Biodivers Conserv. 1997;6:903–13. | X | X | X | X |
| 86. Silverman B, Horn DJ, Purrington FF, Gandhi KJK. Oil Pipeline Corridor Through an Intact Forest Alters Ground Beetle (Coleoptera: Carabidae) Assemblages in Southeastern Ohio. Environ Entomol. 2008;37:725–33. | X | X | X | X |
| 87. Rutkovska S, Pučka I, Evarts-Bunders P, Paidere J. The role of railway lines in the distribution of alien plant species in the territory of Daugavpils City (Latvia). Est J Ecol. 2013;62:212. |  |  |  |  |
| 88. Tikka PM, Hogmander H, Koski PS. Road and railway verges serve as dispersal corridors for grassland plants. Landsc Ecol. 2001;16:659–66. | X | X |  |  |
| 89. Trewhella W, Harris S. The Effect of Railway Lines on Urban Fox *Vulpes vulpes* Numbers and Dispersal Movements. J Zool. 1990;221:321–6. | X | X | X | X |
| 90. Tryjanowski P, Sparks TH, Jerzak L, Rosin ZM, Skórka P. A Paradox for Conservation: Electricity Pylons May Benefit Avian Diversity in Intensive Farmland: Paradox of the impact of pylons. Conserv Lett. 2014;7:34–40. | X | X |  |  |
| 91. Valtonen A, Jantunen J, Saarinen K. Flora and lepidoptera fauna adversely affected by invasive *Lupinus polyphyllus* along road verges. Biol Conserv. 2006;133:389–96. | X | X | X | X |
| 92. Valtonen A, Saarinen K, Jantunen J. Intersection reservations as habitats for meadow butterflies and diurnal moths: Guidelines for planning and management. Landsc Urban Plan. 2007;79:201–9. | X | X | X | X |
| 93. Vandevelde J-C, Bouhours A, Julien J-F, Couvet D, Kerbiriou C. Activity of European common bats along railway verges. Ecol Eng. 2014;64:49–56. | X | X | X | X |
| 94. Vandevelde J-C, Penone C, Julliard R. High-speed railways are not barriers to Pyronia tithonus butterfly movements. J Insect Conserv. 2012;16:801–3. | X | X | X | X |
| 95. vanDorp D, Schippers P, vanGroenendael JM. Migration rates of grassland plants along corridors in fragmented landscapes assessed with a cellular automation model. Landsc Ecol. 1997;12:39–50. | X | X |  |  |
| 96. Vasconcelos PB, Araujo GM, Bruna EM. The role of roadsides in conserving Cerrado plant diversity. Biodivers Conserv. 2014;23:3035–50. | X | X |  |  |
| 97. Vermeulen HJW, Opdam PFM. Effectiveness of roadside verges as dispersal corridors for small ground-dwelling animals: A simulation study. Landsc Urban Plan. 1995;31:233–48. |  |  | X | X |
| 98. Vermeulen HJ. Corridor function of a road verge for dispersal of stenotopic heathland ground beetles Carabidae. Biol Conserv. 1994;69:339–349. | X | X | X | X |
| 99. Von Der Lippe M, Kowarik I. Long-Distance Dispersal of Plants by Vehicles as a Driver of Plant Invasions. Conserv Biol. 2007;21:986–96. | X | X |  |  |
| 100. Wagner DL, Ascher JS, Bricker NK. A Transmission Right-of-Way as Habitat for Wild Bees (Hymenoptera: Apoidea: Anthophila) in Connecticut. Ann Entomol Soc Am. 2014;107:1110–20. | X | X | X | X |
| 101. Wrzesień M, Denisow B. The usable taxons in spontaneous flora of railway areas of the central-eastern part of Poland. Acta Agrobot. 2006;59:95–108. |  |  |  |  |
| 102. Yost AC, Wright RG. Moose, caribou, and grizzly bear distribution in relation to road traffic in Denali National Park, Alaska. Arctic. 2001;54:41–8. | X | X | X | X |
