# Supplementary material for: Can linear transportation infrastructure verges constitute a habitat and/or a corridor for vascular plants in temperate ecosystems? A systematic review
Source: Environ Evid. 2024 Mar 16;13:4. doi: 10.1186/s13750-024-00328-3 (PMC11376103; doi:10.1186/s13750-024-00328-3)
Supplement: Supplementary file 3 — Additional file 3. Search strings used for search engines. Search strings that were used for search engines Google Scholar, BASE, and CORE. [file 13750_2024_328_MOESM3_ESM.docx]

**Additional File 3: Search strings used for search engines.** Search strings that were used for search engines Google Scholar, BASE, and CORE.

Search strings used for Google Scholar:

- (riparian OR riverside OR riverbank OR waterway OR canal) (corridor OR dispersal OR habitat OR refuge OR abundance OR richness OR diversity OR composition OR community OR communities)
- (road OR roadside OR highway) (corridor OR dispersal OR habitat OR refuge OR "right of way" OR "rights of ways" OR verge OR abundance OR richness OR composition OR diversity OR community OR communities)
- (railway OR railroad) (corridor OR dispersal OR habitat OR refuge OR "right of way" OR "rights of ways" OR verge OR abundance OR richness OR composition OR diversity OR community OR communities)
- (pipeline) (corridor OR dispersal OR habitat OR refuge OR "right of way" OR "rights of ways" OR verge OR abundance OR richness OR composition OR diversity OR community OR communities)
- ("power line" OR "power lines" OR "transmission line" OR "transmission lines") (corridor OR dispersal OR habitat OR refuge OR "right of way" OR "rights of ways" OR verge OR abundance OR richness OR composition OR diversity OR community OR communities)

Google Scholar doesn’t understand parenthesis but we kept them in the equation to illustrate that Scholar automatically inserts “AND” between the two blocks which are in parenthesis.

Search strings used for BASE and CORE:

- (riparian OR riverside OR riverbank OR waterway OR canal) **AND** (corridor OR dispersal OR habitat OR refuge OR abundance OR richness OR diversity OR composition OR community OR communities)
- (road OR roadside OR highway) **AND** (corridor OR dispersal OR habitat OR refuge OR "right of way" OR "rights of ways" OR verge OR abundance OR richness OR composition OR diversity OR community OR communities)
- (railway OR railroad) **AND** (corridor OR dispersal OR habitat OR refuge OR "right of way" OR "rights of ways" OR verge OR abundance OR richness OR composition OR diversity OR community OR communities)
- (pipeline) **AND** (corridor OR dispersal OR habitat OR refuge OR "right of way" OR "rights of ways" OR verge OR abundance OR richness OR composition OR diversity OR community OR communities)
- ("power line" OR "power lines" OR "transmission line" OR "transmission lines") **AND** (corridor OR dispersal OR habitat OR refuge OR "right of way" OR "rights of ways" OR verge OR abundance OR richness OR composition OR diversity OR community OR communities)
