# Supplementary material for: Can linear transportation infrastructure verges constitute a habitat and/or a corridor for vascular plants in temperate ecosystems? A systematic review
Source: Environ Evid. 2024 Mar 16;13:4. doi: 10.1186/s13750-024-00328-3 (PMC11376103; doi:10.1186/s13750-024-00328-3)
Supplement: Supplementary file 4 — Additional file 4. Searches for literature summary. Summary of all the searches for literature with dates of search and number of articles found. [file 13750_2024_328_MOESM4_ESM.docx]

**Additional File 4: Searches for literature summary.** Summary of all the searches for literature with dates of search and number of articles found.

| **Tools used for literature search** | | **Date(s) of search** | **Number of references found** |
| --- | --- | --- | --- |
| Publication database | Web of Science Core Collection | 27 April 2015  15 June 2018  3 March 2021 | 44,581  16,831  17,494 |
|  | Zoological Records | 1 February 2016  15 June 2015  3 March 2021 | 19,030  2,130  1,458 |
| Search engine | Google Scholar | 4-9 March 2016  6 November 2018 | 100  100 |
|  | BASE (Bielefeld Acadamic Search engine) | 7 November 2018 | 100 |
|  | CORE | 8 November 2018 | 100 |
| Specialist website | IENE Infra Eco Network Europe (conference 2016) <http://www.iene.info/> | 26 November 2018 | 33 |
|  | KHEOPS <https://www.kheops.ca/mission/> | 29 November 2018 | 0 |
|  | ICOET International Conference on Ecology and Transportation (conference 2017) <http://www.icoet.net/ICOET_2019/index.asp> | 29 November and 3 December 2018 | 14 |
|  | ANET Australasian Network for Ecology and Transportation <http://www.ecologyandtransport.com/anet-2018/> | 29 November 2018 | 0 |
|  | EWT Endangered Wildlife Trust <https://www.ewt.org.za/index.html> | 29 November 2018 | 0 |
|  | IAIA International Association for Impact Assessment <http://www.iaia.org/index.php> | 3 December 2018 | 0 |
|  | SIFEE Secrétariat francophone pour l’évaluation environnementale <https://www.sifee.org/ressources/actes-des-colloques/actes-du-colloque-international-de-antananarivo> | 3 December 2018 | 0 |
|  | ITTECOP Infrastructures de Transport Terrestre, Ecosystèmes et Paysage <http://www.ittecop.fr/> | 28-29 November 2018 | 18 |
|  | MTES Ministère de la transition écologique et solidaire | 4 December 2018 | 0 |
|  | DTRF Documentation des Techniques Routières Françaises <http://dtrf.setra.fr/> | 4 December 2018 | 0 |
|  | Cerema <http://www.cerema.fr/fr/evenements/dependances-vertes-infrastructures-quels-enjeux-quelles> | 4 December 2018 | 17 |
| Journal website | Special issue Frontiers In <https://www.frontiersin.org/research-topics/5170/integrating-transport-infrastructures-with-living-landscapes> | 3 December 2018 | 14 |
| Documents spontaneously sent to us | | 8 October 2018 and 6 November 2018 | 2 |
|  |  | 20 October 2021 and 15 November 2021 | 14 |
| Email requests sent to a list of 1902 people | | 21 April 2015 | 495 |
| **TOTAL** | | | **102,531** |
