# Supplementary material for: Can linear transportation infrastructure verges constitute a habitat and/or a corridor for vascular plants in temperate ecosystems? A systematic review
Source: Environ Evid. 2024 Mar 16;13:4. doi: 10.1186/s13750-024-00328-3 (PMC11376103; doi:10.1186/s13750-024-00328-3)
Supplement: Supplementary file 6 — Additional file 6. Narrative tables. Tables summarizing key results of the studies included in the narrative syntheses. [file 13750_2024_328_MOESM6_ESM.docx]

**Additional file 6: Narrative tables.** Tables summarizing key results of the studies included in the narrative syntheses.

**Table S1: Key results of the 127 studies included in the narrative synthesis of the question: Do LTI verge management practices increase, decrease, or have no effect on tracheophyte biodiversity in LTI verges? (question Q1)**

| **[id]** | **Reference** | **Risk of bias** | **Country** | **Biological group** | **LTI** | **LTI verge** | **Comparison** | **Key results** | **Grp.^§^** |
| --- | --- | --- | --- | --- | --- | --- | --- | --- | --- |
| 3453 | Aguiar et al., 2018 | Medium | Portugal | Riparian woody species | River | Riverbanks | *Stream flow regulation*  Free-flowing river vs river downstream of a damn | Hydrological regime (natural vs regulated) did not influence the occurrence of riparian guilds. The relative abundance however varied with the hydrological regime for all the riparian guilds (obligate riparian, water-stress tolerant, deciduous competitive and Mediterranean evergreen). (text p. 7, table S5.1) | a |
| 2003 | Asdonk et al., 2019 | Medium | Germany | Riparian vegetation | River | Riversides | *Stream flow regulation*  Before vs after disconnection from tidal regime (construction of a sluice) | Significantly more threatened species, less non-native species and more species per plot (except for the reed vegetation category) were found in 1951 than in 2016. Evenness on the other hand was lowest for reed vegetation in 1951. (text p. 3-4, Fig. 3A-D)  Species composition also significantly changed between 1951 and 2016. (text p. 5, Fig. 4) | a |
| WOS_1512 | Azami et al., 2004 | Medium | Japan | Riparian vegetation  *Robinia pseudoacacia*  *Euptelea*  *polyandra* | River | River floodplain | *Stream flow regulation*  Downstream of dam vs tributary with no dam | Compared to the reference area with no dam, the area downstream of the dam reservoir had significantly higher ratios of vegetated area and notably forested area. (text p. 555, Fig. 5)  *Robinia pseudoacacia* and *Euptelea polyandra* had significantly higher ratios among other plant species in the downstream area. (text p. 555) | a |
| WOS_2298 | Beauchamp et al., 2007 | Medium | USA | Herbaceous vegetation | River | River floodplain with *Populus-Salix* stands | *Stream flow regulation*  Unregulated reach vs dam-regulated reach | Regulated plots showed lower herbaceous cover, perennial cover and richness, annual cover, wetland species cover and upland species cover compared with unregulated reaches. Herbaceous richness, annual richness, wetland species richness and upland species richness on the other hand did not differ between regulated and unregulated reach plots. (table 1)  The proportion of annual species in the ﬂora was signiﬁcantly greater in the  regulated reach than the unregulated reach and conversely, the proportion of  perennial species was greater in the unregulated reach. (text p. 196) | a |
| WOS_2301 | Beauchamp et al., 2008 | Medium | USA | Herbaceous vegetation | River | River floodplain with *Populus-Salix* stands | *Stream flow regulation U*nregulated reach vs dam-regulated reach | Regulated reach plots showed significantly less cover, species richness and  diversity for all seasons and year (except in spring 2001 for richness and  diversity) compared with unregulated reach plots. (Fig. 5b-d)  For functional groups (Nitrogen-fixer, No N fixation, Annual, Clonal perennial, Non-clonal perennial, Hydric, Mesic, Xeric) or origin (Introduced, Native), differences in species density were often found, especially during the summer and in every case where there was a significant difference in richness between reaches, it was always higher in the unregulated reach. (text p. 761, see table 3 for detailed results)  During spring 2001, exotic/native species represented a greater proportion of the vegetation of the unregulated/regulated sites respectively, but the differences were not significant for the three other sampling periods.  Differences in proportions for certain functional groups in specific  season/year combinations were also found. (text p. 763, see Fig. 6 for all  comparisons) | a |
| WOS_2412 | Bejarano et al., 2013 | Medium | Spain | Riparian woody species | River | Riversides | *Stream flow regulation*  Periods pre- vs post-dam construction | Pre-dam woody species Shannon’s diversity index was signiﬁcantly higher than for the post-dam period. Patterns of diversity varied however between initial-transitional vegetation guilds and late-successional guilds and with the geomorphological characteristics of the sites. (see text p. 1244-1246, Fig. 5 for detailed results) | a |
| WOS_3267 | Bombino et al., 2014 | Medium | Italy | Riparian vegetation | River | Riversides | *Stream flow regulation*  Transects upstream of check dam vs downstream of check dam vs intermediate non-disturbed control site | Transects located upstream of check dams showed significantly higher alpha  diversity than the intermediate transects, while the difference across sites was not significant between downstream and intermediate transects. (text p. 683, table 2)  Differences in the alpha diversity of specific vegetation layer were also  found, with respectively higher diversity of herbaceous and shrub layers for  the upstream transects, and higher diversity of shrubs and trees for the  downstream transects, compared to the intermediate transects. (table 5)  The ratio of alien species richness over native species richness was higher in  upstream transects than in intermediate transects. Values for the downstream  transects however were similar to the controls. (table 4) | a |
| 918 | Chen et al., 2020 | Medium | China | Riparian vegetation | River | Riversides | *Stream flow regulation*  Inundated gradient in drawdown zone of the dam reservoir and non-flooded upland zone | Values obtained for Shannon, Simpson, Pielou indices and species richness did  not vary significantly along the inundated gradient, whereas community height,  community biomass and total coverage did, with the highest values found for intermediate levels of inundation. (text p. 4, Fig. 2) | a |
| 1532 | Czortek et al., 2020 | Medium | Poland | Understory riparian vegetation | River | Riversides in urban areas | *Stream flow regulation*  Plots pre- vs post-dam construction | Post-dam *Galio sylvatici-Carpinetum* and *Salici-Populetum* plots had significantly lower species richness and higher alien species understory cover in comparison to the pre-dam sampling period. The differences between the two periods in *Salicetum triandro-viminalis* and *Querco-Ulmetum minoris* plots however were not significant for neither measure. | a |
| WOS_7816 | Bejerano et al., 2012 | Medium | Spain | Riparian woody species | River | Riverbanks from channel to terrace top or hillslope with relatively mature woody vegetation | *Stream flow regulation*  Pre-dam vs post-dam construction | There were significant differences in the relative proportion of the riparian  guilds that established during the pre-dam and the post-dam periods. (see text  p. 451 and Fig. 5 for a full description)  Pre–Post comparisons of Shannon’s index measuring woody species diversity  did not however indicate any statistically significant differences for most of  the guilds. (text p. 452, Fig. 7) | a |
| WOS_8487 | Elderd, 2003 | Medium | USA | Riparian vegetation  *Mimulus guttatus* | River | Riverside 0-10m from channel | *Stream flow regulation*  Transects upstream of dams, downstream of dams or with no dam | There were significant differences in overall herbaceous species coverage  across flooding regimes, with an increased herbaceous species coverage below  dams as compared to above dams and natural flowing streams. (text p. 1615, Fig. 2)  Naturally flowing streams had significantly greater canopy coverage than  streams above dams and below dams, but coverage did not differ between above and below dam sites. (text p. 1615)  Forb species composition also showed significant differences with flooding  regimes, although the differences between above and below dam sites were  significant only when comparing plots at 0m from the stream channel and not at the 5m or 10m distance. (text p. 1616, Fig. 5)  The distribution of the riparian species *Mimulus guttatus* was significantly  affected by the presence of dams and occurred less frequently in survey plots  above dams as compared to below-dams or natural streams survey plots. (text p. 1617) | a |
| WOS_11298 | Greet et al., 2013 | Medium | Australia | Riparian vegetation | River | Riverbanks | *Stream flow regulation*  Gradient of flow regulation (from unregulated to seasonally  inverted) | Greater proportions of exotic taxa and fewer native woody taxa were observed  with increasing level of regulation both at the site and quadrat level. At the  quadrat level, more regulation was also associated with greater number of  exotic ‘dry’ short-lived taxa. The interaction between the effect of regulation  and the season was also significant for multiple variables at both levels.  (text p. 690, table 2-3, Fig. 3-4)  Regarding species composition, similarity was greater between sites within  regulation types than between sites of different regulation types, with native  taxa generally more common at natural sites and exotic taxa more common at  inverted sites. (text p. 690-691, table 4) | a |
| WOS_11300 | Greet et al., 2013 | Medium | Australia | Soil seed bank | River | Riverbanks with a remnant native overstorey | *Stream flow regulation*  Gradient of flow regulation (from unregulated to seasonally  inverted) | Many more seedlings and taxa germinated from the seed banks of regulated  (moderate and inverted) sites than unregulated sites, with higher  numbers of exotic, exotic ‘dry’ taxa in regulated sites and higher numbers of  native, native ‘wet’ taxa in moderately regulated sites only.  (text p. 160, table 2, Fig 2a-b)  The level of regulation also explained differences of seed bank compositions, with moderately regulated and inverted sites having closer seed bank compositions than with the unregulated sites. (text p. 161, table 3) | a |
| EM_250 | Huc et al., 2015 | Low | France | Soil seed bank | River | Riversides | *Stream flow regulation*  Before vs after artificial flooding including an unaffected control site | Species richness in soil seed banks significantly decreased in all sites affected by flood events. | a |
| 12 | Lozanovska et al., 2020 | Medium | Portugal | Riparian woody vegetation | River | Riversides | *Stream flow regulation*  Gradient in degree of regulation including unregulated sites | In the reservoir case study, ‘disturbance adapted’ and ‘disturbance favoured’  riparian guilds showed an increase in cover in regulated sites, while  ‘disturbance resilient’ or ‘poorly disturbance-adapted’ guilds where more  present in unregulated sites. A similar pattern was observed in the  run-of-river case study except that ‘disturbance-resilient’ guild only occurred  in regulated sites. (text p. 7) | a |
| ACTU_ZR_F_49 | Stephens, 2017 | Low | USA | Riparian vegetation | River | Riverbanks | *Stream flow regulation*  Before vs after dam removal (and control not affected by dam removal) | Two years after dam removal, the vegetation structure (tree, shrub and ground  strata respectively) was similar to one year before. (text p. 333, Fig. 2) | a |
| 798 | Su et al., 2020 | Medium | China | Riparian vegetation | River | Undisturbed riparian zones | *Stream flow regulation*  Dam regulated river exposed to unnatural flooding vs free flowing river exposed to natural flooding | Both total species richness and species richness of different groups (tree +  shrubs, forbs + ferns, graminoids, annuals + biennials, perennials) were  significantly lower in the sites exposed to unnatural flooding regimes.  Except for the percentage cover of trees and shrubs, total cover and cover for  the other groups did not significantly differ between the sites exposed to  natural or unnatural flooding regimes. (text p. 5, Fig. 2b)  Guild composition of the two flooding regimes also differed significantly.  Guild richness and functional richness were significantly lower in the UFRZ,  while functional dispersion was similar between the UFRZ and the NFRZ.  (text p. 6, table 3)  Significant differences in guild abundance and species redundancy were also  found for various guilds. (text p. 6, see Fig. 5 for detailed results) | a |
| WOS_29729 | Su et al., 2012 | Medium | China | Riparian vegetation | River | Drawdown zone | *Stream flow regulation*  Pre-upland drawdown zone vs adjacent upland  Pre-riparian drawdown zone vs natural riparian zone  (drawdown zones associated with dam reservoir) | In the pre-upland drawdown zones, total cover was significantly lower but  transformed species richness was significantly higher than in the adjacent  upland. In terms of species composition, significant differences in the number  of species and proportion of cover for most groups were also found, with  overall a greater representation of annuals and biennials as well as ferns,  forbs and graminoids in the pre-upland drawdown zone. (see table 1 for detailed results)  In the pre-riparian drawdown zone, total species richness was significantly lower than in the natural riparian zone, while total cover was similar. Differences in species composition were also found with a general decrease in species richness for all morphology and life cycle groups and a decrease in the  proportion cover of trees and shrubs. (see table 1 for detailed results) | a |
| 2446 | Yang et al., 2019 | Medium | China | Riparian vegetation | River | Dam reservoir riparian zone | *Stream flow regulation*  Artificial flooding exposure in water-level ﬂuctuation zone of dam reservoir | Species richness was significantly higher in the control zone with no exposure  to artificial flooding, while biomass was significantly smaller. Shannon and  Simpson diversity indices on the other hand were similar across the flooding  regimes. (text p. 665, table 2) | a |
| 1883 | Zhang et al., 2019 | Medium | China | Riparian vegetation | River | Dam reservoir riparian zone | *Stream flow regulation*  Riparian zone exposed to artificial flooding vs upland zone | Species richness was significantly higher in the Upland zone (never flooded) than that in the Reservoir Riparian Zone. (text p. 12) | a |
| WOS_242 | Aguiar et al., 2001 | Medium | Portugal | Riparian vegetation | River | Riverbanks | *Channelization*  Channelized river segments vs ‘natural’ reaches | Richness and cover of exotic species were significantly greater in the channelized inner-bank plots than they were in the corresponding ‘natural’ ones. A higher richness but lower cover of natives were also found for channelized inner-bank plots. The outer bank plots of channelized sites on the other hand were characterized by lower cover of exotic and native species.  (table 1) | b |
| ACTU_WOS_F_2245 | Foard et al., 2016 | Low | USA | *Quercus nigra*  *Quercus pagoda*  *Quercus michauxii*  *Quercus lyrata*  Woody species (surrounding sampled trees) | River | Riparian forest | *Channelization*  Channelized vs non-channelized sites | Trees and stands characteristics (tree height, DBH, % Dieback, Live Crown Ratio, % Canopy missing) did not differ significantly between non-invaded/channelized sites and non-invaded/non-channelized sites. (text p. 406, table 1)  Species richness and Simpson’s diversity index did not differ significantly between non-invaded/channelized sites and non-invaded/non-channelized sites. (text p. 406, table 1)  Wetland prevalence in non-invaded/channelized sites and non-invaded/non-channelized sites were not significantly different (text p. 406) | b |
| WOS_9732 | Francis & Hoggart, 2008 | Medium | UK | Riparian vegetation | River | River walls or foreshore | *Channelization*  River walls vs intertidal foreshore along urban river | River walls were significantly more species rich than intertidal foreshore  plant communities along the river. (text p. 374) | b |
| 2789 | Stepien et al. 2019 | Low | Poland | Riparian vegetation | River | River floodplain | *Channelization*  Pre-dredging vs post-dredging including control undredged sites | The number of species significantly increased on the river terrace the year after dredging. Differences were no longer significant after two years however.  (text p. 7)  Similarly, the mean cover at the impact site increased the first year after  dredging while cover at the control site remained stable. However, it decreased  between the first and second year after dredging such that differences were no  longer significant. (text p. 9, table 2, Fig. 6) | b |
| WOS_1539 | Baattrup-Pedersen et al., 2000 | Medium | Denmark | Vascular plants | River | Riverbanks | *River restoration*  Before vs after stream restoration project | Cover by non-riparian gramineous species decreased from approximately 40% to 5%, while cover by riparian gramineous species increased significantly but only on the southern bank slope. Cover by non-riparian herbaceous species did not change, while cover by riparian herbaceous species decreased after restoration. (text p. 19, Fig. 3b-c)  The mean number of non-riparian gramineous species decreased after restoration, whereas the mean number of riparian gramineous species increased. In contrast, the mean number of non-riparian herbaceous species remained constant on both banks, whereas the mean number of riparian herbaceous species either remained constant (northern bank) or decreased (southern bank). (text p. 19, Fig. 3b-c) | c |
| 3766 | Bauer et al., 2018 | Medium | Germany | Riparian plants (including invasive *Impatiens glandulifera***, *Solidago canadensis***, *Solidago gigantea***) | River | Riverbanks | *River restoration*  - creation of gravel groynes  - deposition of sand and loam  - riprap embankment removal  - unrestored upstream riparian sections | The riparian vegetation recovered fastest on banks with gravel groynes but there were no differences between the three types of restoration sites as well as their control after 4 years. (text p. 4, Fig 3)  There was no difference between control plots and restored sites in target and reed coverage. (text p. 6, Fig 6)  After 4 years, species composition in restored sites was more similar to controls compared with that after 2 years. Species composition also differed between gravel and sand sites in both years and gravel sites were more heterogeneous than sand sites over time and among measures. (text p. 5) | c |
| ACTU_WOS_F_2596 | Gothe et al., 2016 | Medium | Austria  Czech Republic  Denmark  Finland  Netherlands  Poland  Sweden  Switzerland | Riparian vegetation | River | River floodplain | *River restoration*  Types of restoration:  - stream channel widening  - recreating instream structure  - flow restoration  - re-meandering  - reconnection of side channels vs degraded sites  Large vs small restoration projects: for the different types of restoration,  depending on the extent of stream restoration | No general responses of the taxonomic diversity indices were detected in  response to the different restoration extent and measures. (text p. 83)  However, several of the response ratios on the community weighted means (CMW) changed significantly depending on restoration extent and the specific  restoration measures applied. The greatest number of significant responses was  detected in response to restorations aiming at widening the stream channels. (see table 4 for detailed results)  Larger and smaller restoration projects had different significant effects on CMWs. (see Fig. 1 for detailed results) | c |
| WOS_17969 | Liebrand et al., 1996 | Medium | Netherlands | Vascular plants | River | River dykes grasslands | *River restoration*  Different seed mixtures (see table 1) and reconstruction methods for river dykes: unmodified, intact sods, former topsoil, former subsoil, imported clay | Species richness was significantly lower on imported clay than in the other  four methods of reconstruction. (text 319, Fig. 1)  Reconstruction methods also had a significant impact on species composition.  (see table 3 for results for the most important syntaxonomical elements) | c |
| ACTU_WOS_F_972 | Modrak et al., 2017 | Medium | Germany | Riparian vegetation | River | Riverbanks | *River restoration*  Restored reaches vs non-restored reaches | The number of species per plot was higher in restored sections of large lowland river floodplains (FTP6) compared with non-restored sections, while no difference was observed for large mountain river floodplains (FTP3). (table 3)  Species composition varied between restored and non-restored areas for both floodplain types. Species pools showed higher numbers in restored areas of tolerators for both dry and wet conditions in contrast to species with average moisture preferences, as well as more species indicating regular flooding for FTP3 and FTP6. On restored areas of FTP6, there were also an increase of the abundance of ruderals as well as a decrease in species indicative of eutrophication. (text p. 6-7, table 4) | c |
| 1157 | Richards et al., 2020 | Medium | UK | Vascular plants | River | River floodplain | *River restoration*  pre- vs post-restoration period (lowering of flood defense banks) | The species richness of the sampled plant communities differed significantly  between survey years. Yet, pairwise comparisons revealed only significant  differences with pre-restoration data for the year 2011, showing a temporary increase. (text p. 9, Fig. 4)  Community composition changed between the pre- and post-restoration periods since all pairs of years were significantly different, with an increase in  moisture tolerant plants and a decline of taxa with stronger ruderal traits.  (text p. 9-10, Fig. 5, table 1) | c |
| WOS_26158 | Rohde et al., 2005 | Medium | Switzerland | Vascular plants | River | Riverbanks | *River restoration*  Restored sites (river widening projects) vs near-natural reference sites | Species richness was lower in the restored Emme site than in the corresponding  near-natural reference along the Sense river. At the restored site of the Moesa  (G) however species richness was signiﬁcantly higher than at the corresponding near-natural site (Moesa (C)). For the restored sites along the Rhône, Thur and Moesa (L), no significant differences with corresponding near-natural sites were found. (text p. 1087, table 8)  A similar pattern was found for the number of ﬂoodplain-dependent species *sensu lato*. For the number of ﬂoodplain-dependent species *sensu stricto*, the restored site at Emme and Thur showed significantly less riparian species of this class compared with the corresponding near-natural site. When considering additional typical riparian species, only the restored Emme site had a significantly lower number of riparian species than the near-natural site. (table 8)  Sites did not differ with regard to the number or proportion of alien species  however. (table 8) | c |
| ACTU_WOS_F_4220 | Strob et al., 2015 | Medium | Germany | Riparian vegetation | River | Riverbanks | *River restoration*  Restoration measures  - embankment removal  - sand input  - gravel addition  Restored areas vs non-restored areas | Vegetation cover was significantly lower on plots with gravel than on those with sand and intermediate on embankment removal (text p. 6, Fig. 1).  The species number was significantly lower when the embankment was removed than when sandy sediment added, but there was no difference in evenness. (text p. 6, Fig. 2)  There were clear differences in species composition between the three types of restoration measures. The cover of therophytes was higher in sites where gravel was added compared to sites where sand was added. Plots with sand addition had more moisture indicating species and target species for reed stands. No differences were found however for the amount of pioneer target species and alien species between the three types of restoration. (text p. 6-10)  Vegetation cover did not vary significantly between restored areas and continuous reed stands. However, the species number per plot as well as species diversity were higher in restored areas than in non-restored areas. Species composition also varied significantly between impact and control sites, with higher number of endangered species and target species for reed stands, as well as less alien species in restored sections compared with non-restored sections. (text p. 10) | c |
| WOS_30964 | Toth & van der Valk, 2012 | Medium | USA | Riparian vegetation | River | River floodplain | *River restoration*  Restored (dechannelization) vs channelized floodplain | Mean plant species diversity and diversity of obligate and facultative wetland  species at the Pool C did not show any other restoration-related changes.  Post-restoration weighted averages of wetland indicator species at Pool C sites were significantly lower than at the control sites in Pool A. (text p. 68-69, Fig. 5-6) | c |
| WOS_4888 | Cavaille et al., 2013 | Medium | France | Riparian vegetation | River | Riverbanks | *Bank engineering*  - Civil engineering embankments: riprap protection  - Mixed embankments: riprap at the lower  part of the bank + cuttings and woody plantation at the upper part  - Bioengineering embankments: willow fascines at the lower part of the bank | Plant species richness differed along the naturality gradient, with bioengineering or mixed techniques having significantly higher species plant diversity compared to purely civil engineering techniques. (text p. 26, table 2)  Floristic composition also differed significantly along the naturality gradient of embankment techniques. (text p. 26, Fig. 3-4)  The number of exotic species however did not differ between the three techniques. (text p. 27, Fig. 6)  However exotic species were more frequent on riverbanks protected using purely civil engineering techniques compared with mixed and bioengineering techniques. (text p. 27, Fig. 7) | d |
| WOS_4889 | Cavaille et al., 2015 | Medium | France  Switzerland | Riparian vegetation | River | Riverbanks | *Bank engineering*  - Civil engineering: riprap protection  - Mixed technique: riprap and bioengineering  - Completely bioengineering technique 1: vegetalized cribwall  - Completely bioengineering technique 2: willow fascines at  the lower part of the bank with cuttings  - natural riparian willow stands (reference) | Plant species richness varied significantly depending on the bank type, with higher richness on banks having undergone bioengineering techniques (Fascine, Cribwall and Mixed banks) than on banks with only ripraps. Significant pairwise differences were found between "Riprap" banks and "Mixed" or "Natural" banks. (text p. 69, Fig. 2, table 3)  Simpson, Shannon and Evenness indices were also significantly different between techniques, although pairwise differences were not significant. Only the Shannon index was significantly higher in "Mixed" banks compared with "Cribwall" banks. (text p. 69, see table 4 for detailed results)  Plant community assemblages also differed significantly depending on the type of riverbank protection technique. Indeed, "Mixed", "Fascine", and "Cribwall" banks were significantly different from "Natural" banks. "Riprap" banks on the other hand were significantly different from "Cribwall" and "Fascine" banks. (text p. 69, table 5)  Differences in terms of biological groups and functional diversity were also found between techniques. (text p. 70; see table 6 and 7 for detailed results) | d |
| WOS_8122 | Dufour et al., 2007 | Medium | France | Pioneer vegetation | River | Riverbanks | *Bank engineering*  Embanked reach constrained by dykes on both banks vs unconstrained reach | Vegetation sampled in quadrats within the embanked reach had a greater index of similarity than vegetation sampled in quadrats within the unconstrained reach. (text p. 1249)  Pioneer vegetation units in the embanked and unconstrained reaches had  significantly distinct vegetation communities, with a relatively high number of  vegetation species that are unique to the unconstrained reach. (text p. 1251, Fig. 4)  Species richness was significantly lower in the embanked reach compared with  the unconstrained reach. The difference in total stem density of ligneous species was not statistically significant between the types of reaches. Yet, significant differences were found for the relative densities of the three most common ligneous species, with more *Populus nigra* in the embanked reach and higher stem densities of *Salix eleagnos* and *Salix purpurea* in the unconstrained reach. (text p. 1251, table 2) | d |
| 2125 | Janssen et al., 2019 | Medium | France  Switzerland | Riparian vascular plants | River | Riverbanks | *Bank engineering*  - riprap protection  - mixed protection (riprap lower, soil bioengineering upper)  - vegetated crib wall  - willow fascines | Richness and density of pioneer tree species was significantly more important on mixed, crib wall, fascine and natural sites than on riprap sites, and richness only was significantly more important on fascine sites than on mixed sites.  Also, the density of other tree species was significantly more important on mixed sites than on riprap, fascine and natural sites. (text p. 4, Fig. A5) | d |
| 151 | Martin et al., 2020 | Medium | France | Riparian vegetation | River | Riverbanks | *Bank engineering*  - willow fascines  - ripraps  - mixed technique (lower-bank ripraps with upper-bank plantings) | The species composition of fascine banks was significantly different from that  of their downstream banks, while upstream, structure and downstream banks  hosted similar species in mixed-technique and riprap sites. (text p. 5-6, Fig. 2)  Regardless of the type of structure, no significant difference in total  richness was found between upstream, structure and downstream banks.  Riprap banks showed significantly less abundant cover of plant species than  their upstream banks, while differences in total abundance for mixed-technique  and fascine sites were not significant. (text p. 6, Fig. 3a)  In fascine sites, downstream banks hosted a higher number of invasive alien  species (IAS) than fascine banks. No significant differences were found for the  other techniques (text p.6, Fig. 3b)  Riprap banks displayed an invasive alien species (IAS) relative abundance significantly higher than their downstream banks, while fascine banks displayed a lower IAS relative abundance than both their upstream and downstream banks (note that differences where only marginally significant for absolute abundance values). (text p. 6, Fig. 3b)  No significant differences in mean herbaceous, shrub and tree covers were  found for any type of site (text p. 6, Fig. 4) | d |
| WOS_24025 | Pettifer & Kay, 2012 | Medium | UK | Riparian vegetation | River | Riversides in suburban areas | *Bank engineering*  Flood defences: with/without and older vs newer | The number of plant species present (species abundance) was significantly higher in riparian areas without ﬂood defences. No difference was found between sites with new and old defences. (text p. 346)  Simpson’s index and species richness values were higher in sites without  defences. Values were similar however between sites with new and old defences. (text p. 346, Fig. 3) | d |
| 855 | Tisserant et al., 2020 | Medium | France  Switzerland | Riparian vegetation | River | Riverbanks | *Bank engineering*  - pure soil bioengineering (willow fascines, brush layers and cuttings)  - ripraps (hard engineering)  - mixed technique (lower-bank ripraps, brushlayers and cuttings of willow species on the middle part and herbaceous seeding on the upper part) | The species pool for total, shade-tolerant and competitive groups was  significantly higher on mixed and pure sites, compared to ripraps. There were  more hygrophilous species in pure sites than in ripraps, more ruderal in mixed  sites than in ripraps, and more non-native in pure sites than in mixed sites.  (text p. 4, table 1)  Significant interactions between type of stabilization structure and time since  completion were also found for some species groups. (see table 3 and Fig. 3 for detailed results)  Plant density was significantly lower on ripraps than on pure and mixed sites  for total, ruderal and competitive species. It also decreased for hygrophilous  and increased for shade-tolerant species on pure sites, compared to ripraps.  Significant differences in species composition occurred between mixed and riprap sites, and between pure and riprap sites, but not between mixed and pure sites. (text p. 5, Fig. 5, see table 3 and Fig. 4b for detailed results with  interactions with time since completion) | d |
| 2774 | Wollny et al., 2019 | Medium | Germany | Riparian vegetation | River | Riverbanks | *Bank engineering*  Bank stabilization structures:  - ripraps  - front-fixed banks  - unfortified banks | Tree coverage was significantly higher at front-fixed banks compared to riprap  banks, while intermediate values where found at unfortified banks. There were  no differences for the shrub layer. (table 2)  The highest diversity levels were observed at ripraps, with significant  differences to front-fixed banks for Shannon diversity and evenness at the Main, and Shannon diversity and species richness at the Danube. Unfortified banks mostly tended to show slightly higher diversity levels than front-fixed  banks. (text p. 330, Fig. 4)  Except for levels of functional evenness which was highest at unfortified  banks at the Danube, analysis of functional diversity revealed non-significant  results. (text p. 330, Fig. 5) | d |
| EM_32 | Bardet, 2011 | Medium | France | Vascular plants | Road | Roadsides | *Mowing*  Mowing regimes:  - mowing annually in fall  - mowing annually in winter  - mowing biannually in fall  - mowing biannually in winter | There was no difference in species richness overall between mowing regimes in 2010 and 2011. (text p. 17)  Some differences appeared when looking at each site separately and with the 2011 survey but results were quite heterogeneous. (text p. 18)  Plant communities did not seem to differ in terms of nutrient and light preferences or in the representation of life cycles. (text p. 17-18) | e |
| ACTU_WOS_T_2720 | Bouchet et al., 2017 | Medium | France | Vascular plants | Road | Road slopes (embankments and roadcuts) | *Mowing*  Plots mown once a year in late autumn/early winter vs unmown plots | Mowing significantly explained the variation in species composition among  quadrats. (text p. 380)  Onset of flowering date was earlier with mowing, while end of flowering date  and flowering duration did not vary with mowing. (text p. 381) | e |
| WOS_777 | Ameloot et al., 2006 | Low | Belgium | *Rhinantus angustifolius*  *Rhinantus minor*  *Vascular plants* | Road | Motorway verges | *Biomass reduction using hemiparasitic plants*  Sowing of seeds of *R. angustifolius* and *R. minor* to control above-ground biomass on verges | Plots with higher number of *Rhinanthus* plants had significantly lower above-ground biomass, the effect being largely due to plots sown with *R. minor*. (text p. 180, table 4) | e |
| ACTU_WOS_T_6047 | Chaudron et al., 2016 | Medium | France | Vascular plants  Seed rain  *Brachypodium pinnatum*  *Arrhenatherum elatius*  *Dactylis glomerata*  *Schedonorus arundinaceus* | Road | Road verges  (herbaceous boundaries of arable fields) | *Mowing*  - mowing of berms in early summer  - mowing of berms in late summer  (in all cases embankments were mown once in winter and biomass was not removed from site) | The period of mowing did not influence the species richness of berm standing vegetation. (text p. 222, Fig. 2a)  However, significantly more species were found in the seed rain of the berms mown later than those mown earlier. (text p. 222, Fig. 2b)  More seeds were trapped in the seed rains of berms mown later. The same trend was not significant however for the seeds trapped on embankments. (text p. 222, Fig. 3)  The Bray-Curtis dissimilarity index between the BSV and the seed rain was significantly lower on the berm when mowing was later. The same pattern was also found for Sorensen’s similarity index between the BSV and berm seed rain. (text p. 223, Fig. 4)  Under late mowing regime, *B. pinnatum* was more abundant in both berm and embankment seed rains, while *A. elatius* was more abundant in the berm seed rain only and *D. glomerata* in the embankment seed rain. The abundance of *S. arundinaceus* was not signiﬁcantly inﬂuenced by the mowing period at any sample point either in standing vegetation or seed rain. (text p. 224, Fig. 5) | e |
| 5285 | Chaudron et al., 2020 | Medium | France | Vascular plants | Road | Road verges (herbaceous boundaries of arable fields) | *Mowing*  - standard mowing regime (one mowing in early summer, a short mowing length (5 cm) and without biomass removal)  - agro-environmental mowing regime (one late mowing between 2 and 3 months after date for standard regime, higher mowing height (25cm) and biomass removal) | There were significant compositional differences between plant communities in boundaries depending on the mowing regime but the latter explained only a small part of the variation after accounting for field identity (text p. 6).  The average weed cover was not significantly different between boundaries with different mowing regimes. (text p. 6)  There was a significantly higher average number of inflorescences in sites with an agro-environmental mowing regime. (text p. 6, Fig 3A) | e |
| WOS_5737 | Clarke & White, 2008 | Medium | Australia | Vascular plants | Powerline | Powerline corridor | *Slashing*  Vegetation cut in powerline corridor in 2000 vs reference site protected from slashing since 1994 | Significant differences were observed in vegetation community between managed and unmanaged sites. (text p. 260)  As time since management increased, vegetation structure changed significantly in both of the managed corridors, but remained stable in the reference corridor. (text p. 260, Fig. 3)  Significant temporal changes in vegetation communities were also found in both managed and reference corridors. (text p. 260-261, see table 3-4 for detailed results) | e |
| REVIEW2018_33 | Endels, 2007 | Medium | Belgium | *Primula vulgaris*  (very rare and declining species) | Road | Road grassland verges | *Mowing*  - no mowing  - mowing once a year  (mid-July)  - mowing twice a year  (mid-July and mid-  October)  (+ hay removal for all  treatments) | Reproductive output was significantly lower if plots were not mown, but no  significant differences were found between mowing once or twice a year.  (text p. 354, Fig. 1d-f)  Proportional survival differed significantly among treatments except for  vegetative adults and was lowest for all life stages other than the juvenile  category if plots were left unmown. Differences in survival for individuals  that were mown once or twice a year were generally small. (text p. 354 , Fig. 2)  Population growth rates were higher in mown sites and the difference with sites  not mown tended to be higher if mowing was done twice a year. (Fig. 3) | e |
| WOS_10494 | Gerard et al., 2008 | Medium | Belgium | Vascular plants | River | Lowland floodplain meadow | *Mowing*  annually mown sites vs non-mown sites | Mowing significantly explained part of the variation in species composition. In  terms of growth forms, mown sites were characterized by smaller graminoids, creeping herbs, rosette herbs, and less tall graminoids.  The effect of mowing on species richness interacted with flooding frequency such that mown, frequently flooded sites showed higher species richness than  non-mown, frequently flooded, or mown, infrequently flooded sites.  (text p. 1783, table 1, Fig. 2)  In terms of the C-S-R signatures of the vegetation, values for competitiveness  were lower in mown sites than in non-mown sites, while values for  stress-tolerance and ruderality were higher in mown sites. The difference in  ruderality was also greater under frequently flooded conditions.  (text p. 1784, table 1, Fig. 3) | e |
| REVIEW2018_35 | Halbritter et al. 2015 | Medium | USA | Flowering plants | Road | Highway margins | *Mowing*  - no mowing  - mowing every 3 weeks  - mowing every 6 weeks | The mowing treatment had a significant effect on each of the 4 floral resource  variables (floral species richness and density of flowers for all species and  for nectar species). (text p. 1085, table 4)  Sites under no mowing or mowing every 6-week had similar numbers of species and densities, which were significantly greater compared to the 3-week treatment. (text p. 1085, Fig. 2) | e |
| REVIEW2018_36 | Helden & Leather, 2004 | Medium | UK | Grassland plants | Road | Urban roundabouts and road enclosed sites | *Mowing*  Cutting frequency:  - no cut  - 7 days  - 14 days  - 40 days | Mowing frequency showed significant associations with the log number of  grassland plants species, but the differences between the four mowing regimes  are not reported. (text p. 373) | e |
| WOS_13910 | Ishida et al., 2008 | Medium | Japan | Riparian vegetation | River | Riverside floodplain | *Burning*  Burnt vs intact plots | Burning affected the occurrences of herbaceous annuals positively, but impacted those of woody and fern species negatively. It also influenced the occurrences of autochore species positively, and negatively the occurrence of synzoochore species. (text p. 749-750; see table 6 and 8 for associations with individual species) | e |
| WOS_14194 | Jansen & Robertson, 2001 | Medium | Australia | Riparian vegetation  *Eucalyptus camaldulensis*  *Phragmites australis* | River | Riverside floodplain | *Grazing*  Gradient of grazing intensity (as measured by cowpat densities) | High cowpat densities were associated with lower values for the index of  vegetation cover, native vegetation cover, and index for the presence of  indicative species. (Fig. 5) | e |
| ACTU_WOS_F_3404 | Kidd & Yeakley, 2015 | Medium | USA | Riparian vegetation ≤ 2m  *Phalaris arundinacea*** | River | Riparian wetlands | *Grazing*  - continuous livestock grazing  - short-term (3 years) since livestock exclusion  - long-term (13 years)  since livestock exclusion | The average species richness was significantly higher in the ‘current grazing’  (CG) site compared with the ‘short term exclusion’ (STE) and ‘long term  exclusion’ (LTE) treatments for all, native and non-native species. (table 1)  Shannon diversity index was the lowest in LTE treatment for all, native and  non-native species. It was also lower in STE compared with CG site for native  species only. (table 1)  The average cover of native species was the highest in STE site, then in CG and the lowest in LTE treatments. Conversely for non-native species, abundance was the highest in LTE, then in CG and the lowest in STE. For the abundance of the invasive *Phalaris arundinacea*, it was significantly higher in LTE site compared to the two other treatments. (table 1)  All of the sites were found to have significantly different plant communities (text p. 508) | e |
| WOS_19025 | Magadlela et al., 1995 | Medium | USA | Herbaceous and woody species | Powerline | Powerline ROW | *Grazing*  *pre-grazing treatments:*  - control  - cutting  - herbicide (2,4-D plus triclopyr)  grazing treatments: sheep vs goat grazing only or including period of mixed grazing | Brush cover decreased significantly faster on plots grazed by goats compared to sheep, although during the fifth year all treated plots showed very low amounts of total brush (text p. 3, Fig. 1)  Cutting and herbicide pre-grazing treatments were both effective in reducing brush cover before grazing and there was no difference between the two treatments. Brush was cleared more rapidly with cutting or herbicide followed by goats vs. those treatments followed by sheep. (text p. 3-4, Fig. 2-3) | e |
| WOS_20670 | Milberg & Lamont, 1995 | Low | Australia | Vascular plants | Road | Roadsides | *Burning*  Before vs after burning and unburnt sites | Generally, the weed species had increased relatively more in cover and  frequency 30 months after the fires than had the native species. (text p. 47, table 2-3)  In burnt plots, number of weed species, number of individuals and the cover of  weed were significantly greater than in unburnt control plots. (text p. 47) | e |
| WOS_20030 | McDonald, 2011 | Medium | UK | Vascular plants | River | Old field floodplain | *Grazing*  - ungrazed  - grazing by sheep  - grazing by cows | Significant differences in species composition where observed between the  treatments in the years 1994, 1995, 1997-1999. (text p. 171, Fig. 3b) | e |
| ACTU_WOS_F_1875 | Moinardeau et al., 2016 | Medium | France | Vascular plants | River | River embankments | *Grazing and mowing and slashing*  Site 1:  - cattle grazed vs mown grasslands  Site 2: horse grazing vs no grazing (in grasslands with three categories of vegetation height)  Site 3:  - no treatment  - cleared and grazed by goats  - cleared and fenced  - grazed but not cleared | On site 1, Bray–Curtis indices were significantly higher in cattle-grazed grasslands than in mown grasslands. (text p. 989, Fig. 3)  On site 2, Bray–Curtis index in 2015 was significantly higher in the open grasslands and ST compared to all the other plots. (text p. 990, Fig. 6)  On site 3, Bray-Curtis indices were significantly higher in 2014 and 2015 in cleared and grazed as well as cleared and fenced plots than in the control. (text p. 992, Fig. 10)  There was no difference in species richness between grazed and mown grasslands on site 1. (text p. 989)  On site 2, species-richness was significantly higher in 2014 in the open and short grasslands than in the other plots, with the exception of the tall grasslands dotted with shrubs. (text p. 990)  No difference in species richness between treatments were found on site 3 in 2014, but the cleared and fenced treatment had a higher species richness than the control in 2015. (text p. 992, Fig. 9) | e |
| REVIEW2018_45 | Noordijk et al., 2009 | Medium | Netherlands | Flowering plants | Road | Highway verges | *Mowing*  - no management  - mowing once in early autumn  - mowing once in early autumn + hay removal  - mowing in early summer and early autumn  - mowing in early summer and early autumn + hay removal | Mowing regime had a significant effect on flower diversity and abundance, with the highest diversity and abundance found where mowing occurred twice with hay removal, whereas plots where no management occurred had the lowest values. (text p. 2098, Fig. 1a-b) | e |
| REVIEW2018_46 | Noordijk et al., 2010 | Medium | Netherlands | Vascular plants | Road | Highway verges | *Mowing*  - no management  - mowing once in early autumn  - mowing once in early autumn + hay removal  - mowing in early summer and early autumn  - mowing in early summer and early autumn + hay removal | For the medium and high productive verges, when comparing the two extreme treatments (no management vs mowing twice a year with hay removal), plant  species richness, flowering plant species richness and flower abundance  increased while standing biomass decreased. The other treatments took  intermediate positions, in orders varying between the different vegetation  characteristics. However, there was no clear pattern between the different  treatments for low productive verges. (text p. 743, see Appendix C for details) | e |
| REVIEW2018_39 | Parr & Way, 1988 | Medium | UK | Vascular plants | Road | Road verges | *Mowing*  Unmanaged control plots and combinations of cutting date (single cuts in June or July), cutting frequency (0, 1, 2, or 5 times per annum), cutting machine (haymower, flail mower or rotary mower) and leaving or removing cuttings  (see table 1) | The type of mowing machine used to cut the plots and delaying a single cut from June to July had no significant effect on species richness at either site.  (text p. 1076)  Results for species richness suggest that cutting frequency had a significant  impact (although they are based on very few data points). Total species richness per quadrat at both sites was lowest in the uncut controls and was greatest with two cuts per annum. The same pattern was found for the number of grass species. The trend was not significant however for herbs, while shrubs declined as cutting frequency increased. The same trends where found for species richness per plot, but only the decline of shrubs with cutting frequency was significant. (text p. 1076, Fig. 1)  Hay removal was associated with a significant increase of total and herbs  species richness per quadrat. No effect where found however with species  richness per plot. (text p. 1079, table 3) | e |
| WOS_23574 | Simoes et al., 2013 | Medium | Portugal | Vascular plants  *Dittrichia viscosa* | Road | Road inside verges | *Mowing*  - unmown sites  - mowing one a year  - mowing twice a year | *Dittrichia viscosa* shrub density was higher in road verges mown once a year  compared to twice a year. (table 1)  Numbers of species per m2 were higher in roads mown twice a year than in roads mown once a year. The impact of mowing regime on species diversity interacted with the presence/absence of *D. viscosa* on road verges, since for *Dittrichia* verges, Shannon diversity index was higher in roads mown once a year, but for non-*Dittrichia* verges it was higher in roads mown twice a year. Evenness index was higher in roads mown once a year. (text p. 245, table 2)  Dissimilarities were found in communities of non-Dittrichia verges between  roads with different mowing regime, but not for roads were *Dittrichia* was  present. (text p. 246)  Production of flower heads was lower in mown sites in autumn 2008 regardless of mowing frequency, while in autumn 2009 it was actually higher in roads mown once a year and lower in roads mown twice a year compared to unmown sites. A similar pattern was found for the amount of seeds produced per square meter (text p. 248, table 5) | e |
| WOS_24337 | Plieninger, 2007 | Medium | Spain | *Quercus ilex* | Road | Abandoned dehesas at roadsides | *Grazing*  Gradient of grazing pressure (as measured by fecal counts, juvenile browsing and sapling browsing) | Three of the four indicators used to measure grazing pressure were found to be  internally consistent (fecal counts, juvenile browsing index and sapling browsing index), however these indicators were not significantly associated with juvenile and sapling densities. (text p. 4) | e |
| WOS_25188 | Ranganath et al., 2009 | Medium | USA | Riparian vegetation | River | River banks | *Grazing*  Streams with vs without livestock access | The median amount of ground cover vegetation was significantly greater in the  livestock exclusion reaches than the grazed reaches. (text p. 37, Fig. 2, table 4) | e |
| WOS_25940 | Robertson & Rowling, 2000 | Medium | Australia | Riparian vegetation  *Eucalyptus spp.* | River | Stream banks | *Grazing*  Banks with or without livestock access | The mean density of seedlings and saplings of *Eucalyptus spp.* was significantly greater in areas with no livestock access. (text p. 532)  Significant differences in species composition were also found between areas  with or without livestock access. There were no differences with regards to  time since exclusion however for the mainstream sites. (text p. 532-533) | e |
| WOS_27266 | Schaich et al., 2010 | Medium | Luxembourg | Riparian vegetation | River | River floodplain pastures | *Grazing*  Grazed by Galloway cattle vs ungrazed plots sampled after a river restoration project | Mean species number per plot significantly increased between 2004 and 2006.  Except for evenness, all other indicators of vegetation diversity, as well as  rare, and typical wetland species significantly increased between 2004 and 2006 (text p. 145, Fig. 1)  There was also a significant short-term variation in overall vegetation  composition over the period, with differences between riparian, floodplain and  valley edge plots (text p. 135, table 4)  Mean species richness was significantly higher in grazed than ungrazed plots in  2006 and 2007 (but not in 2005). The same pattern was found for Shannon  diversity index, while evenness did not differ between treatments in any  years. There was no clear difference between grazing treatments regarding the number of rare species. (text p. 147, table 7)  Turnover rates between 2005-2006 and 2006-2007 were higher in the ungrazed than in the grazed treatment. (text p. 147) | e |
| WOS_29505 | Stockan et al., 2012 | Medium | UK | Riparian vegetation | River | Riparian margins | *Grazing*  - unbuffered sites: no permanent fencing to prevent livestock accessing the watercourse  - buffered sites: fenced off strips of at least 2m width  - reference sites: mature, woody riparian vegetation | Shannon and Simpson diversity indices, total and riparian richness did not differ significantly between buffered, unbuffered and reference sites. Although  buffering seemed to have a significant effect on plant species richness within  the Tarland catchment (but not within the Ugie catchment) because richness declined with the age of the buffer strip. (text p. 368, Fig. 1)  Riparian abundance was higher in unbuffered sites compared to buffered and  reference sites. The number of trees, tree species richness and canopy cover were highest in the reference sites and intermediate in buffered sites. Ellenberg values were found to be significantly lower in reference sites for 'light' and 'reaction'. (text p. 368, table 3) | e |
| WOS_31642 | Van Looy & Meire, 2009 | Medium | Belgium | Riparian vegetation | River | Dry river grasslands | *Grazing*  Management of river meadows:  - with haying and/or pastures  - natural grazing  - no management | Species composition showed significant covariance with management type.  (text p. 39, table 3) | e |
| WOS_32121 | Vogt et al., 2007 | Medium | Germany | Riparian vegetation | River | Fen grasslands | *Grazing*  Grazed vs abandoned grasslands | Grazing had a significant impact on the total number of species (higher in  grazed sites) but not on the total number of seedlings (see also table 1 for  individual results for the most frequent species). (Fig. 2, table 1)  The species composition of both seedlings and established vegetation also  varied significantly with exposure to grazing. (table 2) | e |
| 17481 | Wigginton & Meyerson, 2018 | Medium | USA | Vascular plants | Road | Roadsides | *Mowing*  Mowing regime:  - mowing 3-6 times a year (‘mowed’)  - mowing 1-2 times a year only in operational zone (‘restored’)  - no mowing (‘reference’) | There were no differences between mowing regimes in species diversity and for total, introduced and invasive species richness. The reference sites however tended to have higher native species richness than mowed and restored sites (the difference was not significant only at the 10m2 scale and at 1m2 scale with restored sites). (text p. 46, Figure 3)  There were no differences between mowing regimes for native and invasive species cover. The cover of introduced species in reference sites on the other hand was significantly lower than in mowed and restored sites. (text p. 47, Figure 4-5) | e |
| WOS_2597 | Bentivegna & Smeda, 2008 | Medium | USA | *Dipsacus laciniatus*** | Road | Highway verges | *Alien plant management*  Herbicide treatments:  - control  - Glyphosate  - 2,4-D  - 2,4-D + triclopyr  - 2,4-D + picloram  - Dicamba + diflufenzopyr  - 2,4-D+ clopyralid  - Metsulfuron-methyl  - Sulfosulfuron  - Paraquat  - Imazapyr  - Sulfometuron methyl | Overall, a number of herbicides were effective in managing emerged plants but  re-infestations of treated sites are likely. (text p. 502)  Visual estimates of injury to the invasive cut-leaved teasel varied between  herbicide treatments and with location and time of evaluation.  (see table 3-4, Fig. 1 for detailed results for the numerous treatments)  Cut-leaved teasel emergence following herbicide applications varied among  treatments and between locations and for many of the selected treatments,  cumulative emergence was similar to or greater compared to the control plots.  (see text p. 505 and Fig. 1 for details) | f |
| WOS_3047 | Blanchard & Holmes, 2008 | Medium | South Africa | Riparian vegetation | River | Riparian zones in Fynbos Biome | *Alien plant management*  Clearing treatments (alien trees):  - fell only  - fell and remove  - fell and burn  vs non-invaded reference plots | At the 50m² scale, 'Fell and Remove' plots had significantly greater species  richness than 'Fell Only', 'Fell and Burn' and reference plots.  At the 1m² scale, richness values were more similar but reference plots were  significantly richer than 'Fell Only' and 'Fell & Burn' treatments. 'Fell &  Remove' plots had the second highest value which was significantly higher  than that of 'Fell Only' plots. (text p. 426, table 2)  Diversity measures differed significantly among treatments for both Shannon and Evenness indices. The 'Fell & Remove' treatment recorded the highest diversity and was significantly different to 'Fell Only' and 'Fell &  Burn', but not to the reference plots. The 'Fell Only' treatment was the only  treatment to have significantly lower diversity than the reference condition.  The reference plots had significantly higher evenness scores than 'Fell Only'  and 'Fell & Burn' plots, but not compared to 'Fell & Remove' plots.  Canopy cover also differed significantly between treatments, with the highest  values found on reference sites and the lowest in 'Fell Only' plots. 'Fell &  Remove plots also had a significantly higher indigenous cover than 'Fell  Only' plots. (text p. 426-427, table 2)  The different treatments had varying proportions of growth forms that were significantly different from the reference plots. (text p. 427, see Fig. 4 for details)  Cover of woody alien species in reference and 'Fell & Burn' plots were lower  than in 'Fell Only' and 'Fell & Remove' plots. The cover of alien herbaceous  species on the other hand was highest in 'Fell & Remove' and reference plots  than in 'Fell & Burn'. (table 2) | f |
| WOS_4157 | Bunn et al. 1998 | Low | Australia | Brachiaria mutica** | River | Riversides | *Alien plant management*  50% or 90% shading with a cloth vs no shading over three-month period | After three months of shading, para grass biomass and height were significantly  lower in the 50% and 90% shade treatments compared with the open control, with more important reductions in the 90% shade treatment.  (text p. 175, table 2-3, Fig. 2-3) | f |
| WOS_4387 | Caffrey, 2001 | Medium | Ireland | *Heracleum mantegazzianum*** | River | Riverbanks | *Alien plant management*  Glyphosate treatment +  destruction of flowering  plants and burning  vs no treatment | Weed control program led to a high reduction in the density of the invasive  giant hogweed plants by the end of the study period both for immature and adult plants for all treated sites compared to the control. (text p. 31, Fig. 2-3) | f |
| WOS_5698 | Claeson & Bisson, 2013 | Medium | USA | Riparian vegetation  *Polygonum sp.*** | River | Riparian areas of low terrace and floodplain forests | *Alien plant management*  Sites treated (3 to 6 years prior to study) with various herbicide mixtures and application methods against invasive *Polygonum sp.* vs non-invaded reference sites | Differences in native species richness and tree cover between Knotweed treated  sites and reference sites were not significant (although significant  interaction with site were found). (text p. 213-214, table 2)  Exotic species richness, exotic shrub and forb cover, and knotweed cover were  all significantly higher in treated sites. Conversely, native forb and shrub  covers were significantly higher in reference sites. (table 2) | f |
| WOS_5851 | Cockel et al., 2014 | Medium | England | Riparian vegetation  *Impatiens glandulifera*** | River | Riverbanks | *Alien plant management*  For sites with high, medium or low invasion levels by *Impatiens glandulifera*:  - pruning at 6-week intervals  - weeding at 6-week intervals  - no removal | At the most heavily invaded sites, there was a significant decrease in the  percentage cover of *Impatiens glandulifera* in the treated plots in comparison  with the control plots and a significant increase in the cover and number of  other species. (text p. 221, Fig. 2-4, table 3)  In sites with moderate invasion levels, the percentage cover of *I. Glandulifera* showed significant differences with treatment but not the cover and number of other species. No significant differences were found for lightly invaded sites.  (text p. 221, table 3; see also table 4 for complementary analysis of midsummer data) | f |
| WOS_10093 | Galatowitsch & Richardson, 2005 | Low | South Africa | Riparian woody species | River | Riverbanks | *Alien plant management*  Invasion/clearing gradient  of invasive trees (especially *Acacia longifolia* and *Acacia mearnsii*) | The density of invasion/removal significantly affected riparian species  composition with a post-clearing shift from canopies dominated by indigenous  large and small tree species to those dominated by shrubs or re-invading alien  trees (see table 3 for association with individual species occurrence). (text  p. 515-516, table 3, Fig. 2)  Species richness was lower in plots with greater invasion/clearing. (text p. 516)  Indigenous tree regeneration was lower in areas with greater alien invasion/clearing. (text p. 516) | f |
| WOS_13580 | Hulme & Bremner, 2006 | Medium | UK | Herbaceous riparian vegetation | River | Riverbanks | *Alien plant management*  Plots with removal of the invasive *impatiens glandulifera* vs plots without removal | The presence of *Impatiens glandulifera* significantly reduced both the mean  abundance of species within plots and their frequency between plots.  (text p. 45)  Removal of *I. glandulifera* led to a significant increase in species  richness, resulting in an average of four additional species per plot.  (text p. 45)  The additional species resulted in a significant increase in α diversity but a  decline in α evenness in removal plots. Similarly, where *Impatiens* *glandulifera* was removed, α richness and diversity of other non-native species increased significantly but their evenness declined. For native species the same pattern was obtained except that no difference in evenness was found in plots with or without *I. glandulifera*. (text p. 46)  Removal plots also had a significantly higher proportion of non-native species  than those where *Impatiens* was present. (text p. 46) | f |
| 1766 | Mangachena & Geerts, 2019 | Medium | South Africa | Riparian vegetation | River | Riversides | *Alien plant management*  Sites cleared of invasive tree *Eucalyptus camaldulensis* vs invaded sites vs near-pristine riparian vegetation | Native species richness was similar in cleared and near-pristine sites but  significantly lower in cleared sites. Alien species richness on the other hand  was higher in cleared sites, intermediate in invaded sites and lowest in  near-pristine sites. (text p. 4, Fig. 2a)  Native species richness did not vary significantly in sites with different post  clearing ages, while significant differences in alien species richness were  found but with no general trend. (text p. 4, Fig. 2b)  Plant composition was similar between cleared and near-pristine sites but  significantly different with invaded sites for both categories. (text p. 4, Fig. 4) | f |
| WOS_26600 | Ruwanza et al., 2013 | Medium | South Africa | Riparian vegetation | River | Riversides | *Alien plant management*  Clearing of the invasive tree *Eucalyptus camaldulensis:*  - complete clearing  - partial clearing  - invaded and uninvaded ‘natural’ control sites | Cover of all natives was lowest in invaded site, intermediate in thinned and  completely cleared sites and highest in natural sites.  Regarding all alien species, cover was highest in invaded sites and lowest in  natural sites. In thinned sites it was significantly lower than in invaded  sites but still higher compared with natural sites. Alien cover in completely  cleared sites was intermediate and not significantly different from the values  in invaded sites and thinned sites. (table 3; see also table 3 and Fig. 4 for results for specific life forms)  Overall species richness was significantly higher in thinned and completely  cleared sites than in both invaded and natural sites. The same pattern was  observed for alien richness. The richness of native species was significantly  lower in invaded sites but similar across the other treatments.  Simpson and Shannon diversity indices were also higher in sites with alien  removal, the difference being significant with both invaded and natural sites  for thinned sites and only invaded sites for completely cleared sites.  Evenness on the other hand was significantly higher in thinned sites compared  with completely cleared sites and intermediate in invaded and natural sites.  (table 4; see also table 4 for results for specific life forms) | f |
| WOS_26912 | Samples et al., 1987 | Medium | USA | *Andropogon saccharoid***  *Cynodon dactyl* | Road | Highway right-of-ways | *Alien plant management*  Herbicide application for control of the invasive grass *Andropogon saccharoid* | Glyphosate at 1.1 and 1.7 kg/ha satisfactorily controlled the silver beardgrass  with little or no permanent damage to the existing bermudagrass (text p. 123;  see table 3, 4 for detailed results) | f |
| ACTU_WOS_F_2727 | Toth, 2016 | Medium | USA | Forbs and grasses  *Hemarthria* *altissima***  *Polygonum punctatum*  *Panicum hemitomon*  *Luziola fluitans* | River | Wet prairie and broadleaf marsh plots along river | *Alien plant management*  - impact of presence of exotic grass *Hemarthria altissima* on restoration project  - herbicide treatment (herbicide glyphosate at a rate of 4.7 kg active ingredient/ha) against exotic grass *Hemarthria* *altissima* | *Impact of cover of H. altissima on restoration*  Cover of the indicator wet prairie taxa (*L. fluitans*, *P. hemitomon* and *P.*  *punctatum*) was statistically independent of *H. altissima* cover. However,  species turnover rates declined significantly with increasing *H. altissima*  cover. (text p. 65)  *Herbicide treatments on restoration*  Mean post-treatment temporal turnover rates of plant species were significantly  higher than pre-treatment turnover rates in the seven plots with established  (>25 %) cover of *H. altissima*. (text p. 69) | f |
| WOS_31390 | Urgenson et al., 2014 | Low | USA | Herbaceous and woody species | River | Highly invaded riparian forest | *Alien plant management*  Plots before and after removal of the invasive *Polygonum x bohemicum* with a glyphosate-based herbicide including no removal control plots | The removal of the invasive Bohemian knotweed led to a significant increase in  the density of both coniferous and broadleaved tree seedlings; the cover of  native shrubs, native forbs and non-native forbs, and graminoids; and the  species richness of native and non-native forbs. (Fig. 2) | f |
| WOS_3160 | Bochet et al., 2010 | Medium | Spain | Vascular plants | Road | Roadslopes | *Revegetation of verges*  - topsoiling  - topsoiling + hydroseeding with commercial seed mixture  - topsoiling + hydroseeding with native seed mixture | Total vegetation cover was higher in the topsoiling plots than in the control ones with the highest difference occurring in June 2004 after the first growing season. Total vegetation cover was higher in topsoiling + hydroseeding with a commercial mixture plots than in topsoiling only plots, but only for some years  (2004 and 2005). When hydroseeding was done with selected native species however, the positive effect on cover hold for the whole period. The difference in vegetation cover between hydroseeding with a commercial or a selected seed mixture was not significant through the study period.  (text p. 117, Fig. 6A) | g |
| WOS_3931 | Brown & Gorres, 2011 | Medium | USA | Turfgrass species | Road | Roadsides | *Revegetation of verges*  - unamended soil  - soil amended with 50% biosolids by volume  - soil amended with 50% composted yard waste by volume | Turf cover was lower in the plain soil plots than in the amended plots throughout the study. (text p. 1406, Fig. 1)  The grass was also significantly taller in the biosolids plots than in the  compost or plain soil plots for all species except idaho bentgrass and tufted  hairgrass. (text p. 1407, Fig. 2) | g |
| 10515 | Ferreiro et al., 2020 | Medium | Argentina | Vascular plants  *Trifolium repens** (seeded)  *Poa domingensis**  (seeded) | Road | Roadsides affected by tephra deposition | *Revegetation of verges*  - nonseeded control  - nonseeded biosolids compost  - nonseeded municipal compost  - seeded control  - seeded biosolids compost  - seeded municipal compost | No effect of seeding was found for any measured variables and no interaction  between compost and seeding was observed. (text p. 4, Fig. 2)  Compost application led to a significant increase in plant cover and biomass  with respect to the control plots. No differences between both composts were  observed for biomass, while cover was higher in biosolids compost than  municipal compost. (text p. 4, Fig. 2-3) | g |
| WOS_10267 | Garcia-Palacios et al., 2010 | Medium | Spain | Vascular plants | Road | Motorway roadside slopes | *Revegetation of verges*  Seeding:  - control  - hydroseeding  - hydroseeding + mulch  Fertilization:  - control  - fertilized  Irrigation:  - control  - irrigated  (full factorial design seeding x fertilization x irrigation) | Treatment effects varied between years and sites and interacted with each  other, which makes the interpretation of the patterns obtained more difficult.  Plant cover showed significant positive associations with hydroseeding but in some sites in 2007 and others in 2008, and the effect disappeared when fertilization was also applied. Irrigation also increased plant cover in 2008.  Plant diversity increased in some of the sites in 2007 but the effect was again  reduced with fertilization. Significant interactions with site and irrigation  levels were also found in 2007 and 2008.  Community composition also showed significant associations with hydroseeding that depended on the year and sites considered and sometimes also on irrigation and fertilization levels.  (see text p. 1293-4 for all interactions) | g |
| 1140 | Londe et al., 2020 | Low | Brazil | Tree species  Seed rain | River | Riparian forest | *Revegetation of verges*  Active forest restoration: saplings of about 35 tree species (mostly native) planted (10 and 20 years restoration sites) vs conserved forest | The differences in species richness of trees, number of individuals and  proportion of non-pioneer, zoochoric and melittophilic species between the  restored area and the reference ecosystem were not significant. There were  significant differences however in the number of species and number of  individuals in the seed rain and in the regenerating stratum between certain  restored sites and the reference ecosystem. The proportion of non-pioneer in  the seed bank, and non-pioneer and zoochoric species in the regenerating  stratum also varied significantly between some restored sites and the reference  ecosystem. (see table 2 for detailed results) | g |
| WOS_19746 | Matesanz et al., 2006 | Medium | Spain | Vascular plants | Road | Motorway slopes (roadcuts and embankments) | *Revegetation of verges*  Hydroseeding vs no hydroseeding | There were no significant differences in plant cover, species richness, and  aboveground biomass between hydroseeded and nonhydroseeded plots on embankments throughout the study. (text p. 301, Fig. 4) | g |
| WOS_19975 | McClain et al., 2011 | Medium | USA | Understory species | River | Riparian forest | *Revegetation of verges*  - older restored sites by planting woody species  - younger restored sites some with and some without understory species planted in addition to woody species  - remnant forest fragments (reference)  (restoration projects also included disking, planting, mowing, irrigation,  weed control, and monitoring) | At older sites restored with overstorey plantings only, in 2001 restored sites had a higher cover, frequency and richness of exotic and lower cover, frequency and richness of native species compared with reference sites. The patterns obtained in 2007 were the same except that native richness had significantly increased compared with 2001 levels. Overstorey cover was lowest in 2001, intermediate in 2007 and highest in reference sites.  (table 2)  Understory composition in terms of relative cover was significantly different  between the 2001 and 2007 surveys of the same restoration sites, and in both  years restoration sites were different from reference forests.  (text p. 284, see table 3 for relationships of the 25 most abundant species)  Concerning younger restoration sites, relative native cover, native frequency  and native richness were all lower in restored sites with no understory  species or with low intensity understory plantings than in reference sites. For  restoration sites with high intensity understory plantings however the  difference with reference sites was significant only for relative native cover  without *Galium aparine* (the most abundant native understory species).  (table 4)  Cover and frequency of native grasses and the native shrub *Rubus ursinus* were  similar in planted and reference sites but lower in not planted sites. Cover  and frequency of the native graminoid *Carex barbarae* was significantly lower in both planted and unplanted sites compared with reference sites, whereas the  difference was significant only for unplanted sites for the native forb  *Artemisia douglasiana*. (table 5) | g |
| WOS_23958 | Petersen et al., 2004 | Medium | USA | Vascular plants  *Elymus trachycaulus*  *(seeded)* | Road | Disturbed roadsides | *Revegetation of verges*  Seeding:  - seed mixture including commercial seeds of *Elymus trachycaulus*  - seed mixture including indigenous seeds of *Elymus trachycaulus*  Fertilization:  - moderate rate  - high rate  - unfertilized (seeded only)  Effect of microsite on revegetation:  - log and stone microsite  - open microsite  Erosion control treatments:  - nylon-netted excelsior cloth  - unnetted excelsior cloth  - crimped straw mulch  - hydroseed-woodchip mulched tackifier  - control (seeded only)  Transplants of native shrubs *Arctostaphylos*  *patula* and *Symphoricarpos oreophilus:* soil type x fertilization x water-absorption gel | *Fertilizer treatments*  Total grass density during the second growing season was greater in fertilized  than unfertilized plots, with no significant differences between low and high  fertilizer rates. Four years after treatment, only plots fertilized with the  high fertilizer rate had greater total grass density than unfertilized plots.  Total grass cover was similar for all treatments during each year except in  1996 when both fertilizer rates produced greater cover than unfertilized plots.  (text p. 252, Fig. 2)  Response of density and cover to fertilizer treatments varied among the four  seeded species and between years (see text p. 252-253 for details).  There were no differences in density and cover of unseeded forbs, shrubs, and  conifers among fertilizer treatments. (text p. 253, Fig. 3)  *Indigeneous vs commercial seed sources of E. trachycaulus*  Near the end of the first growing season the density of *E. trachycaulus* was  greater for the commercial than indigenous seed source but cover was similar.  By the second growing season, however, density and cover of plants from the local source were greater than the commercial source. Differences were greatest for both density and cover by the fall of the fourth growing season.  (text p. 253, Fig. 3)  *Effects of microsites*  Density of combined seeded grasses and unseeded conifer saplings was higher  both in 1995 and 1996 in microsites adjacent to stones than in nearby open  microsites. This was also true for the density of each of the four seeded grass  species taken individually. (Fig. 5-6)  *Erosion control methods*  At the end of the second growing season, plots treated with the unnetted  excelsior cloth had a higher mean total grass density than plots treated with  crimped straw, hydroseed, or raked seed only, while there were no differences  found in total grass cover for all treatments tested.  By the end of the fourth growing season plots treated with the netted excelsior  cloth had higher total grass densities than either the crimped straw or the  seeded only plots. The unnetted excelsior plots, however, only had higher total  grass density than the seed only plots. Total grass cover was similar between  treatments except for cover in unnetted plots, which was greater than cover in  crimped straw plots.  (text p. 255, Fig. 7)  *Transplant experiment*  Shrub transplants showed minimal differential response to fertilizers,  water-absorbing gels, and soil type. (text p. 248, 255) | g |
| WOS_33680 | Wu et al., 2012 | Medium | China | Vascular plants | Road | Highway slopes | *Revegetation of verges*  Spray seeding of a mixture of plants using different sowing densities for each species | Slopes I, II, and III displayed no significant differences between the survival  rates, while a significant difference was obtained between the survival rates  of woody plants of slopes I and IV (see table 2 for the sowing densities for  each slope). (text p. 1195, Fig. 1) | g |
| 806 | Yuan et al., 2020 | Medium | China | Riparian herbaceous species | River | Riversides | *Revegetation of verges*  Ecological planting of strips of semi-natural meadows (either tall-grass meadow or forb-rich meadow) vs untreated vegetation | Number of species, Shannon index and Pielou evenness index in the quadrats  of tall-grass meadow and forb-rich meadow communities were significantly higher than those in the control group. (text p. 55, Fig. 10) | g |
| WOS_34604 | Zelnik et al., 2010 | Medium | Slovenia | Vascular plants | Road | Motorway slopes | *Revegetation of verges*  Revegetation with two different seed mixtures (see table 1): one "stress  tolerators" mixture and one "nurse species" mixture | There were no differences in species richness, species diversity index or  vegetation cover between the seed mixture treatments. (text p. 453-454, Fig. 4-6) | g |
| WOS_7080 | De la Riva et al., 2011 | Medium | Spain | Vascular plants | Road | Highway slopes (roadcuts and embankments) | *Revegetation of verges*  Hydroseeding of roadcuts or embankments with a standard seed mix, with a  native seed mix or no hydroseeding | None of the hydroseeding treatments induced significant differences in terms  of local colonization and local extinction rates with respect to the untreated  plots, even when only the 15 hydroseeded species were considered. (text p. 298) | g |
| WOS_3740 | Breton et al., 2014 | Medium | France | *Populus nigra*  *Salix alba*  *Salix atrocinerea*  *Salix fragilis*  *Salix purpurea*  *Salix triandra*  *Salix viminalis* | River | Riverbanks degraded by the invasive rodent *Myocastor coypus* | *Revegetation of verges (and protection against invasive rodent)*  - fencing vs no fencing  - planting of cuttings of either 40cm or 80 cm | Survival rates were significantly greater in the inside exclosures compared with the control plots and with 80-cm compared with 40-cm cuttings.  (text p. 1006, Fig. 3, table 1) | g |
| WOS_15811 | Kiviniemi & Eriksson, 1999 | Medium | Sweden | Grassland species (17, see table 1) | Road | Road verges | *Revegetation of verges*  *-* seeds sown in plot where vegetation was removed and soil laid bare  - seeds sown in undisturbed vegetation | Seedling emergence was greater in gaps free from vegetation than in vegetated plots. (text p. 246, see table 3 for individual results)  Seedling survivorship was not generally higher in disturbed plots compared with undisturbed ones. Significant differences between the two treatments were found only for P*rimula veris* and *Plantago media*, with survival significantly greater in vegetated plots. (text p. 246, see table 4 for individual results)  Recruitment was significantly greater in disturbed plots in 12 of the 15 species where juveniles were recorded. (text p. 247, see table 5 for individual results) | g |
| ACTU_WOS_F_1638 | Flores & Osses, 2017 | Medium | Chile | Riparian vegetation | River | Riparian forest | *Vegetation replacement*  *Pinus radiata* plantation (with replacement sites) vs native vegetation (without replacement sites) | Shannon index of diversity and species richness for tree species was  significantly higher in the watersheds without replacement, while there was a  greater diversity of herbaceous species in the watersheds with replacement. No  significant differences were found between the two types of watersheds in the  diversity of shrub and climber species.  The same pattern between watershed with and without replacement was found for the species richness of trees, herbaceous, shrubs and climber species respectively.  (text p. 69, Fig. 2)  Exotic species were more diverse and in greater numbers in watersheds with  replacement, while there was a significantly higher diversity (but not  richness) of native species in watersheds without replacement. (text p. 69, Fig. 3)  There were no significant differences between watersheds with and without  replacement in plant cover at the stratum <1 m, while at the stratum 1–5 m,  there was significantly more cover in watersheds with replacement. In contrast,  cover in the stratum >5 m was significantly greater in watersheds without  replacement. (text p. 69, Figure 6). | h |
| WOS_13261 | Hosseini et al., 2011 | Medium | Iran | Woody and herbaceous species | Road | Forest roadsides | *Earth work*  Roadside clearing with/without earth work | Clearing size and presence/absence of earth work had no association with  coverage of individual woody and non-woody plants (except for *Diospyrus lotus* and *Carex sylvatica* respectively). Total coverage however was significantly different between clearings with or without earth works. (table 1-2)  Simpson index was higher in clearings of 10-15 m wide on cut slopes compared with clearings of the same size on filled slopes. Simpson index however was similar between clearings without earth works. (Fig. 4) | h |
| WOS_14251 | Jarzyna et al., 2010 | Medium | Poland | *Atriplex tatarica*** | Road | Urban roadsides | *Salinity due to road maintenance*  Gradient of salinity as a result of winter road maintenance | A higher salinity in roadside verges was associated with a significant increase  in cover of the invasive *Atriplex tatarica*. (text p. 251, table 1) | h |
| EM_282_10 | Le Viol, 2009 | Medium | France | Vascular plants | Road | Highway roadcuts | *Planting of hedgerows*  Sites with planted hedgerows vs sites without hedgerows | Planted hedgerows had a significant positive effect on plant richness at the  local scale but not at the site level. (text p. 284)  Sites with and without planted hedgerows supported significantly different  plant communities both in terms of presence/absence data and of species  attributes. (text p. 284) | h |
| 15249 | Mori et al., 2018 | Medium | Italy | *Photinia × fraseri Viburnum lucidum* | Road | Roadsides | *Planting of vegetation barrier*  Either *Photinia × fraseri* or *Viburnum lucidum* at 0.5 or 1 plant m−2 planted 2m away from the road | The vegetation barrier had a significant impact on the deposition of most  fractions of particulate matter (PM) with distance from the road. Indeed,  roadsides with planted trees showed an increase in deposition between 2m and 12.5m from the road followed by a reduction of PM deposition at 19.5m, while no reduction was observed for the lawn strip.  (text p. 731, Figure 3C-D)  Barrier and lawn strips differed in the deposition of 8 out of 16 elements (Ca,  Al, K, Mg, Mn, Ni, Mo, Co), with higher values in the barrier strip compared  with the lawn strip. (text p. 731, Figure 4)  The planting density of *P. x fraseri* was associated with an increase of some PM fractions while no effect of planting density was found for *V. lucidum*.  (text p. 730, Fig. 2E-F)  In *P. x fraseri*, leaf deposition of some PM fractions were lower compared with  *V. lucidum*. (text p. 730, Fig. 2A) | h |
| WOS_26300 | Rose & Webb, 1994 | Low | UK | Vascular plants | Pipeline | Heathland vegetation above pipeline | *Vegetation cuts and temporary ballast addition during pipeline building*  Effects of temporary roadways associated with pipeline building on vegetation regeneration:  vegetation cut +/- ballast for various durations (2, 4, 12 weeks) and  treatment applied at different time of the year (April, June, September)  (see table 1) | The vegetation cover in dry heath sites showed some significant differences  depending on the time of the year when cutting was done and whether ballast was added and for how long. Ballast-addition treatments and plots  cut later in the year (September) had the lowest covers. (see table 4 for  all comparisons between the various treatments)  Similar results were obtained for wet heath sites. (detailed in table 5)  Significant changes in occurrence were observed for a few species between 1986 and 1988 in dry heath sites on the cut/cut-and-rolled plots and the  cut-with-ballast-addition plots. For both treatment categories, *Polygola*  *serpylliflora*, *Zygogonium ericetorum*, *Campylopus brevipilus* and *Agrostis*  *curtisii* were more frequent, whereas *Hypnum cupressiforme* occurred less often in 1988. On the cut-with-ballast-addition *Erica cinerea* also decreased in  abundance. No significant changes were observed however on plots with only  ballast addition. (see table 6 for all changes in occurrence and table 7 for similar results for wet heath sites)  All treatments had a greater number of dead rootstocks than from controls, with the largest difference linked to the duration of ballast cover. The effect of  cutting was not significant however for wet heath sites. (text p. 648)  Both cutting and covering vegetation with ballast significantly increased  seedling establishment, cutting having the largest effect. (table 8) | h |
| ACTU_WOS_T_1209 | Chaudron et al., 2018 | Medium | France | Vascular plants | Road | Road verges  (herbaceous boundaries of arable fields) | *Mowing and alien management plan*  Mowing regimes on berms:  - mowing once in early summer  - mowing once in late summer  - mowing 2-4 times during summer  Mowing regimes on embankments: from 1 to 3 cuts annually  (no biomass removal in all cases)  Herbicide treatment: herbicide spraying or not on embankments | A single late mowing within berms was linked to high values of Ellenberg’s ecological indicator value for nutrients, while a single early mowing was associated with low values. (text p. 38, table 2)  Mowing or herbicide treatments within embankments was not significantly associated with functional traits of the vegetation in berms and embankments. (text p. 38, table 2) | e,f |
| ACTU_GREY_LITT_333 | Chaudron et al., 2016 | Medium | France | Vascular plants | Road | Road verges  (herbaceous boundaries of arable fields) | *Mowing and alien plant management*  Berm mowing:  - 1 cut in early summer  - 1 cut in late summer  - at least 2 cuts during the summer  Embankment mowing:  - number of mowing operations per year (1 to 3)  Herbicide treatment  - no herbicide on embankment  - herbicide treatment on embankment | One early mowing in berms increased species richness compared with a minimum of two cuts during summer. Species richness in embankments increased when berms were mown later and when herbicide was applied in embankments. (text p. 650, table 2)  Species evenness decreased both in berms and embankments with an increase in mowing frequency. Abundance gradient between each pair of elements was not influenced by any management practice. (text p.650, table 2)  Berm/embankment balanced variation decreased with an increase in embankment mowing frequency. (text p.650, table 2) | e,f |
| REVIEW2018_34 | Gannon & Yelverton, 2011 | Medium | USA | *Paspalum notatum*** | Road | Roadsides | *Mowing and alien plant management*  Mowing and herbicide treatments:  - no treatment  - mowing  - mowing + herbicide  Herbicide application method:  - Burch wet-blade system  - low volume application of herbicide (Weedbug)  - broadcast spraying | Mowing alone did not increase phytotoxicity or consistently affected vegetative  height compared to the nontreated control. However, it tended to increase  seedhead production (although this result could be explained by above-normal  rainfall according to the authors). (table 1-2,4-5)  The effects of the three herbicide application equipment depended on the year  and time of the season but overall, they increased phytotoxicity and reduced  vegetative height as well as seed head production and height compared to the  mowing only treatment and untreated control. The impact of broadcast spray seemed more important but no clear and consistent pattern emerged between the plant growth regulator treatments. (table 1-2, 4-5) | e,f |
| ACTU_WOS_T_2607 | Jeffries et al. 2017 | Medium | USA | *Lolium arundinaceum* | Road | Roadside ROWs | *Mowing and alien plant management*  Mowing:  - control  - mowing 23cm  - mowing 30cm  Herbicide treatment:  - control  - imazapic  - imazapic + clopyralid + triclopyr  (mowing x herbicide) | Chemical inputs and mowing intervention height did not affect tall fescue cover compared to controls from 0 to 56 days after treatment. After 84 days, compared to the control with no mowing and no herbicide application, the imazapic treatment alone at 23cm mowing height and the combined herbicide treatment at both 23cm and 30cm mowing heights improved the cover of tall fescue. (text p. 1767, table 1)  The number of seedheads was significantly reduced by mowing and herbicide application compared to the controls (see table 1) | e,f |
| EM_274 | Lanciaux, 2013 | Medium | France | Vascular plants | Road | Roadsides | *Mowing and alien plant management*  Typical ‘intensive’ management regime vs altered management regime (late mowing and measures against invasive *Robinia pseudoacacia*) | Species richness was higher under the management regime including late mowing and measures against *R. pseudoacacia* compared to a typical management regime. (text p. 30, Fig. 10)  Cover of annual and biannual species was significantly lower under the typical  management regime. (text p. 33-34, Fig. 14,15) | e,f |
| ACTU_WOS_T_10625 | Meffin et al., 2015 | Medium | New Zealand | *Brassica* spp.*** | Road | Roadsides | *Mowing and alien plant management*  - Mowed vs unmowed sites  - Sites sprayed with herbicide vs sites not sprayed | Brassica populations were less likely to be present on mowed roadsides for all three years, while they were more likely to occur on sites sprayed with herbicide but only in 2012. (table 3)  The survival of brassica populations did not seem to be affected by mowing, while herbicide spraying was associated with a reduction in the annual probability of disappearing. (text p. 123, table 4) | e,f |
| REVIEW2018_38 | Milakovic & Karrer, 2016 | Low | Austria | Soil seed bank of  *Ambrosia artemisiifolia*** | Road | Highway and non-highway roadsides | *Mowing (for alien plant management)*  - no mowing  - 1^st^ cut before flowering and 2^nd^ cut beginning of seed set  - 1^st^ cut beginning of flowering and 2^nd^ cut beginning of seed set  - 1^st^ cut before start of flowering, 2^nd^ cut before the onset of male mass flowering and 3^rd^ cut beginning of seed set  - 1^st^ cut before start of flowering, 2^nd^ cut after  the beginning of female mass flowering and 3^rd^ cut beginning of seed set | The number of seeds per m2 of the invasive *A. artemisiifolia* was significantly  higher in the unmown treatment compared to the numbers before the experiment or for the various mowing regimes.  Mowing either reduced the number of seeds compared to the values measured  before the experiment (treatment 3 and 4) or did not yield significant  differences after the three years (treatment 2 and 5). (Fig. 1) | e,f |
| WOS_20663 | Milakovic et al., 2014 | Medium | Austria | *Ambrosia artemisiifolia*** | Road | Highway and non-highway roadsides | *Mowing (for alien plant management)*  - no mowing  - 1^st^ cut before flowering and 2^nd^ cut beginning of seed set  - 1^st^ cut beginning of flowering and 2^nd^ cut beginning of seed set  - 1^st^ cut before start of flowering, 2^nd^ cut before the onset of male mass flowering and 3^rd^ cut beginning of seed set  - 1^st^ cut before start of flowering, 2^nd^ cut after  the beginning of female mass flowering and 3^rd^ cut beginning of seed set | Cutting treatment had a significant impact on the number of female flowers, for  almost all years. (text p. 259, Fig. 1A-C)  Treatment 3 (two cuts with the first after flowering) and 5 (three cuts) were  the most effective in reducing the average number of female flowers per plant,  while treatment 4 (three cuts with the second before the onset of male mass  flowering) had the most impact overall on number of male inflorescences.  (text p. 259, Fig. 1D-G)  A highly significant effect of treatment on male and female phenology was found at all sites in all years of the experiment (see Fig. 2 for details).  (text p. 259, Fig. 2) | e,f |
| ACTU_WOS_T_7397 | Pellegrini et al., 2016 | Medium | Italy | Vascular plants | Road | Roadsides | *Mowing and alien plant management*  Mowing vs herbicide (glyphosate) treatement | For the Shannon, Simpson and Equipartition indices they were no differences between the mechanical mowing and the chemical treatment with the herbicide. (table 3) | e,f |
| REVIEW2018_47 | Renz & DiTomaso, 2006 | Medium | USA | *Lepidium latifolium*** | Road  River | Roadside  River floodplain | *Mowing and alien plant management*  - untreated control  - mowing  - herbicide treatment (2,4D or glyphosate or chlorsulfuron at various rates)  - mowing + herbicide | Mowing alone did not significantly reduce perennial pepperweed biomass or  density 1 year after treatment on roadsides or in the floodplain. (text p. 34, table 2,3)  The effectiveness of the herbicide treatments alone on pepperweed biomass and density varied between the type and rate of herbicide used on roadsides and in the floodplain sites. (see table 3 for details)  Treatments combining mowing and herbicide application showed enhanced control with significant reductions in perennial pepperweed biomass and density compared with non-mowed areas. (text p. 34, table 4) | e,f |
| REVIEW2018_44 | Young, 2003 | Medium | USA | *Centaurea solstitialis*** | Road | Highway verges | *Mowing and Alien plant management*  - untreated control  - single mowing at spiney stage  - single mowing at early flowering | Mowing at the spiney stage or at early flowering was not found to effectively reduce the number of yellow starthistle plants. The number of buds in 2002 was significantly lower compared to the untreated site only when mowing was done at the spiney stage. (table 1.36) | e,f |
| REVIEW2018_42 | Young & Claassen, 2008 | Medium | USA | Vascular plants  *Centaurea*  *soltistialis*** | Road | Roadside | *Mowing, burning and alien plant management*  Different control strategies against *Centaurea solstitialis*:  - control (no management)  - burn  - mow  - spray herbicide (clopyralid)  - burn + mow  - mow + spray  - spray + burn  - burn + mow + spray | Between 2005 and 2006, neither high- (combinations of mowing, spraying  herbicide and burning) or low- (only one of the three treatment) intensity  treatments reduced the cover of annuals. However, high-intensity treatments  significantly increased the cover of native perennial grasses and both high-  and low-intensity treatments reduced the cover of the invasive *Centaurea*  *soltistialis.* Yet, the reduction in *C. soltistialis* cover was more important in  high-intensity treatments. (text p. 359, table 1) | e,f |
| 2308 | Entsminger et al., 2019 | Medium | USA | Herbaceous and woody species | Road | Highway ROWs | *Mowing and Revegetation of verges*  - 4 or more mowings annually (May, July, September, November)  - 1 mowing during fall (November)  - 1 mowing during fall (November) + supplemental native wildflower seeding | The herbaceous and woody vegetation percent coverage did not differ for the  three height categories among treatments. (text p. 24, table 1)  There were no significant differences in woody plant stem densities among  treatments. (text p. 26, table 2) | e,g |
| WOS_5898 | Coiffait-Combault et al., 2011 | Low | France | Vascular plants | Pipeline | Steppe disturbed by pipeline construction | *Grazing and Revegetation of verges*  - Grazing vs no grazing  - Hay transfer vs no hay transfer | Species richness for the 3 years was significantly higher on pipeline quadrats  with hay than pipeline quadrats without hay. There was no effect of grazing *per se* for pipeline quadrats, but for the second and third year grazing had a  significant positive effect on species richness of pipeline quadrats with hay.  (text p. 218, Fig. 5) | e,g |
| 2574 | Janssen et al., 2019 | Medium | France | Vascular plants | River | Riverbanks | *Clearing and Bank engineering*:  - relict bars repeatedly cleared  - newly reprofiled banks  - naturally rejuvenated bars  Clearing technique: brush clearing vs plowing | Annuals and alien species richness were significantly higher on bars maintained by plowing than by brush clearing. The association of maintenance technique with cover however was not significant. (text p. 1318, table 1-2; see also table 2 and text p. 1318-1319 for interaction between the type of maintenance and environmental gradients)  Alien cover was significantly higher on reprofiled banks and on naturally  rejuvenated bars than on relict bars. Hydrochorous species richness and cover  were significantly lower on naturally rejuvenated bars than on repeatedly  cleared bars. Annual richness however was similar across geomorphic  surface types. (text p. 1319-1321, table 4; see also table 2 and text p. 1318-1319 for interaction between the type of surface and environmental gradients) | d,e |
| REVIEW2018_41 | Skousen & Venable, 2008 | Medium | USA | Seeded native and non-native plants  Vascular plants | Road | Newly constructed or established highway ROWs | *Revegetation of verges, Mowing and alien plant management*  Fertilization: application vs no application of a fertilizer  Seeding: four distinct seed mixtures with varying proportions of native species and no seeding control  Mowing and alien plant management  For established sites:  - mowing + seeding  - tillage + seeding  - herbicide treatment +  seeding  - only seeding  - no disturbance and no  seeding control | Fertilization treatment in the new sites did not affect total or seeded native  plant cover. In the established sites, it increased total plant cover in only  one of three sites (Elkins), but had no effect on seeded native plant cover.  (text p. 4, table 4-5)  Seeding treatments affected total plant cover at only one of the three new  sites (Baker), where plots treated with DOH and DOH-native seed mix had a  higher total plant cover than unseeded plots. Native plant cover on the other  hand was increased by native and native, DOH-native and 1/2DOH-native seed mix treatments in Baker and Parkesburg respectively. (text p. 4-5, table 4-5)  In established sites, total plant cover was lower in herbicide plots compared  to the other treatments at Elkins and Watson. At Buckhannon both herbicide and till plots had lower cover than other treatments. Seeded native cover was  higher at all three sites for herbicide and till plots, and also for plots subjected to mowing in Elkins. (text p. 6, table 6-7)  The absence of effect of fertilization and the increase of seeded native covers  after tillage and herbicide application were mostly consistent when looking at  individual species cover for four of the prominent seeded native species and  four of the non-native species. (see table 8) | e,f,g |
| WOS_13044 | Holl & Crone, 2004 | Medium | USA | Understorey riparian species | River | Riparian forest | *Revegetation of verges and alien plant management*  - remnant forest (reference)  - restored sites (newly or older – planting of native woody species and removal of invasives) | Total cover, native cover and native richness of understorey species were  significantly higher in reference sites compared with newly and older restored  sites. However, they were no differences in cover and richness of exotic  species between restored sites and reference sites. (table 1)  At the site level, time since restoration did not explain a significant amount  of native or exotic species richness or cover. (text p. 929) | f,g |
| WOS_21621 | Muranaka, 2009 | Medium | Japan | Riparian vegetation  *Aster kantoensis*  *Eragrostis curvula***  *Diodia teres*** | River | Gravelly floodplains | *Revegetation of verges and Alien plant management*  Seed sowing for restoration of population of *Aster kantoensis* (endangered endemic) in different conditions regarding:  - flood frequency  - substrate type (gravel, sand)  Alien plant management:  - mechanical removal of invasive grasses (mostly *Eragrostis curvula)* | The type of substrate had a significant impact on survival, flowery, seed  production both in 2003 and 2004 and mean fitness, with higher values on sandy compared with gravelly substrate except for seed production in 2003.  (text p. 13, table 1)  Percentage flowery in 2003, seed production in both 2003 and 2004, and mean  fitness showed significantly higher values at habitats with alien plants  control than those without control measures. Significant interactions between alien control and substrate type were also found for seed production in 2003, survival in 2004 and mean fitness. (text p. 13, table 1)  Vegetation cover and coverage of the two invasive *E. curvula* and *D. teres*  showed relatively higher values in habitat conditions without alien plants  control (text p. 14, table 3) | f,g |
| WOS_26599 | Ruwanza et al., 2013 | Medium | South Africa | Riparian vegetation | River | Riversides | *Alien plant management and Revegetation of verges*  Clearing of the invasive tree *Eucalyptus camaldulensis:*  - fell and remove  - fell and stack burn  - control sites: invaded or natural sites  Revegetation of verges:  - passive restoration  - active restoration (seed sowing or planting of cuttings of nine native pioneers) | Both in spring 2011 and summer 2012, native cover was higher in ‘fell & removal’ sites than invaded control sites but significantly lower than natural control sites. Native cover after the ‘fell & stack burning’ treatment however was similar to the one found in invaded control sites. (table 2; see also results for different life forms)  In spring 2011, alien species cover in 1m2 plots was the highest after the ‘fell  and removal’ treatment and intermediate after the ‘fell & stack burning’  treatment. For the 25m2 plots, the pattern was similar except that invaded  plots had a similar alien cover than the ‘fell and removal’ group.  In summer 2012, both clearing treatments had higher alien cover than natural  controls, although it was lower than the one found on invaded 25m2 control  plots. (table 2; see also results for different life forms)  Species richness, Shannon and Simpson indices of diversity showed  significant differences between clearing treatments, with significant  interactions with season. Overall, ‘fell & removal’ sites tended to show the  highest diversity, while ‘fell & stack burning’ sites showed intermediate values  compared to invaded and natural controls. Levels of evenness on the other hand were similar across treatments and season. (Fig. 2)  Germination differed among clearing treatments for most seeded species, with the highest germination rates observed in ‘fell and stack burning’ sites compared to fell and removal sites and invaded controls. (text p. 137, see table 3 for detailed results)  Species showed no significant differences in mortality rates however amongst  the different clearing treatments. (text p. 137, Fig. 3)  There was no recruitment of native species in the passive restoration sites. (text p. 137) | f,g |
| WOS_34352 | Young & Claassen, 2008 | Medium | USA | Grass and forb species | Road | Highway ROWs | *Alien plant management and Revegetation of verges*  Seeding (native perennial grass):  - dryland seedmix  - wetland seedmix  Cultivation:  - disc or non-disc cultivation  Herbicide treatments:  - glyphosate  - glyphosate + clopyralid  - glyphosate + clopyralid + chlorsulfuron)  (all treatments were crossed) | Herbicide regimes seemed effective in increasing the cover of native grass and  reducing the cover of annual forbs across most treatment combinations. Annual  grasses however were less consistently affected by herbicide treatments.  The impact of disc cultivation and seedmix on the cover of weeds and native  grasses were less robust overall, being significant in fewer site x year x burn  status combinations. (see table 3 and 4 for detailed results and interactions) | f,g |
| 1218 | Ye et al., 2020 | Medium | China | Riparian vegetation | River | Dam reservoir riparian zone | *Revegetation of verges and Stream flow regulation*  Natural revegetation vs active revegetation with flooding resistant plants  Stream flow regulation:  Flooding regime:  - extreme flooding zone (flooded 286 days per year on average)  - severe flooding zone (flooded 237 days per year on average)  - moderate flooding  zone (flooded 169 days per year on average)  - non flooding-zone | *Effects of flooding regime*  Plant coverage and aboveground biomass were significantly higher in the  severely flooded zone (SFZ) and moderately flooded zone relative to the non  flooded zone (NZF), whereas the effect of increased flooding duration on plant  diversity and species richness were significantly negative with respect to the  NFZ. (text p. 4, Fig. 3)  *Effects of revegetation*  There were no significant differences in plant coverage, diversity, above-ground biomass, or species richness between the revegetation areas and the natural regeneration areas.  Mean post-flooding recoveries of plant coverage, diversity, and species  richness were small but significantly positive in both the revegetation areas  and natural regeneration areas. Recoveries of aboveground biomass were negative in the revegetation area but non-significant in the natural regeneration areas. (text p. 5-6, Fig. 6, table 3) | a,g |
| WOS_31636 | Van Looy, 2011 | Medium | Belgium | 40 target species for dry river grasslands | River | Dry river grasslands | *River restoration and Revegetation of verges*  River grasslands restoration:  - removal of upper layers or the addition of primary substrate, initiating a primary succession on gravel or sand  - addition of alluvial soils, inducing a secondary succession  - artificial seed provision with hay or topsoil of relict dry river grasslands | Restoration practices had different impacts on site species richness, target species richness and dissimilarity. (table 1)  Alpha diversity was highest in the enhanced techniques (hay or topsoil transfer) at the plot and site level, with intermediate values for 'secondary succession' practice. The same trend was found for beta-diversity at plot level but at site level it was significantly lower with the enhanced practice.  The overall additive species richness (gamma diversity) showed only a small difference between the three practices. (text p. 345, Fig. 1)  Both the site-level species richness and the richness in target species were higher in enhanced practices, while the opposite was true for dissimilarity.  Abundance on the other hand was not significantly different between restoration practices. (text p. 345, table 1) | c,g |
| 774 | Janssen et al., 2020 | Medium | France | Tree species | River | Riparian forest | *Channelization and Stream flow regulation*  Free-ﬂowing river with a channel mostly un-constrained vs regulated channelized river | Most of the functional traits and stand attributes were mediated by the interaction terms between the type of river and the forest stand age. Overall "channelization and flow regulation induced a more rapid terrestrialization of the river channel margins along the Rhône River and accelerated change in stand attributes, from pioneer-dominated stands to a mature successional phase dominated by non-native species."  (text p. 1, 6; see table 2-3 and Fig. 3-5 for detailed results for the numerous outcomes)  Forest structure and composition were also significantly different between the two types of river. (text p. 7, Fig. 7A) | a,b |
| ACTU_WOS_F_3315 | Harvolk  et al., 2015 | Medium | Germany | Riparian vegetation | River | Riverbanks | *Bank engineering, Traffic intensity and Stream flow regulation*  Bank engineering:  - bank protection (no bank protection, groynes, rock, pavement, wall)  Traffic intensity:  - waterway class (state of construction, size of ships that can use the waterway)  Stream flow regulation:  - river regulation (free-flowing vs regulated by dam) | River management variables explained a small but significant proportion of the variation in species composition along river banks. (text p. 470, table 4)  Rivers regulated by dams had significantly lower species richness on the river banks than free-flowing rivers. Bank protection and waterway class were not associated with species richness.  Conversely, bank protection and waterway class showed a significant association with Shannon diversity index. Shannon diversity values were higher for banks protected by groynes and pavement, while waterway class was negatively related with Shannon diversity.  No river management variables showed significant relationships with evenness on river banks. (text p. 471, table A2) | a,d,h |

*: exotic species

**: invasive species

^§^Broad categories of management interventions:

a: impact of artificial fluctuations of water levels in dam-regulated waterways

b: channelization of rivers

c: river restoration projects of channelized streams

d: bank engineering techniques to protect against erosion and stabilize stream banks

e: biomass reduction techniques

f: weeds, exotic and invasive control

g: revegetation techniques of LTI verges

h: other management interventions

**Table S2: Key results of the 38 studies included in the narrative synthesis of the question:** **Is tracheophyte biodiversity in LTI verges dependent on the surrounding landscape? (question Q5)**

| **[ref]** | **Reference** | **Risk of bias** | **Country** | **Biological group** | **LTI** | **LTI verge** | **Comparison** | **Key results** |
| --- | --- | --- | --- | --- | --- | --- | --- | --- |
| 3453 | Aguiar et al., 2018 | Medium | Portugal | Riparian woody species | River | Riversides | % of urban areas, % of extensive agriculture, % of intensive agriculture, % agroforestry systems, % of scrublands, % forests and % rocks in 250m buffer | Guild abundance varied differently with land-use parameters (text p. 7, table 2):  - the abundance of the obligate riparian guild decreased near managed forests  - the abundance of water-stress tolerant species increased near managed forest  - the Mediterranean evergeen guild increased in cover in agricultural and  urbanized landscapes and nearby shrublands and decreased with adjoining  managed forests  - species of the deciduous competitive guild were more abundant in agricultural and urbanized landscapes |
| WOS_505 | Alexander et al., 2009 | Medium | USA | *Helianthus annuus* | Road | Rural roadsides | Types of landscape: woods, grassland | Occupancy of roadsides was significantly higher in units adjacent to grasslands than woods. (text p. 1353, table2, Fig. 3a) |
| ACTU_WOS_T_2062 | Arenas et al., 2017a | Low | Spain | Perennial vegetation | Road | Motorway roadslopes (roadcuts and embankments) and road verges | Remnants of natural vegetation in fragmented vs unfragmented agricultural landscape | No differences were found between roadsides in fragmented vs unfragmented  landscapes for perennial total cover, species richness, inverse Simpson index  or the number of protected and endemic species. (Fig. 2) |
| ACTU_WOS_T_3340 | Arenas et al.,  2017b | Medium | Spain | Trees:  *Quercus ilex Quercus pyrenaica Populus nigra Fraxinus angustifolia*  *Ulmus pumila Salix spp* | Road | Motorway roadslopes (roadcuts and embankments) | Types of landscape: agricultural, forestry | Density of individuals higher on roadslopes surrounded by forested areas compared to agricultural landscapes for all species except *Salix spp.* for which associations were not significant. (table 4) |
| WOS_1292 | Arteaga et al., 2009 | Medium | Spain | Vascular plants | Road | Roadsides | Distance to nearest large urban nuclei | Non-endemic native species richness decreased on leeward slopes and introduced species richness on windward slopes with increasing distance to urban nuclei. (table 2) |
| WOS_2375 | Beerling, 1991 | Medium | UK | *Reynoutria japonica*** | River | Riverbanks | Types of landscape: natural/semi-natural, grazed grasslands, woodland, intermediate woodland (less mature trees), waste ground/colliery spoil, land drainage works | For three out of five rivers, significant differences between land-use types were found, with a lower abundance of *R. japonica* in semi-natural and grazed grasslands, and the highest abundance for waste ground/colliery spoil and land drainage works. (text p. 333,335; Fig. 2) |
| WOS_1674 | Bailleul et al., 2012 | Low | France | Seeds of *Brassica napus* | Road | Verges of one-lane or two-lane paved roads | Distance to the nearest field | The number of oilseed rape seeds per trap-site per road decreased with the distance to the nearest field. (text p. 3, table 1) |
| WOS_3374 | Borgmann & Rodewald, 2005 | Low | USA | *Lonicera maackii**  *Lonicera tatarica** | River | Riparian forests | % of urban, % forest, % agricultural within 1km radius | Percent cover of *Lonicera* was positively associated with the proportion of urban land cover within the landscape. (text p. 336, table 2)  Interaction effects were also found such that forest patches with reduced forest cover and higher levels of urbanization contained more *Lonicera* *spp.* relative to sites in more forested and less urbanized landscapes. (text p. 337, table 2) |
| 320 | Cao & Natuhara, 2020 | Low | Japan | Shrubs, herbs and grasses | River | River floodplain | % impervious surface, % forest, % farmland within 500m radius | Taxonomic Beta-diversity and functional Beta-diversity strongly decreased  between 10% and 20% of impervious surfaces (text p. 12, Figure 6)  Taxonomic Beta-diversty significantly increased beyond approximately 20% of forest cover and functional Beta-diversity significantly increased between 20% and 30% of percentage of forest. (text p. 12, Figure 6)  For both taxonomic and functional Beta-diversity the effect of the percentage of farmland was negligible. (text p. 12, Figure 6) |
| 9241 | Cao & Natuhara, 2020 | Low | Japan | *Phragmites australis*  Vegetation of reed communities | River | Riversides | % of impervious surface, % of farmland | Coverage, biomass and density of common reed were negatively affected by the proportion of impervious surface. The proportion of farmland on the other hand was only significantly correlated positively with the biomass of common reed. (table 6) |
| WOS_4889 | Cavaille et al., 2015 | Medium | France  Switzerland | Riparian vegetation | River | River embankments | % forest and semi-natural areas, % agricultural and open areas, % artificial areas, % water bodies within 500m radius | Landscape factors had no significant effect on bank species richness. (text p. 69) |
| 15639 | Chaudron et al., 2018 | Low | France | Vascular plants | Road | Road-field boundaries | % grasslands, % woodlands within 250m, 500m and 1km respectively | Both past and current proportion of woodland were associated with higher species richness on the berm of road-field boundaries. The association was not significant however for embankments. On the other hand, the association of species richness with the proportion of grassland was significantly negative for the past and significantly positive for the current measure on embankments, but not significant on berms. (see table 1 for detailed results with different buffer sizes) |
| ACTU_WOS_T_4570 | Cochard et al., 2017 | Low | France | Native grassland species (75) | Road | Managed herbaceous road verges of two-way paved local roads | Urban-rural gradient based on:  - % buildings, % roads and pavements, % woodlands (including hedges), % crops, % herbaceous vegetation within 500m radius  - proximity of herbaceous vegetation (% at 50m)  - distance to city centre | An increase in the proportion of buildings was associated with a significant decrease for grassland species richness, from 15 species per quadrat in rural settings to eight in urban contexts, while the shape of the response curve for ruderal species was opposite, but less pronounced, rising from four species in rural samples to six in urban areas. (text p. 222, Fig. 3d)  Urban species, peri-urban species and rural species groups were identified based on their relationship with the proportion of buildings (text p. 221-222, Fig. 3a-c) |
| ACTU_WOS_T_7297 | Cordero et al., 2016 | Medium | Brazil | *Ulex europaeus*** | Road | Roadsides | Distance to the nearest patch of grassland, forest (natural and plantation), anthropogenic area (urban and agricultural lands) | Gorse presence was more likely in sites closer to anthropogenic areas. (text p.7, table 2) |
| WOS_6747 | Dallimer et al., 2012 | Medium | UK | Forbs and woody species | River | Riparian corridors | Distance from the urban core | Complex trends were found between richness and distance to urban centre depending on rivers and cities considered, sometimes with non-linear relationships. (text p. 748, Fig. 4) |
| ACTU_WOS_T_2816 | Dylewski et al., 2017 | Medium | Poland | Trees and shrubs | Powerline | Electricity pylons | - Types of landscape: arable, meadows  - Distance to human settlement  - Distance to woodland | Density of non-native species was significantly higher in arable plots than meadows, while there was no difference for native species. (text p. 52, Fig. 1a)  The density of alien tree and shrub species was negatively correlated with distance to human settlements (but no effect for all species and native species only). Distance to woodland was not significantly correlated with neither overall, alien or native densities. (table 1-3) |
| WOS_8405 | Ehrenfeld & Schneider 1991 | Medium | USA | *Chamaecyparis thyoides*  Herbaceous, ferns and shrubs | Road | Roadsides | Gradient of suburban impact | The density of seedlings of *C. thyoides* decreased with suburban disturbance. (table 6)  The total number of species increased with the level of disturbance (table 8), but this was mainly due to an increase in invader species (table 9). |
| 5363 | Fekete et al., 2020 | Medium | Austria  Hungary  Romania  Slovakia  Slovenia | Orchids | Road | Verges of asphalt roads | % urban areas, % agricultural areas, % natural grasslands and pastures, % broadleaved forest, % shrublands, % semi-agricultural areas, % sparsely vegetated areas, % wetlands, % unsuitable places for orchids, % beaches, dunes, and sand plains, % mixed forests, % coniferous forests | Urban and agricultural land covers were associated with significantly lower numbers of orchid species and individuals. The same association was found for the cover of natural grasslands and pastures, broadleaved forests and semi-agricultural areas but only for species richness, not for the number of individuals. The cover of shrubland on the other hand was associated with a higher number of orchid individuals.  (text p. 13240, table 3) |
| WOS_9202 | Fernandes et al., 2011 | Low | Poland | Riparian vegetation | River | Riparian zone of riverbanks | % agroforestry (montado), % forestry, % agriculture, % urban within 30m and 200m buffers | An increase in the degradation pattern across the land use pressure gradient, from agroforestry to urban land use was found. (text p. 173, Fig. 5) |
| WOS_10265 | Garcia-Palacios et al., 2011 | Low | Spain | Vascular plants | Road | Roadside grasslands | Dispersal limitation index based on distance and quality (industrial–urban, farmland, old ﬁeld, roadside grassland, grass–shrubland mosaic, and forest) of the nearest four patches | Higher dispersal limitations values were associated with greater plant community similarities. (text p. 2813) |
| 3722 | Grella et al., 2018 | Low | Australia | Riparian vegetation | River | Urban riparian zones | Landscape categories: non-urban (< 5% imperviousness), peri-urban (5-18% imperviousness) and urban (> 18% imperviousness) | Total vegetation cover and species richness did not vary significantly between urbanization levels. Cover and number of species of weeds on the other hand varied significantly, increasing with the degree of urbanization. Conversely, cover and number of native species decreased significantly with urbanization. (text p. 5, t able 2) |
| ACTU_WOS_F_3315 | Harvolk et al., 2015 | Low | Germany | Vascular plants | River | River floodplains (banks, grasslands and alluvial forest fragments) | % grasslands, % forest, % arable land, % settlement within the study site and within 1km buffer | Bank relevé: The proportion of grasslands within 1km as well as the proportion of forest within the study site were negatively associated with evenness, whereas no significant relationships were found with species richness and Shannon diversity index.  Grassland relevé: Shannon index of landscape diversity was both negatively correlated with species richness and Shannon species diversity.  Copse relevé: The proportion of settlement was negatively associated to Shannon diversity and richness, and landscape structure diversity negatively associated with evenness.  (table A2) |
| WOS_12389 | Hayasaka et al., 2012 | Low | Japan | Vascular plants | Road | Curbside cracks of roadsides | Dominant land use type within 100m radius: urban land, dry fields, developed land, paddy fields, forest, coastal areas | Different land-use types were associated with distinct vegetation types. (text p. 128, table 1; see Appendix for species composition of vegetation types) |
| 2125 | Janssen et al., 2019 | Medium | France  Switzerland | Vascular plants | River | Riverbanks | % forest, % urban within 500m radius | Plant richness increased significantly with an increasing proportion of forest area in the surrounding landscape (text p. 5, Table 2)  Neither proportion of urban or forest areas significantly predicted plant abundance (text p. 5, table 2) |
| ACTU_WOS_T_5675 | Kurek et al., 2016 | Medium | Poland | Herbaceous species | Powerline | Electricity pylons | Types of surrounding landscape: cereals, maze, grasslands | Species richness but not Shannon diversity or Jaccard indices depended on the surrounding crop, with higher values in maze plots. (text p. 420, Fig. 2) |
| WOS_17974 | Liendo et al., 2015 | Medium | Spain | Alien species | River | Riversides (forests,  river bars, man-made slopes, terraces and helophytic communities) | Gradient in anthropogenic pressure (hydrological and morphological disturbances as proxies) | Number of invasive and level of invasion were higher in sites subjected to hydrological disturbance (also true for level of invasion and morphological disturbance). (text p. 293, Fig. 3) |
| 16526 | McLean et al., 2018 | Medium | South Africa | Alien species | Road | Roadsides | Types of landscape: agriculture, garden, curbs, urban green space, industrial | Garden roadsides had significantly higher species diversity than curbs, urban green spaces, agricultural areas and industrial areas. (text p. 74, table 1) |
| WOS_20257 | Meek et al., 2010 | Medium | South Africa | Riparian vegetation | River | Riversides | Types of landscape: grazed, agricultural, urban, natural | Measures of cover, richness, and diversity all differed significantly among land-use types. (text p. 160, table 3)  The following patterns were found for native and alien cover and species richness in transformed landscape (table 3-4, Fig. 2):  (+) alien cover adjacent in ‘agricultural’ vs adjacent ‘grazing’  (-) native cover in ‘agricultural’ vs ‘urban’ and ‘grazing’  (+) native richness in ‘urban’ vs ‘grazing’ and ‘agricultural’  Natural sites had the lowest values for alien species and the highest for native for both richness and relative cover. (Fig. 2)  Significant differences in species composition were also found between land-use types. (text p. 161) |
| ACTU_WOS_T_10625 | Meffin et al., 2015 | Low | New Zealand | *Brassica spp.* | Road | Rural roads and state highway verges | Distance to seed company and type of adjacent field (*Brassica* present or not) | The estimated probability of Brassica presence declined sharply with distance away from the nearest seed company. (text p. 123, Fig. 2)  Neither location adjacent to a cultivated *Brassica* field, nor distance to the nearest *Brassica* population in the previous year, were significantly linked to the probability of presence of *Brassica* populations. (text p. 123, Table 3) |
| WOS_20529 | Meunier et al., 1998 | Medium | France | Vascular plants | Road | Motorway verges | Types of landscape: garrigue, forest, crops | Species richness on verges was similar for the three types of landscape. (text p. 103) |
| ACTU_WOS_F_1922 | Nobis et al., 2016 | Low | Poland | Vascular plants | River | Riverbanks | - % arable, % forest, % urban, % shrub, % agricultural, % water courses  - Landscape diversity | Landscape diversity was associated with higher native species richness. Only  the proportion of pastures and water courses (PC3) was weakly positively  correlated with the species richness of native plants. (text p. 20, table 2)  The proportion of forest in the surroundings was negatively associated with the richness of invasive species, while the reverse pattern was found for the proportion of arable land, urbanized areas, shrubs and agricultural areas. Land cover diversity had a positive effect on the richness of invasive species. (text p. 21, table 2, Fig. 2) |
| ACTU_WOS_F_1840 | Schwoertzig et al., 2016a | Low | France | Riparian vegetation | River | Riverbanks | Urbanization gradient: peri-urban, suburban, urban (classification based on land cover areas used in PCA) | Total species richness, Shannon index and Simpson index significantly increased along the peri-urban - suburban - urban gradient. (text p. 1773, table 1) |
| ACTU_WOS_F_2761 | Schwoertzig et al., 2016b | Low | France | Riparian vegetation | River | Riverbanks with narrow riparian forest | Urbanization gradient: urban vs suburban vs periurban:grassland or  periurban:cropland (classification based on land cover areas used in PCA) | Total species richness was highest in urban sites and higher in sub-urban  compared to peri-urban sites. Richness of exotic species on the other hand was  comparable across the urbanization gradient. (text p. 572, Fig. 3) |
| WOS_31345 | Ullmann et al., 1995 | Low | New Zealand | Vascular plants | Road | Roadside verges | Presence vs absence of forest | The presence of forest was associated with a higher species richness and with a greater proportion of natives. (text p. 134) |
| 5312 | Viippola et al., 2020 | Low | USA | Vascular plants | Road | Roadside verges | Types of landscape: forest vs open land | Near road nanoparticle concentration was not affected by forest cover in either  Baltimore or Shenyang. (text p. 5, Fig. 2c) |
| 1170 | Wagner et al., 2020 | Low | Austria | *Alien woody species*  *Acer negundo**  *Ailanthus altissima**  *Fraxinus pennsylvanica**  *Populus x canadensis**  *Robinia pseudoacacia** | River | Urban riversides | % urban habitat and population density in 500m radius | An increase in the proportion of urban habitat was associated with a higher occurrence of *Acer negundo* and *Ailanthus altissima*. The same pattern was true for human population density (text p. 6, table 2)  *Robinia pseudoacacia* was less present in sites with a high proportion of urban habitats, while no association was found for *Fraxinus pennsylvanica* and *Populus x canadensis*. |
| WOS_34355 | Young-Mathews et al., 2010 | Low | USA | Riparian vegetation | River | River floodplain | Types of landscape: rangeland (pasture) vs cropland | Rangeland was associated with a higher plant diversity, species richness and cover invasive/noxious weeds. No impact of land use type was found however for the cover of herbaceous natives and woody perennials.  (table 1) |
| 1300 | Zelnik et al., 2020 | Medium | Slovenia | Invasive alien species | River | Riparian zones | Land use types: forest, wetland/mixed pasture, wood, swamp, arable  land/agricultural landscape/urban landscape, with gardens and ruderal areas | The land use was significantly less altered in sites without invasive alien species compared to sites with at least some individuals both for the adjacent zone and the wider catchment area. (text p. 8, table 3)  Regarding life forms (trees, annuals, perennials and vines), adjacent land use was negatively correlated with the number of annuals and land use in the catchment area was correlated with the total abundance of perennials and vines. (table 2) |

*: exotic species

**: invasive species

**Table S4: Key results of the 4 studies included in the narrative synthesis of the question: Is tracheophyte dispersal in LTI verges equal to, higher, or lower than dispersal in similar habitats away from LTIs? (question Q4)**

| **[ref]** | **Reference** | **Risk of bias** | **Country** | **Biological group** | **LTI** | **LTI verge** | **Comparison** | **Key results** |
| --- | --- | --- | --- | --- | --- | --- | --- | --- |
| WOS_17700 | Leyer, 2006 | Low | Germany | Riparian flora | River | Riversides | Seed dispersal along a gradient of declining connectivity between sites | Significant effect of connectivity on seedling number and species richness. (text p. 411, table 2) |
| 136 | Reisch et al., 2020 | Medium | Germany  Austria | *Gypsophilia repens* | River | Riverbanks | *Genetic distance*  Populations at different distances to the river | Genetic variation was not significantly correlated with geographic distance to the river. (text p. 4) |
| WOS_13142 | Honnay et al., 2010 | Medium | Belgium  Netherlands | *Sisymbrium austriacum Erysimum cheiranthoides Rorippa sylvestris* | River | Riverbanks | *Genetic distance*  Populations along only one river, at different perpendicular distance from the streamline | None of the three species showed a significant correlation between population genetic diversity and position of the population along the river. (text p. 37)  There was no significant correlations between population genetic diversity (molecular variance) and distance of the population from the streamline. (text p. 37) |
| ACTU_WOS_F_3709 | van der Meer et al., 2015 | Medium | Belgium | *Saxifraga granulata* | River | Riverbanks | *Genetic distance*  Populations at different distances to the river along two river systems | No relationship was found between genetic diversity and distance to the river for any of the river systems. (text p. 10) |

**Table S3: Key results of the study included in the narrative synthesis of the question: Do LTI verge management practices increase, decrease, or have no effect on tracheophyte dispersal in LTI verges? (question Q3)**

| **[ref]** | **Reference** | **Risk of bias** | **Country** | **Biological group** | **LTI** | **LTI verge** | **Comparison** | **Key results** |
| --- | --- | --- | --- | --- | --- | --- | --- | --- |
| WOS_33007 | Werth et al., 2014 | Medium | Austria, Germany Italy Switzerland | Riparian endangered flora:  *Myricaria germanica* | 4 major alpine rivers | Gravel riverbanks | *Gene movement*  Natural (canyon) and anthropogenic barriers (channelized river segment, reservoirs) vs no barriers | Genetic differentiation was higher between localities isolated by a barrier than between connected localities, indicating genetic isolation caused by barriers. (text p. 2508, table 2, Fig. 3)  Average FST values did not differ between connected and isolated localities when the putative barrier was a channelized river segment. (text p. 2508) |

**Table S5: Key results of the 2 studies included in the narrative synthesis of the question:** **Is tracheophyte dispersal in LTI verges dependent on the surrounding landscape? (question Q6)**

| **[ref]** | **Reference** | **Risk of bias** | **Country** | **Biological group** | **LTI** | **LTI verge** | **Comparison** | **Key results** |
| --- | --- | --- | --- | --- | --- | --- | --- | --- |
| WOS_33958 | Yager et al., 2011 | Medium | USA | *Imperata cylindrica*  ****** | Road | Roadsides | Dispersal in pine–tallgrass and pine–shrub forests | The mean dispersal distance did not significantly differ between the pine–tallgrass forest and the pine–shrub forest. (text p. 209)  The mean maximum dispersal distance was greater in the pine–tallgrass association compared to the pine–shrub association. (text p. 209)  More spikelets dispersed farther than 5 m into the pine–tallgrass forest compared to the pine–shrub forest. (text p. 209, Fig. 2)  Spikelets were less likely to be intercepted by vegetation in pine–tallgrass forests. (text p. 209, table 1) |
| ACTU_GREY_LITT_117 | Schwoertzig, 2016 | Low | France | Riparian flora | River | Riverbanks | Urbanisation and seed dispersal along a river corridor | The number of seeds was notably higher in the urban sites than in the suburban sites along the two riparian corridors. (text p.108, Fig. 4.3)  The number of seeds in the traps was three times higher in the peri-urban site than in the urban one. (text p. 108) |

**: invasive species
