# Supplementary material for: Can linear transportation infrastructure verges constitute a habitat and/or a corridor for vascular plants in temperate ecosystems? A systematic review
Source: Environ Evid. 2024 Mar 16;13:4. doi: 10.1186/s13750-024-00328-3 (PMC11376103; doi:10.1186/s13750-024-00328-3)
Supplement: Supplementary file 7 — Additional file 7. Extended narrative summary for the subquestion Q1 on the impact of management practices on the habitat role of transport infrastructure verges. [file 13750_2024_328_MOESM7_ESM.docx]

**Additional file 7.** Extended narrative summary for the subquestion Q1 on the impact of management practices on the habitat role of transport infrastructure verges. Mell *et al.*

Given the diversity of the management measures studied we provide here an extended narrative synthesis about subquestion Q1: *Do LTI verge management practices have positive, neutral or negative effect on vascular plants in LTI verges?*

1. **Regulating waterway water level**

Twenty-three studies focused on the effects of artificial fluctuations of water levels on the riparian vegetation along dam-regulated rivers. Among them, two studies had a low risk of bias. One assessed the impact of an artificial flooding event on the soil seed banks [1] and found that the number of species in soil seed banks was lower after flooding. The other measured the changes in riparian vegetation cover after the removal of a dam [2] and found no significant variation for neither of ground, shrub and tree strata two years after the intervention.

Among the studies with a medium risk of bias, a group of six studies also evaluated the effects of artificial changes to flooding exposure, all conducted in the ‘Three Gorges Reservoir’ area in China [3–8]. Overall, these studies found a negative impact of artificial flooding in terms of species richness, with the exception of a single study, which found a greater number of species in the pre-upland drawdown zone compared to the adjacent upland [5]. Yet, more consistently with similar studies, the inverse pattern was obtained between pre-riparian drawdown zone and a natural riparian control. The effects of exposure to artificial flooding regimes on plant cover were more heterogeneous, with non-significant differences or significant negative, positive, and non-linear relationship depending on the study. Studies reporting indices of diversity found either a decrease with exposure to artificial flooding [8] or no impact [3, 6], while those tracking changes in species assemblages highlighted significant differences between flooding regimes [4, 5].

Another group of eleven studies compared regulated sections of streams to either less regulated or completely unregulated watercourses. In terms of species richness, studies most consistently observed a decrease with stream flow regulation, either for overall species richness [9, 10] or for some specific groups such as perennials or natives [11, 12]. In one instance species richness was higher in the soil seed bank of highly regulated rivers, but it concerned exotic species [13]. Consistently with the previous results, several studies looking at species assemblage found an increase in the proportion of exotic species and species adapted to disturbance [13–15]. More generally, studies consistently highlighted that stream flow regulation was associated with differences in the structure of vegetation communities. Studies assessing the effects of stream flow regulation on plant cover or diversity indices reported contrasted results, positive [14, 16, 17] in some cases and negative in others [9, 11, 17].

Lastly, three studies evaluated the impact of dam-regulation by assessing changes in riparian vegetation before and after the construction of the dam. One study found a lower overall diversity of woody species as measured by the Shannon-Wiener index for the post-dam period [18], as well as different pattern of diversities between the pre- and post-dam periods depending on guild and geomorphological attributes. Another study found a lower overall species richness and a higher alien species understory cover during the post-dam period in *Galio sylvatici-Carpinetum* and *Salici-Populetum* habitats, but no differences pre- and post-dam construction for *Salicetum triandro-viminalis* and *Querco-Ulmetum minoris* plots. The third study reported significant variations in the relative proportions of riparian guilds before and after dam-construction but not in diversity for most of the guilds [19].

[1] Lajeunesse MJ, Koricheva J, Gurevitch J, et al. Recovering missing or partial data from studies: a survey of conversions and imputations for meta-analysis. In: Koricheva J, Gurevitch J, Mengersen K (eds) *Handbook of Meta-analysis in Ecology and Evolution*. Princeton, New Jersey, USA, pp. 195–206.

[2] Hedges LV, Olkin I. *Statistical method for meta-analysis*. Academic Press, 1985.

[3] Hedges LV. Distribution theory for Glass’s estimator of effect size and related estimators. *J Educ Stat* 1981; 6: 107–128.

[4] Hamman EA, Pappalardo P, Bence JR, et al. Bias in meta-analyses using Hedges’d. *Ecosphere* 2018; 9: e02419.

[5] Hedges LV. Estimation of effect size from a series of independent experiments. *Psychol Bull* 1982; 92: 490.

[6] Nakagawa S, Santos ES. Methodological issues and advances in biological meta-analysis. *Evol Ecol* 2012; 26: 1253–1274.

[7] Egger M, Smith GD, Schneider M, et al. Bias in meta-analysis detected by a simple, graphical test. *Bmj* 1997; 315: 629–634.

[8] R Core Team. *R: A Language and Environment for Statistical Computing*. Vienna, Austria: R Foundation for Statistical Computing, https://www.R-project.org/ (2022).

[9] Viechtbauer W. Conducting meta-analyses in R with the metafor package. *J Stat Softw* 2010; 36: 1–48.

[10] Huc S, Van Es J, Abdulhak S, et al. Quel est l’effet d’une crue sur la banque de semences du sol ?

[11] Stephens JL. Short-Term Response of Vegetation and the Riparian Bird Community to Dam Removal on the Rogue River, Oregon. *Ecol Restor* 2017; 35: 328–340.

[12] Chen Z, Yuan X, Roß-Nickoll M, et al. Moderate inundation stimulates plant community assembly in the drawdown zone of China’s Three Gorges Reservoir. *Environ Sci Eur* 2020; 32: 1–11.

[13] Su X, Bejarano MD, Yi X, et al. Unnatural flooding alters the functional diversity of riparian vegetation of the Three Gorges Reservoir. *Freshw Biol* 2020; 65: 1585–1595.

[14] Su X, Zeng B, Huang W, et al. Effects of the Three Gorges Dam on preupland and preriparian drawdown zones vegetation in the upper watershed of the Yangtze River, PR China. *Ecol Eng* 2012; 44: 123–127.

[15] Yang F, Zhang D, Wu J, et al. Anti-seasonal submergence dominates the structure and composition of prokaryotic communities in the riparian zone of the Three Gorges Reservoir, China. *Sci Total Environ* 2019; 663: 662–672.

[16] Zhang A, Cornwell W, Li Z, et al. Strong restrictions on the trait range of co-occurring species in the newly created riparian zone of the Three Gorges Reservoir Area, China. *J Plant Ecol* 2019; 12: 825–833.

[17] Ye C, Butler OM, Chen C, et al. Shifts in characteristics of the plant-soil system associated with flooding and revegetation in the riparian zone of Three Gorges Reservoir, China. *Geoderma* 2020; 361: 114015.

[18] Beauchamp VB, Stromberg JC. Changes to herbaceous plant communities on a regulated desert river. *River Res Appl* 2008; 24: 754–770.

[19] Harvolk S, Symmank L, Sundermeier A, et al. Human impact on plant biodiversity in functional floodplains of heavily modified rivers–A comparative study along German Federal Waterways. *Ecol Eng* 2015; 84: 463–475.

1. **River channelization**

Five studies assessed the impact of river channelization on the riparian vegetation. Among them, two had a low risk of bias [20, 21]. The first study compared channelized to non-channelized sites and found no difference regarding species richness, Simpson’s index of diversity or wetland species prevalence. The other one evaluated the impact of canal dredging and found an increase in species richness and mean plant cover the year following dredging, but differences were no longer significant two years after the intervention [21]. Two of the studies with a medium risk of bias also compared channelized rivers with more ‘natural’ streams [22, 23]. Both found a higher representation of exotic species among riparian communities of channelized streams, although for one study this pattern appeared only for the inner part of the riverbank [24]. The third study with a medium risk of bias compared the vegetation on river walls to plant communities of the intertidal foreshore along an urban river and found that the former were more species rich [25].

[20] Foard M, Burnette DJ, Burge DR, et al. Influence of river channelization and the invasive shrub, Ligustrum sinense, on oak (Quercus spp.) growth rates in bottomland hardwood forests. *Appl Veg Sci* 2016; 19: 401–412.

[21] Stępień E, Zawal A, Buczyński P, et al. Effects of dredging on the vegetation in a small lowland river. *PeerJ* 2019; 7: e6282.

[22] Aguiar FC, Ferreira MT. Human-disturbed landscapes: effects on composition and integrity of riparian woody vegetation in the Tagus River basin, Portugal. *Environ Conserv* 2005; 32: 30–41.

[23] Janssen P, Stella JC, Piégay H, et al. Divergence of riparian forest composition and functional traits from natural succession along a degraded river with multiple stressor legacies. *Sci Total Environ* 2020; 721: 137730.

[24] Aguiar F, Ferreira M, Moreira I. Exotic and native vegetation establishment following channelization of a western Iberian river. *Regul Rivers Res Manag Int J Devoted River Res Manag* 2001; 17: 509–526.

[25] Francis RA, Hoggart SPG. Waste not, want not: The need to utilize existing artificial structures for habitat improvement along urban rivers. *Restor Ecol*. Epub ahead of print 2008. DOI: 10.1111/j.1526-100X.2008.00434.x.

1. **River restoration**

Ten studies investigated the impacts of various restoration projects of channelized rivers. Two of them evaluated changes in riparian vegetation before and after the restoration measures [26, 27]. One study found changes in plant cover and species richness between the two periods, but the exact patterns varied depending on the type of vegetation (stratum, riparian, gramineous) and which bank were sampled [26]. The other reported an increase in species richness, which was however no longer significant two years after restoration (lowering of flood defence) [27]. Changes in species assemblage remained significant over the study period and reflected a decrease in taxa with stronger ruderal traits as well as an increase in moisture tolerant species. The remaining studies compared riparian vegetation in restored sections to either unrestored, ‘near-natural’ reference sections, or areas restored using a distinct restoration measure. The association of restoration status with species richness was positive in one study [28], varied in another study between positive, neutral or negative depending on the river sites considered [29], and was positive but only for lowland river floodplains (not large mountain river floodplains) in a third study [30]. Associations with species diversity were either found to be neutral [31] or positive [28]. Regarding the impacts of distinct restoration measures, one study found no significant differences in plant cover between riverbanks submitted to either the creation of gravel groynes, the deposition of sand and loam, or riprap embankment (i.e. surface layer of rock or other loose material used to protect river embankment) removal [32]. Yet, a separate study comparing similar restoration measures found that vegetation cover on riversides after riprap embankment removal was lower than on riverbanks with added sand [28]. River grasslands restoration practices were also compared in another study [33] and showed that enhanced techniques involving hay or topsoil transfer were associated with the highest alpha diversity values, compared to techniques based only on the addition of sand, gravel or alluvial soils. Another study focusing on reconstruction methods of river dykes found that using imported clay was associated with a lower species richness compared with methods using intact sods, former topsoil or former subsoil [34]. Impacts of projects involving one or more techniques like river widening, remeandering, recreation of instream structure, flow restoration and reconnection of side channels have also been investigated and were found to be neutral based on taxonomic diversity indices [35]. Lastly, regarding species assemblage, differences in restoration status and techniques were almost systematically reflected through measures such as species composition, relative abundance or guild composition.

[26] Baattrup-Pedersen A, Riis T, Hansen HO, et al. Restoration of a Danish headwater stream: short-term changes in plant species abundance and composition. *Aquat Conserv Mar Freshw Ecosyst* 2000; 10: 13–23.

[27] Richards DR, Moggridge HL, Warren PH, et al. Impacts of hydrological restoration on lowland river floodplain plant communities. *Wetl Ecol Manag* 2020; 28: 403–417.

[28] Strobl K, Wurfer A-L, A.-L, et al. Ecological assessment of different riverbank revitalisation measures to restore riparian vegetation in a highly modified river. *TUEXENIA*; 35.

[29] Rohde S, Schütz M, Kienast F, et al. River widening: an approach to restoring riparian habitats and plant species. *River Res Appl* 2005; 21: 1075–1094.

[30] Modrak P, Brunzel S, Lorenz AW. Riparian plant species preferences indicate diversification of site conditions after river restoration. *Ecohydrology* 2017; 10: e1852.

[31] Toth LA, van der Valk A. Predictability of flood pulse driven assembly rules for restoration of a floodplain plant community. *Wetl Ecol Manag* 2012; 20: 59–75.

[32] Bauer M, Harzer R, Strobl K, et al. Resilience of riparian vegetation after restoration measures on R iver I nn. *River Res Appl* 2018; 34: 451–460.

[33] Van Looy K. Restoring river grasslands: Influence of soil, isolation and restoration technique. *Basic Appl Ecol* 2011; 12: 342–349.

[34] Liebrand C, Sykora K. Restoration of semi-natural, species-rich grasslands on river dikes after reconstruction. *Ecol Eng* 1996; 7: 315–326.

[35] Göthe E, Timmermann A, Januschke K, et al. Structural and functional responses of floodplain vegetation to stream ecosystem restoration. *Hydrobiologia* 2016; 769: 79–92.

1. **Riverbanks engineering**

Ten studies assessed the impact of the presence of different types of engineering structures used for stabilization and protection against the erosion of riverbanks. The study by Dufour et al. [36] reported that embanked reaches constrained by dykes on both banks had similar stem densities of ligneous species than unconstrained reaches. Vegetation communities were distinct however with a higher index of similarity in embanked reaches and a relatively high number of species unique to unconstrained reaches. Pettifer and Kay [37] found that banks protected by flood defences had lower species richness, Simpson index and species abundance than unprotected banks. No particular trend was found however with regard to the age of the flood defence installation. Janssen et al. [38] reported a greater cover of exotic species in reprofiled banks compared to relict bars, but the same pattern was also true between naturally rejuvenated bars and relict bars.

Five studies compared riparian vegetation on riverbanks that had been modified using only civil engineering techniques (riprap protection), purely bioengineering techniques (*e.g.* willow fascines, vegetalized cribwalls) or mixed techniques. Overall, either mixed techniques or pure bioengineering seemed to yield the best outcomes in terms of species richness when compared to riverbanks with riprap protection [38–40]. Moreover, Tisserant et al. [41] also found that more exotic species were present on pure bioengineering sites than mixed sites. Yet, Martin et al. [42] reported a lower number of exotics in banks modified with willow fascines compared to downstream banks, whereas a similar number of exotic species were found between banks protected with ripraps or with mixed-technique and downstream banks. Wollny et al. [43] also compared banks with riprap to front-fixed banks (front-fixed banks are ripraps parallel to the shoreline and embedded in the waterway with an unsecured bank of low steepness behind them), as well as unfortified banks. They found a higher Shannon index and species richness for riprap protections compared to front-fixed banks at the Danube site, but only a greater Shannon index at the Main site. Tree coverage on the other hand was higher at front-fixed sites, whereas shrub coverage was similar across bank types as well as functional diversity. Lastly, the study by Harvolk et al. [10] with different types of protection (groynes, rock, pavement, wall or no protection) reported a higher Shannon index for banks protected by groynes and pavement. No significant differences were found in terms of species richness between protection types.

[10] Harvolk S, Symmank L, Sundermeier A, et al. Human impact on plant biodiversity in functional floodplains of heavily modified rivers–A comparative study along German Federal Waterways. *Ecol Eng* 2015; 84: 463–475.

[36] Dufour S, Barsoum N, Muller E, et al. Effects of channel confinement on pioneer woody vegetation structure, composition and diversity along the River Drôme (SE France). *Earth Surf Process Landf* 2007; 32: 1244–1256.

[37] Pettifer E, Kay P. The effects of flood defences on riparian vegetation species richness and abundance. *Water Environ J* 2012; 26: 343–351.

[38] Janssen P, Cavaillé P, Bray F, et al. Soil bioengineering techniques enhance riparian habitat quality and multi-taxonomic diversity in the foothills of the Alps and Jura Mountains. *Ecol Eng* 2019; 133: 1–9.

[39] Cavaillé P, Dommanget F, Daumergue N, et al. Biodiversity assessment following a naturality gradient of riverbank protection structures in French prealps rivers. *Ecol Eng* 2013; 53: 23–30.

[40] Cavaillé P, Ducasse L, Breton V, et al. Functional and taxonomic plant diversity for riverbank protection works: Bioengineering techniques close to natural banks and beyond hard engineering. *J Environ Manage* 2015; 151: 65–75.

[41] Tisserant M, Janssen P, Evette A, et al. Diversity and succession of riparian plant communities along riverbanks bioengineered for erosion control: a case study in the foothills of the Alps and the Jura Mountains. *Ecol Eng* 2020; 152: 105880.

[42] Martin F-M, Janssen P, Bergès L, et al. Higher structural connectivity and resistance against invasions of soil bioengineering over hard-engineering for riverbank stabilisation. *Wetl Ecol Manag* 2021; 29: 27–39.

[43] Wollny JT, Otte A, Harvolk-Schöning S. Dominance of competitors in riparian plant species composition along constructed banks of the German rivers Main and Danube. *Ecol Eng* 2019; 127: 324–337.

1. **Vegetative biomass reduction**

Forty-five studies examined the impacts of various practices (burning, grazing, clearing or mowing) used to reduce the biomass of the vegetation on LTI verges, usually as part of the maintenance of a given LTI. Ishida et al. [44] assessed the consequences of artificial burning of river grasslands and found that it maintained early successional vegetation by favoring the occurrence of herbaceous species to the detriment of woody and fern species. Milberg and Lamont [45] investigated the response of weed species in sclerophyll vegetation in highway verges to experimental fires. They found that overall weed species had increased more in cover and frequency thirty months after the fires than had the native species. The number of weed species, individuals and weed cover were also significantly greater in burnt plots than in unburnt control plots. In another study by Young and Claessen [46], the consequences of burning roadside grasslands as a mean of reducing the cover of the weed *Centaurea solstitialis* was studied either in isolation or in combination with mowing and/or herbicide spraying. A reduction in *C. solstitialis* cover was found as a result of burning both in isolation and in combination with other treatments, whereas native perennial grasses increased only when treatments were combined and the cover of annuals was not affected by the treatments.

Six studies focused on the impact of grazing on riverbanks and river floodplain pastures by comparing grazed plots to sites unexposed to livestock [47–52]. Overall, grazed sites tended to host a higher number of species than control plots without livestock access [48, 49], although some neutral associations were also reported [49, 52]. Similar results were reported for Shannon index [48, 49], whereas the study also reporting Simpson index did not detect differences between grazing treatments [49]. Ranganath et al. [47] on the other hand illustrated a positive influence of livestock exclusion on groundcover vegetation, while Schaich et al. [48] observed a higher abundance of riparian plants in sites not protected from grazing. The study by Janssen and Robertson [53] assessed the impact of grazing intensity using cowpat densities as a proxy to situate river floodplains pastures along a gradient. It showed a decrease in both total and native vegetation cover with grazing intensity. Furthermore, a study conducted by Plieninger [54] also focused on grazing intensity but this time in abandoned dehesas (a traditional agrosylvopastoral system) at roadsides, and using a grazing pressure index based on fecal counts and traces of browsing by livestock. Because the research focused on the regeneration of *Quercus ilex*, it looked at the relationship between the indicators of grazing pressure and sapling or juvenile densities but did not detect any significant effect. Kidd and Yeakley [55] on the other hand compared riparian wetlands currently subjected to grazing with sites from which livestock had been excluded in the short-term (3 years before) or in the long-term (13 years before). They showed that sites exposed to continuous livestock grazing had higher total, native but also non-native species richness than both livestock exclusion treatments. The same patterns for Shannon index were found between grazed sites and sites under long term exclusion, while only the Shannon index for native plants was greater in grazed plots than in sites under short term exclusion. Regarding plant cover, it was highest for natives in short-term exclusion sites, intermediate in continuously grazed sites and lowest in long-term exclusion sites, while the opposite pattern was found for non-native species. Interestingly, the study also reported the highest abundance values of the invasive *Phalaris arundinacea* in long-term exclusion sites. The role of the type of livestock (sheep or cows) in the vegetation response to grazing was also investigated experimentally in a study by Mcdonald [56]. It showed that after a few years of survey, significant differences in species composition appeared between the livestock treatments. Lastly for grazing studies, Coiffait-Combault et al. [57] reported results on the impact of grazing on steppe habitats that had been disturbed by the construction of a pipeline. They found that after two and three years, grazing had a positive influence on the species richness of quadrats above the pipeline that had also received a hay transfer. However, there was no influence of grazing per se independently of the hay treatment.

Two studies focused on the consequences of vegetation clearing and slashing of LTI verges. Clark and White [58] highlighted significant temporal changes in vegetation communities between a powerline corridor that had been recently cut and a reference site protected from slashing since multiple years. Janssen et al. [59] on the other hand investigated the respective impacts of two clearing techniques (brush clearing or plowing) used on relict river bars. More annuals and exotic species were observed on bars maintained by plowing than by brush clearing, while no differences in vegetation cover were detected between the two maintenance techniques.

The final group of studies in this management category concerned the impacts of various mowing regimes on LTI verges and gathered twenty-six studies, all with a medium risk of bias except for one study [60]. The latter assessed to what extent distinct mowing regimes in terms of timing and frequency (based on flowering and seed set schedules) could affect the soil seed bank of the invasive *Ambrosia artemisiifolia* along highways and non-highway roadsides. It showed that mowing negatively impacted the invasive, since the seed densities of *A. artemisiifolia* were higher in the unmown. The extent of the reduction in the size of the seed banks of the invasive varied with the number of cuts and their timing, and the most effective mowing regime consisted of one cut just after the beginning of female flowering, followed by a second cut two to three weeks later. A similar study by Milakovic et al. [61] applied the same treatments but focused on their impact on flowering. Overall, the various mowing regimes tended to decrease the number of flowers compared to unmown roadsides but the most effective combinations of number of cuts and timing depended on whether on the number of male or female flowers was considered. Three other studies evaluated the role of mowing in controlling the propagation of weeds and invasive species. Gannon and Yelverton [62] reported that mowing alone did not significantly harm the invasive *Paspalum notatum* compared to the non-treated control. Mowing might even had led in this case to an increase in seedhead production, although particular climatic conditions with above normal rainfalls could be the principal determinant. Similarly, Renz and DiTomaso [63] found that mowing alone was insufficient to reduce the biomass or density of the invasive *Lepidium latifolium* on roadsides and river floodplains. Combining mowing with herbicide application however showed enhanced control *L. latifolium* compared to unmown areas. Meffin et al. [64] on the other hand observed that mowing decreased the probability of occurrence the weeds *Brassica spp.*, although it did not seem to influence survival rates. Another subset of five studies provided information on the impact of mowing on the verges of highways specifically. Skousen and Venable [65] observed that, in two out of three highway sites studied, mown highway right-of-ways (ROWs) seeded with native and non-native plants exhibited similar total plant cover but higher seeded native cover than unmown ROWs after two growing seasons. Halbritter et al. [66] further evaluated the impact of mowing frequency on highway margins and reported lower species richness and densities for margins mown every three weeks compared to margins under no mowing or mown every six weeks. In addition to the frequency of mowing, Entsminger et al. [67] assessed the impact of the timing of the cuts in highways ROWs and they did not detect significant differences among treatments with regard to cover of herbaceous and woody vegetation, as well as woody plant stem densities. Two studies by Noordijk et al. [68, 69] included hay removal after cutting as a third parameter in addition to frequency and timing to the mowing regimes applied on highway verges. They found that the regime with mowing twice a year and hay removal had the most positive influence on plant species richness, flower diversity and abundance, although the improvement was significant only for high productive verges in one of the studies [69].

[44] Ishida S, Nakashizuka T, Gonda Y, et al. Effects of flooding and artificial burning disturbances on plant species composition in a downstream riverside floodplain. *Ecol Res* 2008; 23: 745–755.

[45] Milberg P, Lamont BB. Fire enhances weed invasion of roadside vegetation in southwestern Australia. *Biol Conserv* 1995; 73: 45–49.

[46] Young SL, Claassen VP. Release of roadside native perennial grasses following removal of yellow starthistle. *Ecol Restor* 2008; 26: 357–364.

[47] Ranganath S, Hession W, Wynn T. Livestock exclusion influences on riparian vegetation, channel morphology, and benthic macroinvertebrate assemblages. *J Soil Water Conserv* 2009; 64: 33–42.

[48] Schaich H, Rudner M, Konold W. Short-term impact of river restoration and grazing on floodplain vegetation in Luxembourg. *Agric Ecosyst Environ* 2010; 139: 142–149.

[49] Stockan JA, Langan SJ, Young MR. Investigating riparian margins for vegetation patterns and plant–environment relationships in northeast Scotland. *J Environ Qual* 2012; 41: 364–372.

[50] Van Looy K, Meire P. A conservation paradox for riparian habitats and river corridor species. *J Nat Conserv* 2009; 17: 33–46.

[51] Vogt K, Rasran L, Jensen K. Seed deposition in drift lines: Opportunity or hazard for species establishment? *Aquat Bot* 2007; 86: 385–392.

[52] Dutoit T, Moinardeau C, Mesléard F. Using Different Grazing Practices for Increasing Plant Biodiversity in the Dykes and Embankments Along the Rhne River (Southern France). *Environ Manage* 2016; 58: 984–997.

[53] Jansen A, Robertson AI. Relationships between livestock management and the ecological condition of riparian habitats along an Australian floodplain river. *J Appl Ecol* 2001; 38: 63–75.

[54] Plieninger T. Compatibility of livestock grazing with stand regeneration in Mediterranean holm oak parklands. *J Nat Conserv* 2007; 15: 1–9.

[55] Kidd S a., Yeakley J a. Riparian Wetland Plant Response to Livestock Exclusion in the Lower Columbia River Basin. *Nat Areas J* 2015; 35: 504–514.

[56] McDonald AW. Succession during the re-creation of a flood-meadow 1985-1999. *Appl Veg Sci* 2001; 4: 167–176.

[57] Coiffait-Gombault C, Buisson E, Dutoit T. Hay Transfer Promotes Establishment of Mediterranean Steppe Vegetation on Soil Disturbed by Pipeline Construction. *Restor Ecol* 2011; 19: 214–222.

[58] Clarke DJ, White JG. Towards ecological management of Australian powerline corridor vegetation. *Landsc Urban Plan* 2008; 86: 257–266.

[59] Janssen P, Piégay H, Pont B, et al. How maintenance and restoration measures mediate the response of riparian plant functional composition to environmental gradients on channel margins: Insights from a highly degraded large river. *Sci Total Environ* 2019; 656: 1312–1325.

[60] Milakovic I, Karrer G. The influence of mowing regime on the soil seed bank of the invasive plant Ambrosia artemisiifolia L. *NeoBiota* 2016; 28: 39–49.

[61] Milakovic I, Fiedler K, Karrer G. Management of roadside populations of invasive A mbrosia artemisiifolia by mowing. *Weed Res* 2014; 54: 256–264.

[62] Gannon TW, Yelverton FH. Application placement equipment for bahiagrass (Paspalum notatum) suppression along roadsides. *Weed Technol* 2011; 25: 77–83.

[63] Renz MJ, DiTOMASO JM. Early season mowing improves the effectiveness of chlorsulfuron and glyphosate for control of perennial pepperweed (Lepidium latifolium). *Weed Technol* 2006; 20: 32–36.

[64] Meffin R, Duncan RP, Hulme PE. Landscape-level persistence and distribution of alien feral crops linked to seed transport. *Agric Ecosyst Environ* 2015; 203: 119–126.

[65] Skousen J, Venable C. Establishing native plants on newly-constructed and older-reclaimed sites along West Virginia highways. *Land Degrad Dev* 2008; 19: 388–396.

[66] Halbritter DA, Daniels JC, Whitaker DC, et al. Reducing Mowing Frequency Increases Floral Resource and Butterfly (Lepidoptera: Hesperioidea and Papilionoidea) Abundance in Managed Roadside Margins. *Fla Entomol* 2015; 98: 1081–1092.

[67] Entsminger ED, Jones JC, Guyton JW, et al. Mowing effects on woody stem density and woody and herbaceous vegetation heights along Mississippi highway right-of-ways. *J Fish Wildl Manag* 2019; 10: 19–37.

[68] Noordijk J, Delille K, Schaffers AP, et al. Optimizing grassland management for flower-visiting insects in roadside verges. *Biol Conserv* 2009; 142: 2097–2103.

[69] Noordijk J, Schaffers AP, Heijerman T, et al. Effects of vegetation management by mowing on ground-dwelling arthropods. *Ecol Eng* 2010; 36: 740–750.

1. **Exotic/Weed management**

Thirty studies focused on actions taken against weeds or invasive to limit their development on LTI verges. One study with a low risk of bias, conducted by Bunn *et al.* [70] investigated the effect of artificial shading for several months on the invasive Para grass, *Brachiaria mutica*. They found that the shading treatments significantly reduced the biomass and height of the invasive species compared to the open controls, with the amount of reduction increasing with the shading intensity. However, the other studies involved control plans that relied on the mechanical removal of problematic species, the application of herbicide treatments or both in combination. Specific treatments varied widely between experiments in terms of the exact cocktails of chemical substances or the removal protocol applied. Overall, they reported systematically at least some success in containing the development of weeds and invasives, although some situations required to maintain a dedicated long term management program to avoid resurgences (*e.g.* [71]). In some cases, authors also demonstrated highly significant positive repercussions on plant communities of non-invasive species, including the native flora (*e.g.* [72–75]). In addition, four studies assessed whether revegetation of LTI verges projects in combination with control of weeds or invasive species were effective and reported mostly positive results [46, 74–76].

[46] Young SL, Claassen VP. Release of roadside native perennial grasses following removal of yellow starthistle. *Ecol Restor* 2008; 26: 357–364.

[70] Bunn S, Davies P, Kellaway D, et al. Influence of invasive macrophytes on channel morphology and hydrology in an open tropical lowland stream, and potential control by riparian shading. *Freshw Biol* 1998; 39: 171–178.

[71] Bentivegna DJ, Smeda RJ. Chemical management of cut-leaved teasel (Dipsacus laciniatus) in Missouri. *Weed Technol* 2008; 22: 502–506.

[72] Urgenson LS, Reichard SH, Halpern CB. Habitat factors and species’ traits influence riparian community recovery following removal of Bohemian knotweed (Polygonum x bohemicum). *Northwest Sci* 2014; 88: 246–260.

[73] Hulme PE, Bremner ET. Assessing the impact of Impatiens glandulifera on riparian habitats: partitioning diversity components following species removal. *J Appl Ecol* 2006; 43: 43–50.

[74] Muranaka T. The restoration of gravelly floodplain vegetation and endemic plants to riparian habitat in a Japanese river. *Landsc Ecol Eng* 2009; 5: 11–21.

[75] Ruwanza S, Gaertner M, Esler K, et al. The effectiveness of active and passive restoration on recovery of indigenous vegetation in riparian zones in the Western Cape, South Africa: A preliminary assessment. *South Afr J Bot* 2013; 88: 132–141.

[76] Holl KD, Crone EE. Applicability of landscape and island biogeography theory to restoration of riparian understorey plants. *J Appl Ecol* 2004; 41: 922–933.

1. **Revegetation of verges**

Twenty-three studies reported results of experiments using a variety of revegetation techniques on LTI verges. One study with a low risk of bias evaluated 10- and 20-year active restoration projects of riparian forests [77]. It was found that planting of saplings of mostly native tree species was effective in restoring plant communities similar to reference conserved forests, although some differences in species composition were nonetheless observed in the seed rain between certain sites. A second study with a low risk of bias found that hay transfers improved the species richness found on steppes disturbed by the construction of a pipeline [57]. Two studies with a medium risk of bias compared passive restoration of riparian vegetation to active restoration projects involving respectively seed sowing and planting of cuttings of nine native pioneer species [75] or revegetation with flooding resistant plants [8]. The first study highlighted benefits of active regeneration as no recruitment of native species occurred with passive restoration, whereas the latter study found no differences in plant coverage, diversity, above-ground biomass, or species richness between areas actively revegetated and areas under natural regeneration. Five other studies with a medium risk of bias assessed the efficacy of revegetation experiments based on planting of cuttings, individuals or strips of vegetation on verges. Two of them compared older and younger restored sites planted with woody species to remnant forest fragments [76, 78]. In both cases, they found differences in vegetation cover and species richness between the restored sites and the reference ecosystem even for older restored sites. Yet, in one of these two studies [78] time since restoration had a significant effect: the vegetation in older restoration sites was more similar to the vegetation in reference sites (remnant forest fragments) compared to younger restoration sites; which means that complete restoration may be possible in the longer term. Another study investigated whether transplants of two native shrubs on disturbed roadsides was more successful with additional fertilization or use of water-absorbing gels but found that simply watering transplants was sufficient for the greatest plant survival and growth [79]. The study by Yuan *et al.* [80] showed that planting of strips of semi-natural meadows improved the richness and diversity on riversides. Breton *et al.* [81] on the other hand investigated the restoration of riverbanks degraded by the invasive rodent *Myocastor coypus* using cuttings of different woody species and two sizes (40cm or 80cm), surrounded or not by fences. They found that survival rates were greater inside exclosures and with 80cm cuttings. Finally, the remaining studies with a medium risk of bias reported the results of seed sowing experiments. Overall, seeding of new vegetation significantly improved vegetation cover on LTI verges in most experiments, with variation in the extent of the improvement often found based on the type of seed mixture (commercial seeds or seeds collected on sites, with or without native species), the use of additional treatments (fertilization, irrigation) and their interactions.

[8] Ye C, Butler OM, Chen C, et al. Shifts in characteristics of the plant-soil system associated with flooding and revegetation in the riparian zone of Three Gorges Reservoir, China. *Geoderma* 2020; 361: 114015.

[57] Coiffait-Gombault C, Buisson E, Dutoit T. Hay Transfer Promotes Establishment of Mediterranean Steppe Vegetation on Soil Disturbed by Pipeline Construction. *Restor Ecol* 2011; 19: 214–222.

[75] Ruwanza S, Gaertner M, Esler K, et al. The effectiveness of active and passive restoration on recovery of indigenous vegetation in riparian zones in the Western Cape, South Africa: A preliminary assessment. *South Afr J Bot* 2013; 88: 132–141.

[76] Holl KD, Crone EE. Applicability of landscape and island biogeography theory to restoration of riparian understorey plants. *J Appl Ecol* 2004; 41: 922–933.

[77] Londe V, Messias MCTB, de Sousa HC. Vegetation restoration is associated with increasing forest width. *New For* 2021; 52: 129–144.

[78] McClain CD, Holl KD, Wood DM. Successional models as guides for restoration of riparian forest understory. *Restor Ecol* 2011; 19: 280–289.

[79] Petersen S, Roundy B, Bryant R. Revegetation methods for high-elevation roadsides at Bryce Canyon National Park, Utah. *Restor Ecol* 2004; 12: 248–257.

[80] Jia Y, Lian C, Jiaqi L, et al. AN ADAPTIVE MULTI-LAYERED ECOLOGICAL LANDSCAPE: THE ECOLOGICAL PLANTING OF HERBACEOUS COMMUNITIES ON RIVER REVETMENTS IN MOUNTAINOUS CITY. *Landsc Archit Front* 2020; 8: 44–58.

[81] Breton V, Forestier O, Guindon O, et al. Ecological restoration under pressure from invasive animal species: Use of Salicaceae cuttings in a river bank overrun by coypu. *River Res Appl* 2014; 30: 1002–1012.

1. **Other management practices**

Seven studies investigated the effects of a variety of other management practices. One study with a low risk of bias conducted by Rose and Webb [82] looked at the impact of building temporary roadways during pipeline construction on vegetation regeneration, by comparing recovery trends whether ballast was applied or not, for how long and at which time of the year. Their main result was that ballast-addition treatments and plots cut later in the year (September) had the lowest covers, both in dry and wet heath habitats. Flores and Osses [83] assessed differences in species diversity and richness between native riparian vegetation and riparian vegetation replaced by *Pinus radiata* plantations. Replacement was associated with lower Shannon index and species richness for tree species, was neutral for shrubs and positive for herbaceous species. In addition, exotic species were in greater number and more diverse in sites with replacement, while native riparian vegetation had a higher diversity of native species (but comparable species richness). Hosseini et al. [84] compared roadside clearing with or without earth work and found relatively little impact on plant cover but a moderating effect of the relationship between clearing width and Simpson index. Jarzyna et al. [85] reported a positive association between a higher salinity as a result of winter road maintenance and the cover of the invasive *Atriplex tatarica* in roadside verges. Le Viol [86] compared highway roadcuts with or without planted hedgerows and found that roadcuts with hedgerows hosted significantly distinct and more species rich plant communities. Harvolk et al. [10] evaluated the impact of traffic intensity on riparian vegetation by comparing different waterway classes. They did not detect differences between classes for species richness but traffic intensity seemed to have a negative influence on diversity, since higher waterway classes exhibited lower Shannon index. Finally, Mori et al. [87] showed that planting of shrub species (*Photinia × fraseri* or *Viburnum lucidum*) on roadsides concentrated the deposition of particulate matter closer to the road when compared to deposition patterns observed on lawn strips. The effects of planting density however were less consistent.

[10] Harvolk S, Symmank L, Sundermeier A, et al. Human impact on plant biodiversity in functional floodplains of heavily modified rivers–A comparative study along German Federal Waterways. *Ecol Eng* 2015; 84: 463–475.

[82] Rose R, Webb N. The effects of temporary ballast roadways on heathland vegetation. *J Appl Ecol* 1994; 642–650.

[83] Gutierrez Flores IR, Becerra Osses PI. The effect of native forest replacement by Pinus radiata plantations on riparian plant communities in Chile. *Plant Ecol Divers* 2017; 10: 65–75.

[84] Hosseini SA, Jalilvand H, Pourmajidian MR, et al. Effects of forest road clearings on understory diversity beneath Alnus subcordata L. stands in Iran. *Maejo Int J Sci Technol* 2011; 5: 241.

[85] Jarzyna I, Malecka K, Panufnik-Medrzycka D, et al. Dynamics and occurrence patterns of the tatarian orache Atriplex tatarica L.(Chenopodiaceae) at the roadsides in Warsaw, Poland. *Acta Soc Bot Pol*; 79.

[86] Le Viol I. *Dynamique et répartition de la diversité: contribution pour une meilleure intégration dans les actions de conservation: l’exemple des dépendances vertes autoroutières*. PhD Thesis, Paris, Muséum national d’histoire naturelle, 2009.

[87] Mori J, Fini A, Galimberti M, et al. Air pollution deposition on a roadside vegetation barrier in a Mediterranean environment: Combined effect of evergreen shrub species and planting density. *Sci Total Environ* 2018; 643: 725–737.
