# Supplementary material for: Can linear transportation infrastructure verges constitute a habitat and/or a corridor for vascular plants in temperate ecosystems? A systematic review
Source: Environ Evid. 2024 Mar 16;13:4. doi: 10.1186/s13750-024-00328-3 (PMC11376103; doi:10.1186/s13750-024-00328-3)
Supplement: Supplementary file 9 — Additional file 9. Graphical analysis of publication bias with funnel plots and cumulative meta-analysis. [file 13750_2024_328_MOESM9_ESM.docx]

**Additional file 9: Graphical analysis of publication bias with funnel plots and cumulative meta-analysis.**


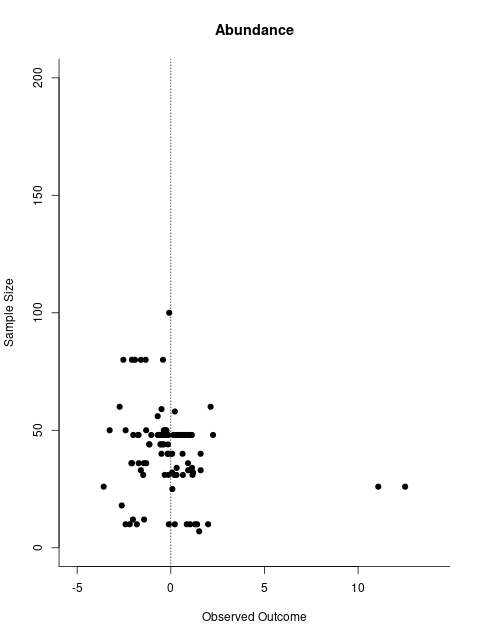

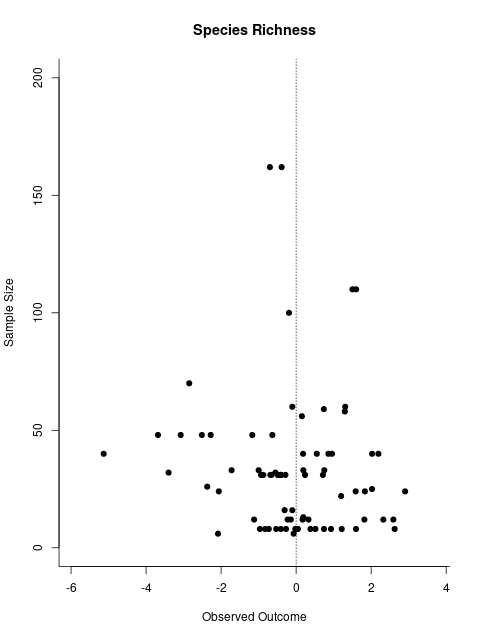


**Figure 1:** Funnel plots of the effect sizes for abundance and species richness. The grand mean effect size was estimated with a null model with cases nested in studies as random effects on intercept.


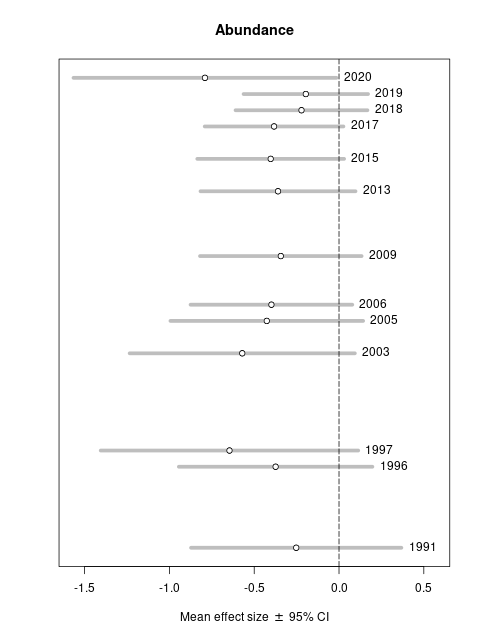

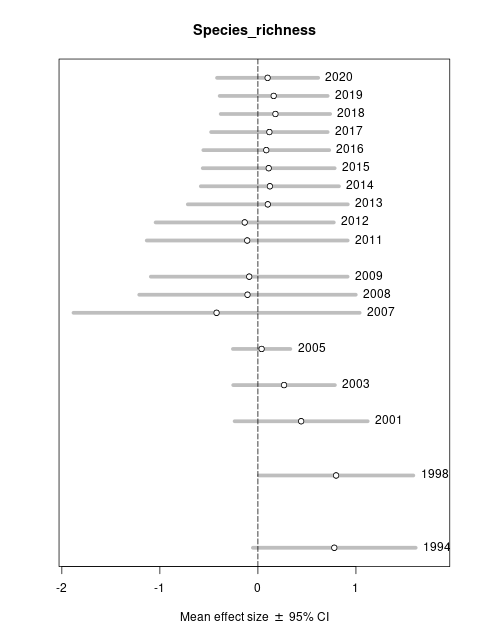


**Figure 2:** Plots for abundance and species richness of the cumulative mean effect size by publication year with 95% confidence intervals. At each year studies are added to the analysis and the new grand mean effect size and 95% CI are recalculated through a null mixed model with cases nested in studies as random effects on intercept.
