# Supplementary material for: Can linear transportation infrastructure verges constitute a habitat and/or a corridor for vascular plants in temperate ecosystems? A systematic review
Source: Environ Evid. 2024 Mar 16;13:4. doi: 10.1186/s13750-024-00328-3 (PMC11376103; doi:10.1186/s13750-024-00328-3)
Supplement: Supplementary file 11 — Additional file 11. Leave-one-out analyses and output from meta-regressions models. [file 13750_2024_328_MOESM11_ESM.docx]

**1. Leave-one-out analysis**


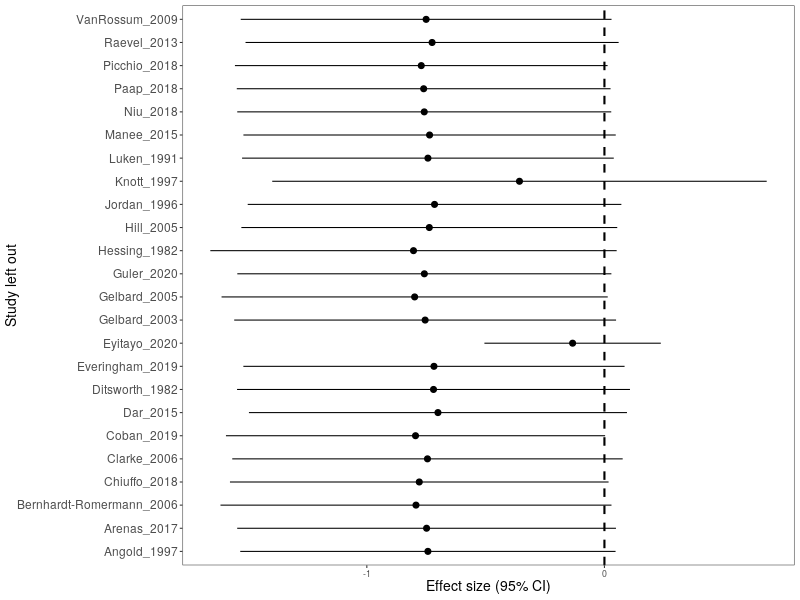
**Fig. S1** Leave-one-out analysis for the abundance data showing overall mean effects and 95% CIs based on dataset with one study left out at a time from model fitting.


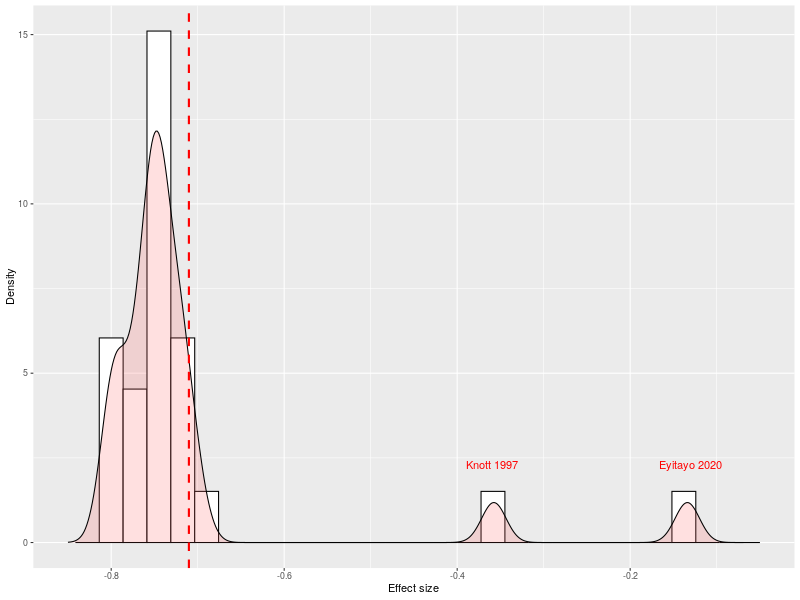
**Fig. S2** Leave-one-out analysis for the abundance data showing distribution of overall mean effects based on dataset with one study left out at a time from model fitting. Red labels highlights the two studies that yield an important change in the effect size estimate when left out.

**
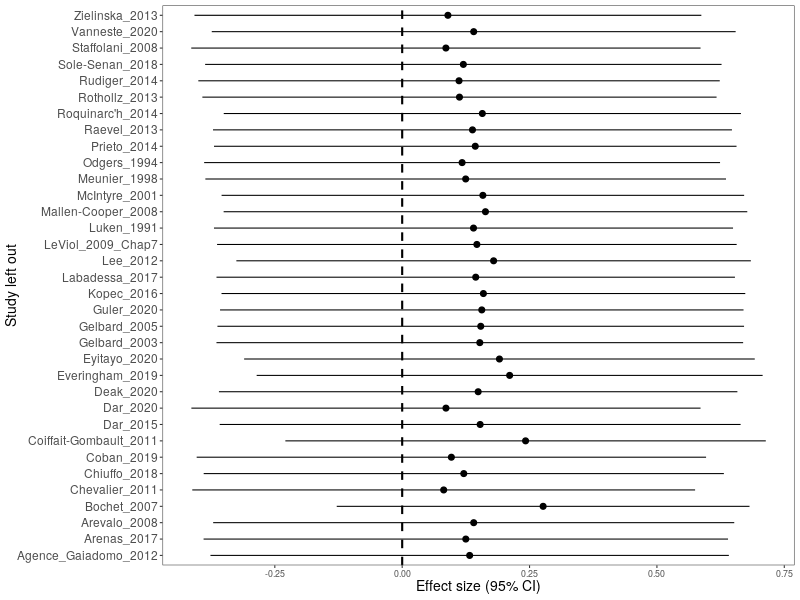
**

**Fig. S3** Leave-one-out analysis for the species richness data showing overall mean effects and 95% CIs based on dataset with one study left out at a time from model fitting.


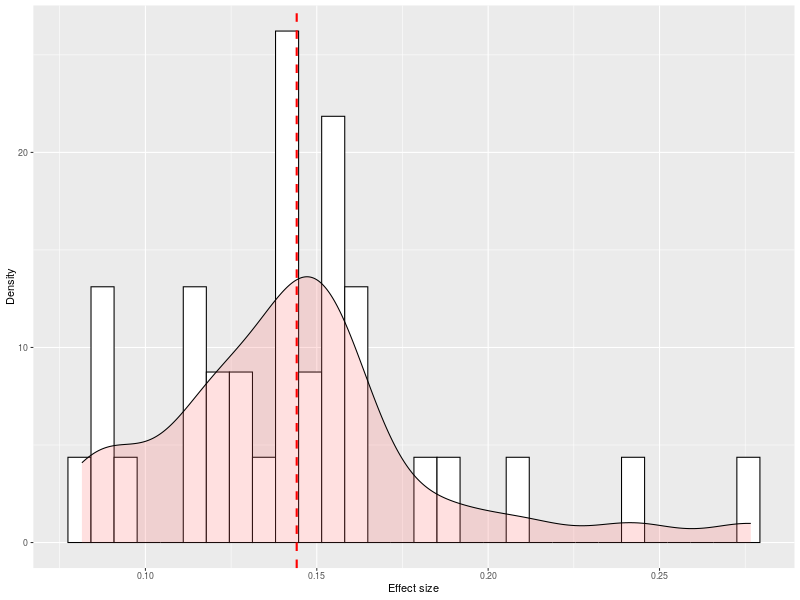
**Fig. S4** Leave-one-out analysis for the abundance species richness showing distribution of overall mean effects based on dataset with one study left out at a time from model fitting.

**2. Meta-regression models summaries**

**Effect of LTI type – Abundance**

Multivariate Meta-Analysis Model (k = 103; method: REML)

Variance Components:

estim sqrt nlvls fixed factor

sigma^2.1 2.3384 1.5292 24 no Author_date

sigma^2.2 2.4874 1.5772 103 no Author_date/Case.ID

Test for Residual Heterogeneity:

QE(df = 99) = 2765.3924, p-val < .0001

Test of Moderators (coefficients 1:4):

QM(df = 4) = 3.0593, p-val = 0.5479

Model Results:

estimate se zval pval

ILT_analysisHighway 0.0029 1.0298 0.0028 0.9978

ILT_analysisNon_Highway_road 0.0132 0.5421 0.0243 0.9806

ILT_analysisPowerline 0.9003 0.6815 1.3210 0.1865

ILT_analysisPipeline -1.7845 1.5570 -1.1461 0.2517

ci.lb ci.ub

ILT_analysisHighway -2.0155 2.0212

ILT_analysisNon_Highway_road -1.0494 1.0757

ILT_analysisPowerline -0.4354 2.2359

ILT_analysisPipeline -4.8361 1.2671

---

Signif. codes: 0 ‘***’ 0.001 ‘**’ 0.01 ‘*’ 0.05 ‘.’ 0.1 ‘ ’ 1

**Effect of LTI type – Species richness**

Multivariate Meta-Analysis Model (k = 78; method: REML)

Variance Components:

estim sqrt nlvls fixed factor

sigma^2.1 1.7094 1.3074 34 no Author_date

sigma^2.2 0.6491 0.8057 78 no Author_date/Case.ID

Test for Residual Heterogeneity:

QE(df = 73) = 1163.7565, p-val < .0001

Test of Moderators (coefficients 1:5):

QM(df = 5) = 2.4166, p-val = 0.7890

Model Results:

estimate se zval pval

ILT_analysisHighway -0.5635 0.6019 -0.9361 0.3492

ILT_analysisNon_Highway_road 0.2867 0.3165 0.9057 0.3651

ILT_analysisPowerline 0.2101 0.6725 0.3124 0.7548

ILT_analysisPipeline -0.5034 0.8885 -0.5665 0.5710

ILT_analysisRailway 0.2620 0.8731 0.3000 0.7641

ci.lb ci.ub

ILT_analysisHighway -1.7433 0.6163

ILT_analysisNon_Highway_road -0.3337 0.9070

ILT_analysisPowerline -1.1080 1.5281

ILT_analysisPipeline -2.2448 1.2380

ILT_analysisRailway -1.4492 1.9732

---

Signif. codes: 0 ‘***’ 0.001 ‘**’ 0.01 ‘*’ 0.05 ‘.’ 0.1 ‘ ’ 1

**Effect of plant status – Abundance**

***Non-highway roads***

Multivariate Meta-Analysis Model (k = 24; method: REML)

Variance Components:

estim sqrt nlvls fixed factor

sigma^2.1 0.4557 0.6751 11 no Author_date

sigma^2.2 0.5316 0.7291 24 no Author_date/Case.ID

Test for Residual Heterogeneity:

QE(df = 22) = 189.3511, p-val < .0001

Test of Moderators (coefficients 1:2):

QM(df = 2) = 9.3835, p-val = 0.0092

Model Results:

estimate se zval pval ci.lb

Status_analysisExotic 0.8025 0.3457 2.3218 0.0202 0.1251

Status_analysisNative -0.3139 0.3222 -0.9740 0.3300 -0.9454

ci.ub

Status_analysisExotic 1.4800 *

Status_analysisNative 0.3177

---

Signif. codes: 0 ‘***’ 0.001 ‘**’ 0.01 ‘*’ 0.05 ‘.’ 0.1 ‘ ’ 1

***Powerlines***

Multivariate Meta-Analysis Model (k = 12; method: REML)

Variance Components:

estim sqrt nlvls fixed factor

sigma^2.1 44.1810 6.6469 3 no Author_date

sigma^2.2 11.7574 3.4289 12 no Author_date/Case.ID

Test for Residual Heterogeneity:

QE(df = 10) = 1528.5160, p-val < .0001

Test of Moderators (coefficients 1:2):

QM(df = 2) = 0.6716, p-val = 0.7148

Model Results:

estimate se zval pval

Status_analysisExotic -4.0289 4.9296 -0.8173 0.4138

Status_analysisNative -0.4107 6.7937 -0.0605 0.9518

ci.lb ci.ub

Status_analysisExotic -13.6908 5.6330

Status_analysisNative -13.7261 12.9046

---

Signif. codes: 0 ‘***’ 0.001 ‘**’ 0.01 ‘*’ 0.05 ‘.’ 0.1 ‘ ’ 1

**Effect of plant status – Species richness**

***Non-highway roads***

***Multivariate Meta-Analysis Model (k = 39; method: REML)***

Variance Components:

estim sqrt nlvls fixed factor

sigma^2.1 0.6538 0.8086 10 no Author_date

sigma^2.2 0.3977 0.6306 39 no Author_date/Case.ID

Test for Residual Heterogeneity:

QE(df = 37) = 302.8747, p-val < .0001

Test of Moderators (coefficients 1:2):

QM(df = 2) = 21.0561, p-val < .0001

Model Results:

estimate se zval pval ci.lb

Status_analysisExotic 0.8793 0.3233 2.7200 0.0065 0.2457

Status_analysisNative -0.2714 0.3215 -0.8443 0.3985 -0.9015

ci.ub

Status_analysisExotic 1.5128 **

Status_analysisNative 0.3586

---

Signif. codes: 0 ‘***’ 0.001 ‘**’ 0.01 ‘*’ 0.05 ‘.’ 0.1 ‘ ’ 1
